# RHCG and TCAF1 promoter hypermethylation predicts biochemical recurrence in prostate cancer patients treated by radical prostatectomy

**DOI:** 10.18632/oncotarget.14391

**Published:** 2016-12-30

**Authors:** Siri H. Strand, Michal Switnicki, Mia Moller, Christa Haldrup, Tine M. Storebjerg, Jakob Hedegaard, Iver Nordentoft, Soren Hoyer, Michael Borre, Jakob S. Pedersen, Peter J. Wild, Jong Y. Park, Torben F. Orntoft, Karina D. Sorensen

**Affiliations:** ^1^ Department of Molecular Medicine, Aarhus University Hospital, Aarhus, Denmark; ^2^ Institute of Pathology, Aarhus University Hospital, Aarhus, Denmark; ^3^ Department of Urology, Aarhus University Hospital, Aarhus, Denmark; ^4^ Institute of Surgical Pathology, University Hospital Zurich, Zurich, Switzerland; ^5^ Department of Cancer Epidemiology, Moffitt Cancer Center, Tampa, Florida, USA

**Keywords:** prostate cancer, DNA methylation, biomarker, diagnosis, prognosis

## Abstract

Purpose: The lack of biomarkers that can distinguish aggressive from indolent prostate cancer has caused substantial overtreatment of clinically insignificant disease. Here, by genome-wide DNA methylome profiling, we sought to identify new biomarkers to improve the accuracy of prostate cancer diagnosis and prognosis.

Experimental design: Eight novel candidate markers, *COL4A6, CYBA, TCAF1* (*FAM115A*)*, HLF, LINC01341* (*LOC149134*), *LRRC4*, *PROM1*, and *RHCG*, were selected from Illumina Infinium HumanMethylation450 BeadChip analysis of 21 tumor (T) and 21 non-malignant (NM) prostate specimens. Diagnostic potential was further investigated by methylation-specific qPCR analysis of 80 NM *vs*. 228 T tissue samples. Prognostic potential was assessed by Kaplan-Meier, uni- and multivariate Cox regression analysis in 203 Danish radical prostatectomy (RP) patients (cohort 1), and validated in an independent cohort of 286 RP patients from Switzerland and the U.S. (cohort 2).

Results: Hypermethylation of the 8 candidates was highly cancer-specific (area under the curves: 0.79-1.00). Furthermore, high methylation of the 2-gene panel *RHCG-TCAF1* was predictive of biochemical recurrence (BCR) in cohort 1, independent of the established clinicopathological parameters Gleason score, pathological tumor stage, and pre-operative PSA (HR (95% confidence interval (CI)): 2.09 (1.26 - 3.46); *P* = 0.004), and this was successfully validated in cohort 2 (HR (95% CI): 1.81 (1.05 - 3.12); *P* = 0.032).

Conclusion: Methylation of the *RHCG-TCAF1* panel adds significant independent prognostic value to established prognostic parameters for prostate cancer and thus may help to guide treatment decisions in the future. Further investigation in large independent cohorts is necessary before translation into clinical utility.

## INTRODUCTION

Prostate cancer is the most commonly diagnosed non-cutaneous malignancy in men in the Western world [1]. The diagnosis is based on elevated serum PSA (prostate-specific antigen), suspicious digital rectal examination and histopathologic evaluation of the sampled biopsies. Unfortunately, the currently available routine prognostic tools (mainly Gleason score (GS), serum PSA, and tumor stage) are unable to clearly distinguish aggressive from indolent disease at the time of diagnosis. Moreover, the lack of biomarkers for aggressive disease, combined with opportunistic PSA screening, has led to large scale overdiagnosis and overtreatment of clinically insignificant prostate cancers, thus new biomarkers are urgently needed.

Methylation of CpG dinucleotides in promoter regions is an essential mechanism of long-term gene silencing. In human malignancies, aberrant hypermethylation of promoter-associated CpG islands (CGIs) is a well-established mechanism for tumor suppressor gene (TSG) silencing [2]. While recurrent somatic mutations are rare in prostate cancer, aberrant promoter hypermethylation occurs early and more consistently in tumor development and progression, and thus constitutes a promising source for discovery of novel biomarkers. Indeed, DNA methylation alterations have shown significant potential as diagnostic as well as prognostic biomarkers for prostate cancer [3].

Here, we performed genome-wide DNA methylation profiling of 21 tumor (T) and 21 non-malignant (NM) prostate tissue specimens using the Illumina Infinium HumanMethylation450 BeadChip (450K array), and selected 8 novel methylation marker candidates associated to the promoter regions of 8 genes for further investigation: *COL4A6* (collagen, type IV, alpha 6)*, CYBA* (cytochrome b-245, alpha polypeptide)*, TCAF1* (*TRPM8* channel-associated factor 1 (previously *FAM115A*))*, HLF* (hepatic leukemia factor)*, LINC01341* (long intergenic non-protein coding RNA 1341 (previously *LOC149134*))*, LRRC4* (leucine rich repeat containing 4)*, PROM1* (prominin 1), and *RHCG* (Rh family, C glycoprotein)*.* Hypermethylation of all candidates was highly cancer-specific in surgical specimens as well as in diagnostic needle biopsy samples (DNBs, area under the curves (AUCs): 0.79-1.00). Finally, a 2-gene panel comprising *RHCG* and *TCAF1* methylation was developed in the training cohort (203 Danish prostate cancer patients) and successfully validated in the validation cohort (286 Swiss/American prostate cancer patients) where it added significant independent prognostic value to routine clinicopathological parameters. This is the first study to show a prognostic biomarker potential for *RHCG* and *TCAF1* methylation in prostate cancer.

## RESULTS

### Identification and validation of candidate methylation markers

**Figure 1:**
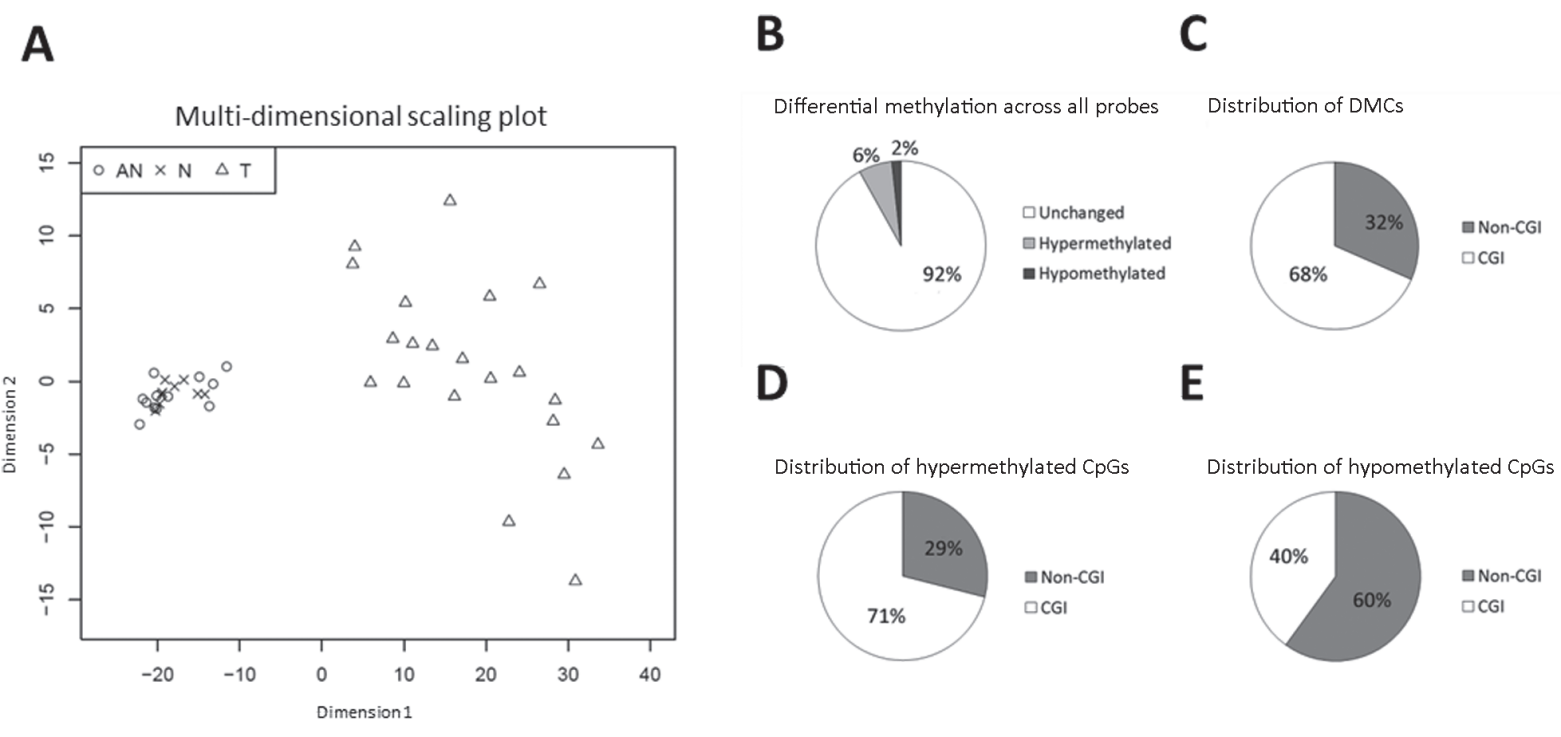
Differential methylation (T ***vs***. **NM) according to Illumina 450K array**. **A**. Multi-dimensional scaling plot of samples included in 450K analysis, based on the 10,000 most variable CpG sites across all samples. T samples (*N* = 21): Triangles; AN samples (*N* = 12): Circles; N samples (*N* = 9): Crosses. **B**.-**E**. Distribution of differential methylation (T *vs*. NM) according to the 450K array. Differentially methylated CpG sites (DMCs) were defined as CpG sites with Δβ ≥ |0.2 | and adj. *P* < 0.05. **B**. Differential methylation across all probes. **C**. Distribution of DMCs. **D**. Distribution of hypermethylated DMCs. **E**. Distribution of hypomethylated DMCs.

To identify novel candidate markers for prostate cancer, global DNA methylation analysis was performed in 21 T, 12 adjacent normal (AN), and 9 true normal (N) prostate tissue samples (Table S1), in addition to 3 malignant (PC3, LNCaP, 22rv1) and 2 NM (PrEC, BPH1) prostate cell lines using the Illumina 450K array. By multi-dimensional scaling analysis, tumor samples showed highly distinct and heterogenous methylation patterns compared to AN and N samples, which clustered tightly together (Figure 1A). No significant differential methylation was observed between AN and N samples (LIMMA differential methylation analysis [4], data not shown), which were thus pooled into one NM sample group. By comparing methylation in T *vs*. NM samples, we identified 37,763 differentially methylated CpG sites (DMCs: mean Δβ ≥ |0.2 |, adj. *P* < 0.05), the majority of which were hypermethylated (*N =* 29,748, Figure 1B) and CGI-associated (Figure 1C, 1D). Conversely, most of the 8,015 DMCs displaying significant hypomethylation in tumors were located outside CGIs (Figure 1E). Thus, we observed cancer-specific hypermethylation of CGIs, whereas CpG sites outside CGIs were largely hypomethylated, consistent with previous reports of methylation patterns in cancer, including prostate cancer [2].

Table 1Clinicopathological characteristics of patients in RP cohorts 1 and 2

**Table:** 

	Cohort 1	Cohort 2
	T	T
*N*	**203**	**286**
Age at RP, median (range)	63 (47 - 77)	61 (41 - 76)
Unknown	0 (0.0 %)	5 (1.7 %)
**Pathological Gleason score**		
<7, *N* (%)	96 (47.3 %)	125 (43.7 %)
=7, *N* (%)	85 (41.9 %)	135 (47.2 %)
>7, *N* (%)	22 (10.8 %)	26 (9.1 %)
**Pathological T-stage (n)**		
≤pT2c, *N* (%)	131 (64.5 %)	216 (75.5 %)
≥pT3a, *N* (%)	72 (35.5 %)	70 (24.5 %)
**Pre-operative PSA**		
PSA ng/ml, median (range)	12.1 (2.0 - 61.0)	6.4 (0.6 - 62.1)
**Surgical margin status**		
Negative, *N* (%)	140 (69.0 %)	72 (25.2 %)
Positive, *N* (%)	63 (31.0 %)	34 (11.9 %)
Unknown, *N* (%)	0 (0.0 %)	181 (63.3 %)
**Lymph node status**		
Positive, *N* (%)	0 (0.0 %)	0 (0.0 %)
Negative, *N* (%)	24 (11.8 %)	23 (8.0 %)
Unknown, *N* (%)	179 (88.2 %)	263 (92.0 %)
**Median follow-up, months (range)**	70 (11 -184)	73(3 - 290)
**PSA recurrence, *N* (%)**	85 (41.9 %)	92 (32.2 %)

**Table:** 

	Cohort 1	
	AN	BPH
*N*	**17**	**13**
Age at RP, median (range)	63 (57-73)	70 (56-83)

**Figure 2:**
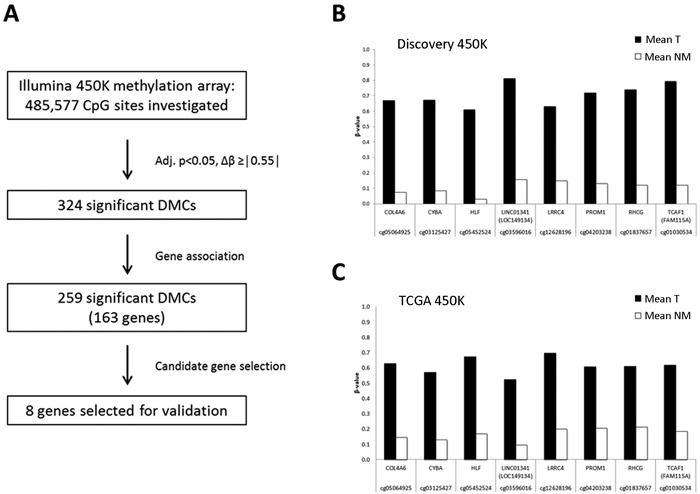
Selection of biomarker candidates **A**: Candidate selection process. Of the 324 top candidate significant DMCs, 259 were associated to a total of 163 different genes. Eight genes were selected for further validation. **B**: Mean methylation of representative DMCs for each selected candidate in T and NM samples according to the discovery 450K dataset (T, *N*=21; NM, *N*=21). **C**: Mean methylation of the same DMCs as in (B) for each candidate in T and NM samples according to the TCGA 450K dataset for prostate cancer (T, *N*=297; NM, *N*=34).” .

To identify candidate biomarkers exhibiting highly cancer-specific differential methylation, we applied a strict threshold (mean Δβ ≥ |0.55|), generating a shortlist of the most differentially methylated CpGs (*N =* 324). Next, filtering for gene association (according to Illumina annotations) generated a final list of 259 top candidate DMCs (adj. *P* < 0.05, mean Δβ >|0.55|), associated to 163 genes (Figure 2A, Table S2). In addition to many novel candidates, this list contained several genes known to be frequently hypermethylated in prostate cancer (*e.g*. *GSTP1, RARB* [5]), supporting the validity of our results. From this list, 8 novel top candidate genes were selected based on their display of highly cancer-specific hypermethylation over multiple adjacent promoter-associated DMCs: *COL4A6, CYBA, HLF, LINC0134* (*LOC149234*)*, LRRC4, PROM1, RHCG,* and *TCAF1* (*FAM115A*) (Figure 2B, S1 and S2, Table S3). None of these candidates have been previously investigated as potential prostate cancer methylation markers.

Table 2Multivariate analysis

**Table:** 

Training cohort (*N* = 203)			
Multivariate			
Variable	HR (95% CI)	*P*	adj. *P*
***RHCG*** **(cont.)**	1.60 (1.07 - 2.38)	**0.021**	**NA**
***TCAF1*** **(cont.)**	2.82 (1.54 -5.17)	**0.001**	**NA**
**Tumor stage (pT2 *vs.* pT3-4)**	2.49 (1.44 - 4.30)	**0.001**	**NA**
**Gleason score (<7 *vs.* ≥7)**	1.86 (1.16 - 2.96)	**0.009**	**NA**
**Pre-op. PSA (<10 *vs*. ≥10)**	3.07 (1.89 - 5.00)	**<0.001**	**NA**
**Surgical margin (neg. *vs.* pos.)**	2.07 (1.27 - 3.37)	**0.003**	**NA**

**Table:** 

Validation cohort (*N* = 286)			
Multivariate			
Variable	HR (95% CI)	*P*	adj. *P*
***RHCG*****(cont.)**	1.55 (1.23 - 1.94)	**<0.001**	**0.001**
**Tumor stage (pT2 *vs.* pT3-4)**	2.03 (1.31 - 3.14)	**0.001**	**0.001**
**Gleason score (<7 *vs.* ≥7)**	2.90 (1.71 - 4.92)	**<0.001**	**<0.001**
**Pre-op. PSA (<10 *vs.* ≥10)**	1.74 (1.13 - 2.67)	**0.011**	**0.011**

**Table:** 

Validation cohort (*N* = 286)			
Multivariate			
Variable	HR (95% CI)	*P*	adj. *P*
***TCAF1*** **(cont.)**	1.48 (1.18 - 1.85)	**0.001**	**0.001**
**Tumor stage (pT2 *vs.* pT3-4)**	2.01 (1.29 - 3.11)	**0.002**	**0.002**
**Gleason score (<7 *vs.*≥7)**	2.85 (1.69 - 4.82)	**<0.001**	**<0.001**
**Pre-op. PSA (<10 *vs.* ≥10)**	1.72 (1.12 - 2.64)	**0.013**	**0.013**

Multivariate Cox regression analysis of methylation marker candidates and clinicopathological variables analyzed as continuous variables with stepwise backwards selection in cohort 1 (top) and of *RHCG* and *TCAF1* in cohort 2 (middle and bottom). Only candidate genes significant in cohort 1 (training) were tested in cohort 2 (validation). Bold: *P*<0.05. NA: Not apliccable.

Table 3Uni- and multivariate Cox regression analysis of the dichotomized *RHCG -TCAF1* panel

**Table:** 

Training cohort (*N* = 203)							
	Univariate			Multivariate			
Variable	HR (95% CI)	*P*	C-index	HR (95% CI)	*P*	C-index^a^	C-index^b^
**d*RHCG-TCAF1***	2.82 (1.74 - 4.59)	**<0.001**	0.581	2.09 (1.26 - 3.46)	**0.004**	**0.777**	
**Tumor stage (pT2 *vs.*pT3-4)**	4.46 (2.86 - 6.97)	**<0.001**	0.677	2.74 (1.66 - 4.55)	**<0.001**		**0.769**			
**Gleason score (<7 *vs.* ≥7)**	2.09 (1.32 - 3.30)	**0.002**	0.579	1.87 (1.18 - 2.97)	**0.007**	
**Pre-op. PSA (< 10 *vs.* ≥ 10)**	2.73 (1.59 - 4.71)	**<0.001**	0.600	2.72 (1.56 - 4.71)	**<0.001**	
**Surgical margin (neg. *vs*. pos.)**	3.47 (2.25 - 5.36)	**<0.001**	0.663	2.49 (1.54 - 4.05)	**<0.001**

**Table:** 

Validation cohort (*N* = 286)							
	Univariate			Multivariate			
Variable	HR (95% CI)	*P*	C-index	HR (95% CI)	*P*	C-index^a^	C-index^b^
**d*RHCG-TCAF1***	2.19 (1.29 - 3.72)	**0.004**	0.550	1.81 (1.05 - 3.12)	**0.032**	**0.717**	
**Tumor stage (pT2 *vs.* pT3-4)**	3.16 (2.09 - 4.78)	**<0.001**	0.629	2.05 (1.33 - 3.17)	**0.001**		**0.703**
**Gleason score (<7 *vs.* ≥7)**	3.53 (2.15 - 5.81)	**<0.001**	0.638	2.81 (1.68 - 4.69)	**<0.001**		
**Pre-op. PSA (< 10 *vs.* ≥ 10)**	2.51 (1.66 - 3.79)	**<0.001**	0.593	1.62 (1.05 - 2.49)	**0.028**		

Analyses performed in cohort 1 (top) and 2 (bottom). Bold: *P*<0.05. ^a^Model including all variables significant in multivariate analysis. ^b^Model including clinicopathological variables only.

For technical validation of the 450K array data, methylation levels of the 8 candidates were investigated by bisulfite sequencing (BS) in the 5 prostate cell lines. BS showed low methylation levels in NM cell lines (PrEC, BPH1) and high levels in malignant cell lines (PC3, LNCaP, 22rv1) for all 8 candidates, and fully corroborated the 450K results for these cell lines (Figure S3). Notably, by both 450K and BS analysis, we observed the same methylation patterns for NM *vs*. malignant cell lines, as was observed for NM *vs*. malignant tissue specimens in the 450K analysis. Moreover, we performed in-silico validation using 450K data from The Cancer Genome Atlas (TCGA) [6, 7] for 297 T and 34 AN prostate specimens, which independently confirmed prostate cancer-specific hypermethylation of all 8 genes (Figure 2C, Table S3). Finally, in order to investigate tissue specificity of our novel candidate markers, we used publicly available 450K methylation array data from the Marmal-aid database [8], focusing on urological cancers and corresponding NM tissue (bladder cancer, *N* = 85; NM bladder, *N* = 10; kidney cancer, *N* = 244; NM kidney, *N* = 136). We found that hypermethylation of *COL4A6, CYBA, LINC01341,* and *RHCG* was highly prostate cancer-specific (Figure S4), suggesting particularly promising diagnostic potential for these genes.

**Figure 3:**
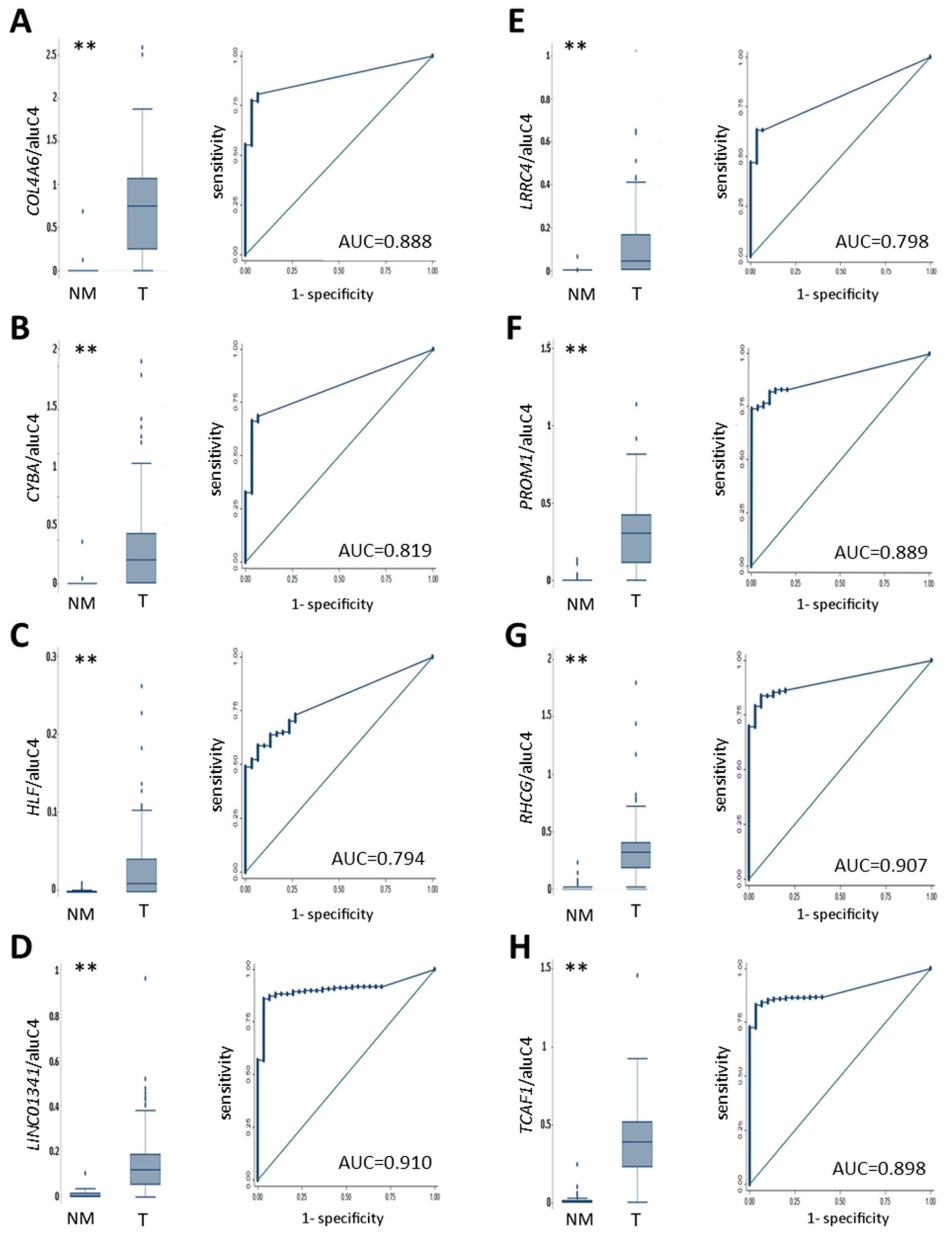
Diagnostic potential of candidate methylation markers in T, AN and BPH samples (cohort 1) ROC analysis of NM samples (AN and BPH, *N* = 30) *vs*. T samples (*N* = 203). Left: Box plots of methylation levels in NM and T samples. (**) *P* < 0.001, rank-sum test. Right: ROC curves of data displayed in box plots. **A**. *COL4A6.* **B**. *CYBA*. **C**. *HLF*. **D**. *LINC01341.* **E**. *LRRC4.* **F**. *PROM1.* **G**. *RHCG.* **H**. *TCAF1*.

**Figure 4:**
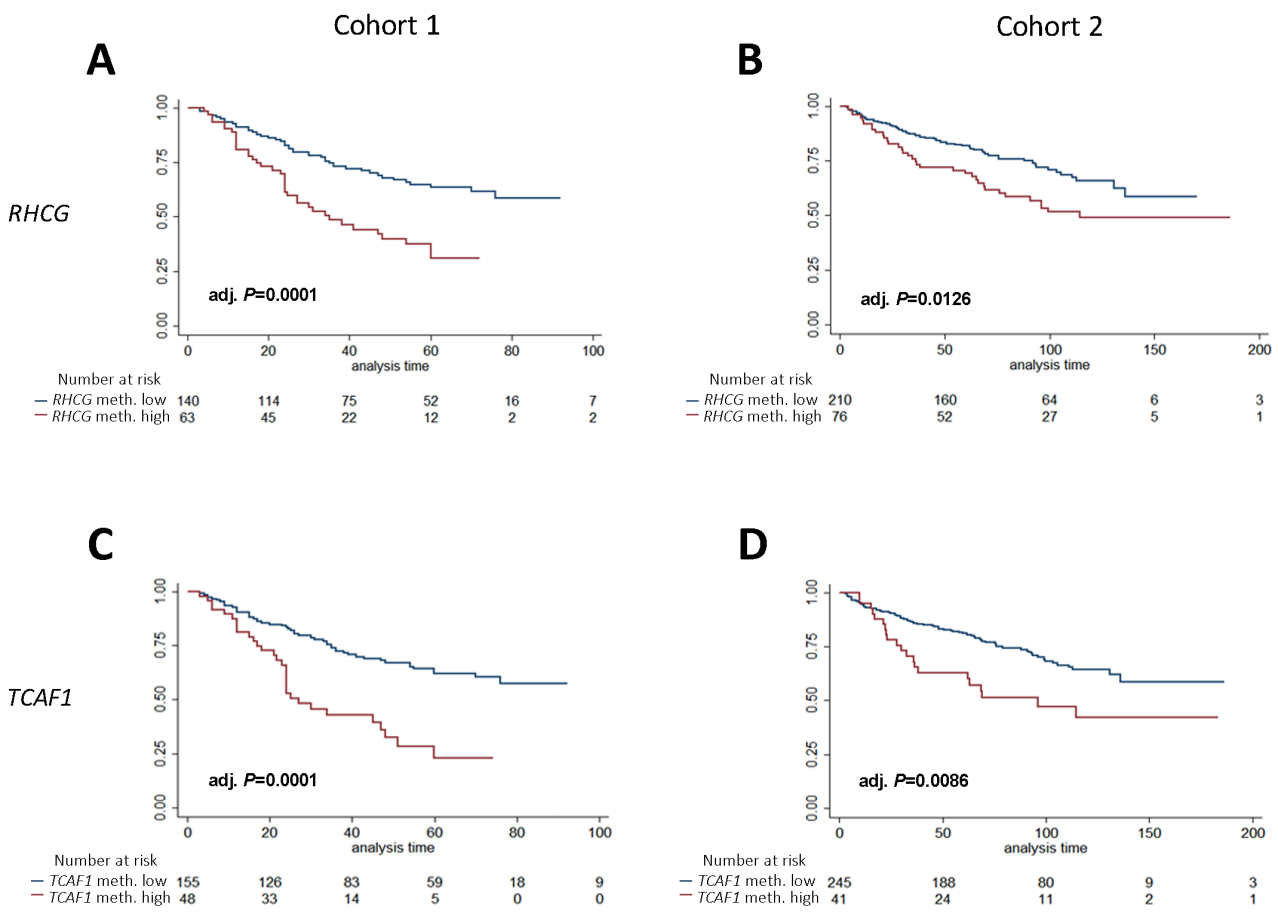
Kaplan-Meier analysis of association between methylation levels of candidate biomarkers and time to BCR after RP Patients were divided into high- or low methylation groups based on ROC analysis of BCR status (36 months after RP, cohort 1). **A, B**: *RHCG* analyzed in cohort 1 (**A**) and cohort 2 (**B**). **C, D**: *TCAF1* analyzed in cohort 1 (**C**) and cohort 2 (**D**). P-values from log-rank test, adjusted according to the Hochberg procedure.

Next, we investigated whether hypermethylation of our candidate biomarkers was associated with altered gene expression, using a small in-house (14 T, 12 NM) and the large TCGA (297 T, 34 NM) RNA-seq datasets. While the non-coding *LINC01341* lacked annotation in both datasets, 6 of the remaining 7 candidate genes were downregulated in T *vs*. NM samples, consistent with epigenetic silencing through aberrant promoter hypermethylation (Figure S5, Table S3). *TCAF1* was not significantly deregulated in the large TCGA dataset, yet we observed a modest but significant upregulation of this transcript in prostate cancer in the small dataset. Notably, aberrant hypermethylation of *TCAF1* was specific to an intragenic CGI/shore region overlapping a putative alternative transcription start site (TSS), suggesting that hypermethylation of this region may stimulate transcription from the upstream TSS (TSS1, Figure S1H) in at least some prostate cancers.

**Figure 5:**
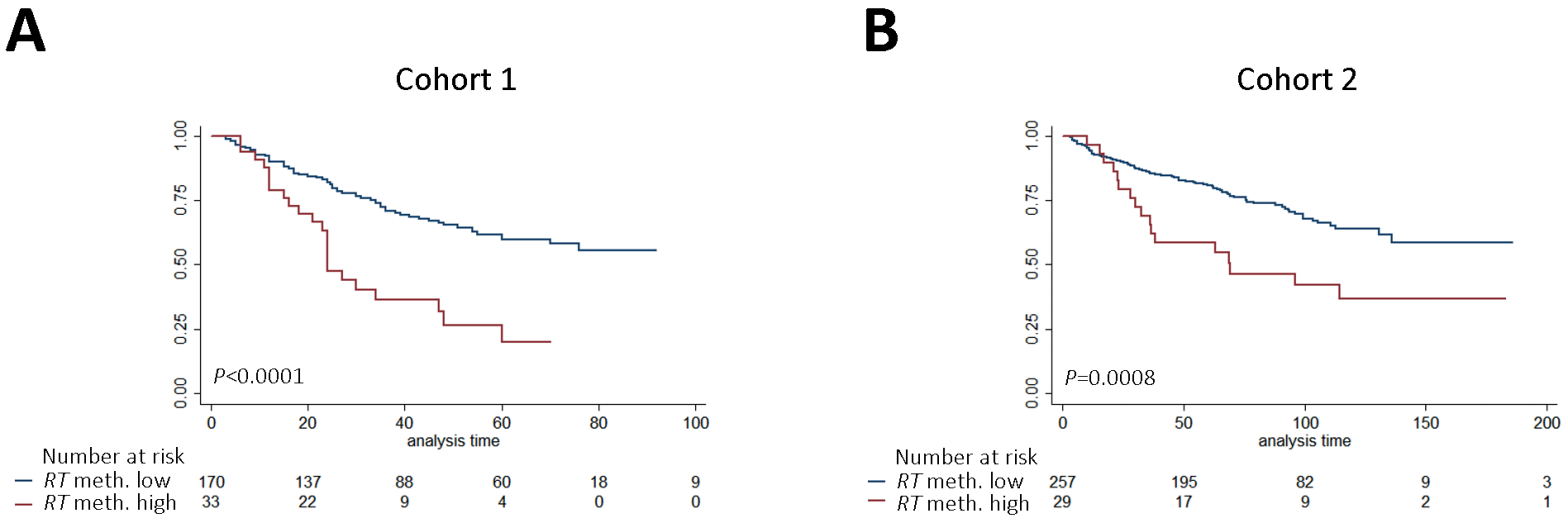
Kaplan-Meier analysis of the association between methylation levels of the marker panel d ***RHCG-TCAF1*** and time to BCR after RP. Patients in cohort 1 (**A**) and cohort 2 (**B**) divided into high-and low methylation groups: High: High methylation of both candidates. Low: High methylation of only one, or low methylation of both candidates. RT: *RHCG-TCAF1* panel. P-values from log-rank test.

Together, these data indicate that *COL4A6, CYBA, HLF, LINC01341*, *LRRC4, PROM1, RHCG,* and *TCAF1* are frequent targets of hypermethylation in prostate cancer, and that aberrant promoter hypermethylation contributes to downregulation of 6 of these genes in this malignancy.

### Diagnostic potential

To investigate the diagnostic potential of our 8 candidates, methylation levels were examined in 203 T and 30 NM prostate tissue samples (cohort 1, Table 1) using methylation-specific qPCR (qMSP). For all 8 loci, T samples displayed highly significant hypermethylation compared to NM samples (Figure 3) with AUCs ranging from 0.79 (*HLF*) to 0.91 (*LINC01341*). At 96.7% fixed specificity (Table S4), sensitivities ranged from 52.2% (*HLF*) to 82.8% (*TCAF1*). To further examine the diagnostic potential, we performed qMSP analysis on 25 malignant and 50 NM (25 AN + 25 N) diagnostic needle biopsy (DNB) specimens. Again, highly significant cancer-specific hypermethylation was observed for all 8 candidates, with AUCs ranging from 0.97 (*HLF*) to 1.00 (*PROM1, TCAF1*) (Figure S6). At 96% fixed specificity (Table S4), sensitivities ranged from 88% (*HLF, LINC01341*) to 96% (*COL4A6, TCAF1, LRRC4*).

**Figure 6:**
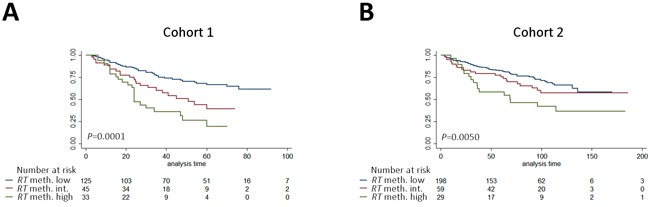
Kaplan-Meier analysis of the association between methylation levels of the marker panel t ***RHCG-TCAF1*** and time to BCR after RP. Patients in cohort 1 (**A**) and cohort 2 (**B**) divided into high-, low- and intermediate methylation groups. High: High methylation of both candidates. Low: Low methylation of both candidates. Intermediate: High methylation of one candidate only. RT: *RHCG-TCAF1* panel. *P*-values from log-rank test.

These results from qMSP analysis of both surgical and DNB specimens confirmed that all 8 genes are highly frequent targets of prostate cancer-specific aberrant hypermethylation, and that our qMSP assays can be used on DNBs.

### Prognostic potential of single candidates

To investigate their possible prognostic potential in prostate cancer, methylation levels of each candidate was initially compared to routine clinicopathological factors in radical prostatectomy (RP) cohort 1, as well as in an independent validation cohort including 286 RP patients (cohort 2, Table 1). For all 8 candidates, a significant correlation between high methylation and at least one established adverse prognostic factor (high GS, advanced pathological tumor stage (pT), high pre-operative PSA) was observed in at least one cohort (Figure S7, Table S5). There were no significant correlations between methylation levels and age (Table S5).

To further assess the prognostic value of the 8 candidates, we investigated whether methylation levels were associated with time to biochemical recurrence (BCR) after RP. Initially, candidate gene methylation (as a continuous variable) was investigated in univariate Cox regression analysis in RP cohort 1. Here, *COL4A6, PROM1, RHCG,* and *TCAF1* were significant predictors of BCR (adj. *P* ≤ 0.006, Table S6). These results were subsequently tested and successfully validated by univariate Cox regression analysis in RP cohort 2 (adj. *P* ≤ 0.008, Table S6). All routine clinicopathological variables were also significant predictors of BCR in univariate Cox regression analyses in both cohorts (Table S6), indicating that these are representative RP cohorts.

Next, in cohort 1, we performed multivariate Cox regression analysis including the 4 candidates significant in univariate analysis, in addition to GS, pT stage, surgical margin (SM) status, and pre-operative PSA. Here, both *RHCG* and *TCAF1* were significant predictors of BCR, independent of routine clinicopathological parameters (*RHCG* hazard ratio (HR) (95% confidence interval (CI)): 1.60 (1.07-2.38), *P =* 0.021; *TCAF1* HR (95% CI): 2.82 (1.54-5.17), *P =* 0.001, Table 2). The independent prognostic potentials of *RHCG* and *TCAF1* were subsequently tested in the validation cohort, where both candidates were significant adverse predictors of BCR, independent of clinicopathological variables (*RHCG* HR (95% CI): 1.55 (1.23 - 1.94), adj. *P =* 0.001; *TCAF1* HR (95% CI): 1.48 (1.18 - 1.85), adj. *P =* 0.001, Table 2). Thus, *RHCG* and *TCAF1* hypermethylation, in addition to routine clinicopathological parameters, were independent predictors of BCR in multivariate analysis in both RP cohorts.

We applied Harrell's C-index to estimate the predictive accuracy of the multivariate model. In cohort 1, adding *RHCG* and *TCAF1* methylation to a model of clinicopathological factors (GS, pT, SM, pre-op. PSA) improved the C-index from 0.769 to 0.782, whereas in cohort 2, adding *RHCG* and *TCAF1* methylation to the clinicopathological model (GS, pT, pre-op. PSA) increased the C-index from 0.703 to 0.718, indicating that *RHCG* and *TCAF1* methylation improved the predictive accuracy in both cohorts and thus carries prognostic potential beyond that of routinely used clinicopathological factors.

In order to simplify test interpretation in the clinic, biomarkers are often analyzed as dichotomized variables. Thus, using cohort 1 for training, *RHCG* and *TCAF1*, respectively, were divided into high- or low- methylation by receiver operating characteristics (ROC) analysis (BCR status at 36 months follow-up) and analyzed for their ability to predict BCR. In cohort 1, high methylation of each candidate significantly predicted BCR in both Kaplan-Meier (adj. *P =* 0.0001, Figure 4), uni- and multivariate Cox regression analyses (adj. *P* < 0.05, Tables S7-S9).

Using the numerical cut-offs defined in cohort 1, we subsequently tested and validated the prognostic potential of dichotomized *RHCG* and *TCAF1* in Kaplan-Meier (adj. *P* < 0.013, Figure 4), uni- and multivariate Cox regression analyses (adj. *P* ≤ 0.04, Tables S7-S9) in cohort 2. Thus, dichotomized *RHCG* and *TCAF1* methylation, in addition to routine clinicopathological parameters, were independent predictors of BCR in both cohorts. Moreover, adding these methylation candidate markers to the clinicopathological models increased the C-indices in both cohorts (Table S8 and S9). Together, these results indicate that dichotomized *RHCG* and *TCAF1* improved the predictive accuracy beyond routine clinicopathological prognostic variables in both cohorts.

### Prognostic methylation panel

Prostate cancer is a highly heterogeneous disease at the molecular level, and multi-gene panels may improve the robustness of individual markers [9]. Thus, we generated a 2-gene panel consisting of dichotomized *RHCG* and *TCAF1* (termed d*RHCG-TCAF1*), where patients with high methylation of both genes were classified into the high-methylation group, and the remaining patients into the low-methylation group. In cohort 1, high methylation of d*RHCG-TCAF1* significantly predicted BCR in univariate (*p* < 0.001, Table 3) and multivariate Cox regression analyses (HR (95% CI): 2.09 (1.26-3.46), *P =* 0.004, Table 3), as well as in Kaplan-Meier analysis (*p* < 0.0001, Figure 5A). The prognostic potential of d*RHCG-TCAF1* was subsequently validated in cohort 2, where it was a significant adverse predictor in both univariate (*P =* 0.004, Table 3) and multivariate (HR (95% CI): 1.81 (1.05-3.12), *P =* 0.032, Table 3) Cox regression analyses, as well as in Kaplan-Meier analysis (*P =* 0.0008, Figure 5B). In cohort 1, the C-index increased from 0.769 to 0.777 when d*RHCG-TCAF1* methylation was added to the clinicopathological model, whereas in cohort 2, it increased from 0.703 to 0.717 by addition of d*RHCG-TCAF1* methylation, indicating improved model performance by adding the 2-gene panel to the established prognostic parameters. Thus, d*RHCG-TCAF1* methylation, together with all available clinicopathological parameters, independently predicted BCR in multivariate analysis in both RP cohorts.

Next, we investigated the prognostic potential of a trichotomized methylation model (t*RHCG-TCAF1*), where patients in cohort 1 were stratified into 3 groups based on whether they had high methylation of both candidates (high-methylation), one candidate (intermediate-methylation), or neither candidate (low-methylation). By Kaplan-Meier analysis, patients in the high-methylation group had significantly higher risk of BCR than patients in the 2 remaining groups, where patients with low methylation also had the lowest risk of BCR (*P =* 0.0001, Figure 6A). This was successfully validated in cohort 2 (*P =* 0.0050, Figure 6B). Moreover, when comparing the high and low-methylation groups only, high methylation was a significant predictor of BCR in uni- and multivariate Cox regression analyses in cohort 1 (Table S10), and these results were successfully validated in cohort 2 (Table S10), suggesting that prostate cancer patients can be stratified into clinically relevant subgroups based on *RHCG* and *TCAF1* methylation. Notably, 3 years after surgery, 61% of patients with high methylation of the *RHCG-TCAF1* panel had suffered BCR, compared to 23% of patients with low methylation in cohort 1. Likewise, in cohort 2, 35% of patients with high *RHCG-TCAF1* methylation had suffered BCR 3 years after surgery, compared to 23% of patients with low methylation. In summary, these results suggest that high methylation of the 2-gene panel *RHCG-TCAF1* is a significant adverse predictor of BCR after RP, independent of routine clinicopathological parameters.

## DISCUSSION

Here, we identified *COL4A6, CYBA, HLF, LINC01341, LRRC4, PROM1, RHCG,* and *TCAF1* as novel targets of frequent hypermethylation in prostate cancer. Cancer-specific hypermethylation was observed for all candidates in both surgical and DNB specimens, suggesting promising diagnostic biomarker potential. Furthermore, we generated a new 2-gene panel comprising *RHCG* and *TCAF1* methylation, which predicted BCR in two RP cohorts from Denmark, Switzerland, and the U.S. independently of routine clinicopathological parameters. This is the first study to demonstrate a significant prognostic value of *RHCG* and *TCAF1* hypermethylation in prostate cancer.

In addition to technical validation by BS, hypermethylation in prostate cancer of our eight candidates was confirmed in the large external TCGA dataset (450K), further supporting their diagnostic potential. Moreover, as proof of principle, we demonstrated that our qMSP assays can be used on DNBs despite scarce sample amounts. While a molecular diagnostic test that can detect histologically verified prostate cancer in DNBs might have limited clinical utility, detection of methylation-based cancer field effects in morphologically normal prostate biopsies may be used to guide the need for repeat biopsy in men with exclusively cancer-negative DNBs but persistently elevated PSA, which remains a major clinical challenge [10, 11]. Although we did not detect significant differential methylation between AN and N samples in this study, our analysis could be limited by the relatively small sample size. Further studies are needed to investigate the possible existence of epigenetic cancer field effects for our candidate genes. Future studies should also investigate whether our novel candidate markers are detectable in blood or urine samples, *e.g*. as circulating cell-free tumor DNA methylation, which could facilitate development of minimally or non-invasive testing for prostate cancer.

Here, using two large prostate cancer patient cohorts, we identified and independently validated *RHCG*, *TCAF1*, and the 2-gene panel *RHCG-TCAF1* as novel independent adverse predictors of BCR after RP. Prior to this, only a few candidate methylation markers have demonstrated prognostic potential in more than one prostate cancer cohort by multivariate analysis adjusting for established clinicopathological parameters [3]. These are *PITX2* [12, 13], *GABRE~miR-452~miR-224* [14], *C1orf114* (*CCDC181*) [9], and the panels *C1orf114/HAPLN3* and *AOX1/C1orf114/HAPLN3* [9]. Further studies, using large independent cohorts with long follow-up, are needed to assess the clinical utility of *RHCG* and *TCAF1* as well as of the previously identified prognostic methylation marker candidates. These candidates should be investigated individually, as well as in combinations, to identify their true prognostic potential.

Current prognostic prostate cancer classification systems rely on histopathological criteria, which cannot accurately predict whether a tumor will progress to clinically relevant disease or remain indolent. Novel biomarkers that enable distinction between aggressive and indolent cancer at the time of diagnosis could improve patient management significantly, *e*.*g*. by allowing active surveillance of low-risk patients and immediate treatment of high-risk patients. While the incremental gain in prognostic information obtained by adding parameters to existing models is often modest [15], the increased C-indices presented here nevertheless represent meaningful improvements to the model comprising only clinicopathological parameters [16]. The full multivariate model including the dichotomized methylation marker panel d*RHCG*-*TCAF*1 provided a C-index of 0.777 in cohort 1 and 0.717 in cohort 2. C-index analysis in reduced models, *i.e*. leaving out one variable at a time, demonstrated modest C-index contributions for any single variable (range 0.008-0.028 in cohort 1 and 0.011-0.032 in cohort 2), corresponding to a maximum contribution of 3.6% and 5.2% in cohort 1 and 2, respectively (data not shown). Moreover, the observed increments in C-index for our novel 2-gene methylation marker panel are comparable to those reported for other prognostic DNA methylation marker candidates [9, 13, 14], as well as for prognostic gene expression signatures that have been developed into commercial tests for prostate cancer, *i.e*. Decipher [17], Oncotype [18] and Prolaris [19].

Furthermore, as cancer-specific hypermethylation was detectable in DNBs, our results suggest that *RHCG-TCAF1* has the potential to improve the accuracy of prostate cancer prognosis at the time of diagnosis, where only pre-operative clinicopathological parameters are available (biopsy-based GS, clinical tumor stage, and pre-operative PSA). Notably, more than 50% of prostate tumors are upstaged and/or upgraded after RP [20], further stressing the need for improved prognostic markers at an early stage. Thus, it is likely that our methylation marker candidates would contribute relatively more independent prognostic information in biopsy specimens at the time of diagnosis, than shown here for RP samples, and thus potentially could be used to guide treatment decisions in the future. Moreover, our qMSP assays are simple and cost-effective and as such would be easy to incorporate into routine diagnostic practice.

The potential functional roles of our 8 candidate methylation markers in prostate physiology and malignancy are largely unknown. Here, we found that expression of 6 of these genes was significantly downregulated in prostate cancer, consistent with epigenetic silencing and suggesting a possible function in prostate carcinogenesis and/or tumor progression. More specifically, we observed promoter hypermethylation and downregulation of *RHCG* in prostate tumors, which is consistent with previous reports of *RHCG* downregulation in kidney and oesophagal carcinomas [21, 22]. *RHCG* encodes an epithelial ammonia transporter that is widely expressed in the kidney, liver and intestinal tract, as well as in male reproductive organs, where it contributes to multiple components of fertility [23]. The role of *RHCG* in the normal and cancerous prostate, however, is unknown and further studies are warranted.

*TCAF1* was recently identified as a positive regulator of *TRPM8*, an ionotropic testosterone receptor highly expressed in various organs, including the prostate [24, 25], but its exact function in the prostate is unknown [25, 26]. According to our analysis, *TCAF1* expression was unaltered or modestly upregulated in localized prostate tumors compared to NM prostate tissue samples, consistent with a previous study [24]. Several studies have reported that intragenic DNA methylation, as we observed for *TCAF1* in prostate cancer, is involved in alternative TSS-regulation in normal and malignant tissue [27]. Moreover, gene body methylation is a feature of actively transcribed genes [28]. Future studies should investigate *TCAF1* methylation and potential isoform-specific expression patterns in NM and malignant prostate cells to elucidate its possible role in prostate cancer tumorigenesis and progression.

We observed cancer-specific promoter hypermethylation and downregulation of *PROM1* (*CD133*) expression, which is consistent with reports of *PROM1* downregulation in several malignancies, including prostate cancer [29, 30]. *PROM1* encodes a transmembrane glycoprotein widely used as a stem cell marker, but its potential role in prostate cancer is unknown. We also observed cancer-specific promoter hypermethylation and downregulation of *COL4A6,* which encodes a subunit of the epithelial basement membrane protein collagen IV. Our observations are consistent with reports of *COL4A6* downregulation in a range of cancers, including prostate cancer [31]. Cancer-specific hypermethylation and transcriptional downregulation was also observed for *CYBA, HLF*, and *LRRC4. CYBA* encodes a subunit of an NADPH oxidase, whereas *HLF* encodes a transcriptional activator of the proline and acidic-rich (PAR) protein family. *LRRC4* is a proposed TSG involved in nervous system development and differentiation [32], and was reported to be hypermethylated and downregulated in gliomas [33]. However, the roles of these genes in prostate physiology and malignancy are unknown. Finally, we observed cancer-specific promoter hypermethylation of the verified but uncharacterized long non-coding RNA *LINC01341* [34]. Unfortunately, no expression data was available for *LINC01341* in the investigated datasets. Thus, further studies are needed to elucidate the possible role of *LINC01341* in normal and malignant prostate biology.

There are some limitations to the present study. The prognostic analyses for our candidates were based on RP specimens, and while it could be useful to identify high-risk patients in need of adjuvant therapy post-RP (*e.g*. radiation treatment or androgen deprivation), there are currently no established adjuvant lines of treatment for patients after RP. Thus, future studies should evaluate the prognostic potential of these novel candidate methylation markers in DNBs, and thus investigate whether they can predict prostate cancer aggressiveness at the time of diagnosis and thereby help guide treatment decisions.

Moreover, the RP cohorts used for analysis of prognostic biomarker potential were of moderately different compositions, with patients in cohort 1 suffering more BCRs and having tumors with higher pT stage and higher median pre-operative PSA compared to patients in cohort 2 (Table 1). Nevertheless, all routine clinicopathological variables (GS, pT, pre-op. PSA) were significant predictors of BCR in uni- and multivariate Cox regression analysis in both cohorts, strongly indicating that both constitute representative RP cohorts. Moreover, our *RHCG-TCAF1* panel was significant in multivariate analysis in both cohorts, using the exact numerical dichotomization/trichotomization cut-points derived from cohort 1 (training cohort), thus indicating the robustness of our methylation marker candidates.

Another limitation to our study is the lack of SM status information for most patients in cohort 2, thus this parameter was excluded from multivariate analysis in this cohort. Nevertheless, SM status was a significant predictor of BCR in uni- and multivariate Cox regression analysis in cohort 1, as were our methylation marker candidates, further supporting their strength as independent prognostic biomarkers for prostate cancer. Furthermore, the use of BCR as end-point for survival analysis constitutes another possible limitation. BCR may result from either non-radical surgery, or micro metastatic disease manifesting prior to surgery, and is only a surrogate marker for tumor aggressiveness. Thus, future studies should investigate our novel methylation marker candidates in relation to more clinically relevant endpoints, such as metastatic progression or prostate cancer-specific mortality. Due to the slow progression of prostate cancer, such studies would require large cohorts with > 15 years of follow-up [35]. Finally, while the cohorts used in this study included patients of European descent, further studies are needed to investigate if our findings can be extended to other ethnic groups.

In conclusion, we identified and validated 8 novel methylation marker candidates for prostate cancer diagnosis. We also identified and validated the prognostic potential of a new 2-gene methylation marker panel *TCAF1-RHCG*, which predicted time to BCR independently of established clinicopathological parameters in 2 RP cohorts. The actual clinical utility of these novel candidate methylation markers for prostate cancer diagnosis and prognosis should be further investigated in large independent cohorts with long follow-up and clinically relevant end-points. Moreover, an important future task will be to investigate whether methylation of *RHCG* and *TCAF1* can also predict prostate cancer aggressiveness at the time of diagnosis based on analysis of DNBs or even liquid biopsies, in order to guide treatment decisions.

## MATERIALS AND METHODS

### Infinium HumanMethylation450 BeadChip

A total of 21 T, 14 AN, 9 N prostate tissue samples (Table S1), and 5 cell lines (BPH1, 22rv1, LNCaP, PC3, and PrEC; Table S11) were analyzed by the Infinium HumanMethylation450 BeadChip (Illumina, San Diego, CA). Bisulfite conversion and genome-wide methylation analysis was conducted as a commercial service by The Genome Centre, Barts and the London School of Medicine and Dentistry, London, UK, according to manufacturer's protocol. For further details, see supplementary methods.

### Quantitative methylation specific PCR (qMSP)

#### Patient material

*Biopsy sample set*: DNB specimens (formalin-fixed, paraffin-embedded (FFPE)) from patients undergoing transrectal ultrasound-guided biopsy due to suspicion of prostate cancer were obtained from Dept. of Pathology, Aarhus University Hospital, DK, as described elsewhere (Moller et al., Scientific Reports, in press). We obtained normal (N) biopsy samples from 25 patients with exclusively cancer-negative biopsies, tumor biopsies from 25 patients diagnosed with prostate cancer (T), and histologically normal (AN) biopsies from 25 patients with prostate cancer detected in other biopsies.

*Radical prostatectomy cohorts*: Cohort 1 (training cohort) consisted of 566 curatively intended RP patients with histologically verified clinically localized prostate cancer (FFPE) from the Dept. of Urology, Aarhus University Hospital, DK (collected 1997-2009). All specimens were assessed by a trained pathologist. Punch biopsies were obtained from FFPE block-areas with > 80% cancer cells, as described previously [9, 14, 36]. Of the 566 patients, 1 withdrew consent, 37 were either lost to follow-up, had < 3 months follow-up, or suffered BCR within 3 months post-RP, 42 were excluded because of pre-/postoperative endocrine treatment or missing data for endocrine treatment, 6 were excluded due to positive lymph node status, 1 lacked clinical data, and 240 were excluded due to lack of tissue in the FFPE block or insufficient DNA concentration/quality. Final analyses comprised 203 prostate cancer samples (Table 1).

Cohort 2 consisted of 448 and 117 curatively intended RP patients with histologically verified, clinically localized prostate cancer (FFPE) from Moffitt Cancer Center, U.S. (collected 1987-2006) and University Hospital Zurich, Switzerland (collected 1993-2001). Of the 565 patients, 19 were either lost to follow-up, had < 3 months follow-up, or suffered BCR within 3 months post-RP, 82 were excluded because of pre-/postoperative endocrine treatment or missing data for endocrine treatment, 6 were excluded due to positive lymph node status, 13 were excluded due to ethnicity (non-European descent), 118 lacked clinical data, and 41 lacked tissue in the FFPE block or had insufficient DNA concentration/quality. Final analyses comprised 286 samples (Table 1). See Figure S8A for flow chart of inclusion/exclusion criteria for both cohorts according to REMARK guidelines [37]. The compositions of the final cohorts were similar to that of the original cohorts (Table S12).

FFPE AN tissue samples from RP specimens and benign prostate hyperplasia (BPH) specimens (trans-urethral resection of the prostate) were sampled as described above for cohort 1 (Table 1, Figure S8B).

#### DNA purification and bisulfite conversion

For DNA purification from DNBs, 3-µm tissue sections were deparaffinized and DNA was extracted using the QIAamp DNA FFPE Tissue Kit (Qiagen) according to manufacturer's protocol. DNA from RP specimens (punch biopsies) was extracted in Denmark and Switzerland using gDNA Eliminator columns from the RNeasy plus micro kit (Qiagen) and the blood and cell culture DNA kit (Qiagen), respectively, as previously described [9]. DNA from FFPE samples from the U.S. was extracted using the QIAamp DNA FFPE tissue kit (Qiagen). All DNA samples were bisulfite-converted using the EZ-96 DNA Methylation-Gold KitTM (Zymo research).

qMSP assays (Table S13) were designed using Primer3Plus [38] and Beacon DesignerTM (Premier Biosoft) and run as previously described [9]. Briefly, 5 ng bisulfite-converted DNA was analyzed per reaction. DNA and mastermix (Taqman universal mastermix no UNG and primer/probe sets) were run in 384-well plates on the Applied Biosystems 7900HT real-time thermal cycler. Bisulfite-converted and un-converted CpGenome Universal Methylated DNA (Millipore), 2 negative controls (whole-genome amplified DNA and H_2_O), as well as serially diluted methylated DNA samples for standard curve analysis, were included on each plate. All reactions were run in triplicates. *MYOD1* and aluC4 served as controls for DNA quality/quantity [9, 39], and aluC4 was used for normalization. Samples were excluded from further analysis if ≥ 2 aluC4 reactions had Ct > 25 and/or *MYOD1* was not amplified. Samples were considered negative for methylation if ≥ 2 methylation-specific reactions did not amplify. Bisulfite-converted DNA from DNBs was pre-amplified prior to qMSP (Table S13).

### Statistics

Statistical analyses were performed using STATA v. 11.2 (StataCorp, College Station, TX, USA). Associations between DNA methylation and clinicopathological variables were assessed by Wilcoxon rank-sum test and Spearman correlations. BCR (defined as PSA ≥ 0.2 ng/ml) was the clinical endpoint in survival analyses. Patients without BCR were censored at their last normal PSA measurement. In all cases, *p* < 0.05 was considered significant. Where appropriate, correction for multiple testing was conducted according to Hochberg [40]. The prognostic potential of methylation marker candidates was analyzed by uni- and multivariate Cox regression analysis, Kaplan-Meier analysis and two-sided log-rank tests. Predictive accuracy was estimated using Harrell's C-index [41]. For multivariate testing, all clinicopathological parameters significant in univariate analysis were included. SM status was unavailable for U.S. patients, and was therefore excluded from multivariate analysis in cohort 2.

Prognostic 2-gene model: For each gene, patients in cohort 1 (training cohort) were dichotomized into high/low-methylation groups by ROC analysis of BCR status at 36 months follow-up. Cohort 2 patients were dichotomized by the exact numerical cutoff values defined in cohort 1 (*RHCG*: ≥ 0.3608787; *TCAF1*: ≥ 0.519267). For the dichotomized 2-gene panel (d*RHCG-TCAF1*), patients were included in the high-methylation group if both genes were highly methylated. For the trichotomized 2-gene panel (t*RHCG-TCAF1*), patients were included in the high-methylation group if both genes were highly methylated, the intermediate group if one gene was highly methylated, and the low-methylation group if neither gene was highly methylated.

### Bisulfite sequencing, RNA-seq, external datasets

See Supplementary Materials and Methods.

### Data and materials availability

Data is available upon request.

## SUPPLEMENTARY MATERIALS FIGURES AND TABLES





## Abbreviations

450K: Illumina Infinium HumanMethylation450 BeadChip
AN: Adjacent normal
AUC: Area under the curve
BCR: Biochemical recurrence
BPH: Benign prostate hyperplasia
BS: Bisulfite sequencing
CGI: CpG island
CI: Confidence interval
DMC: Differentially methylated CpG site
DNB: Diagnostic needle biopsy
FFPE: Formalin-fixed, paraffin-embedded
GS: Gleason score
N: Normal
NM: Non-malignant
PSA: Prostate-specific antigen
pT: Pathological tumor stage
qMSP: Methylation-specific qPCR
ROC: Receiver operating characteristics
RP: Radical prostatectomy
SM: Surgical margin
TCGA: The Cancer Genome Atlas
TSG: Tumor suppressor gene
TSS: Transcription start site
