# Supplementary material for: RHCG and TCAF1 promoter hypermethylation predicts biochemical recurrence in prostate cancer patients treated by radical prostatectomy
Source: Oncotarget. 2016 Dec 30;8(4):5774–88. doi: 10.18632/oncotarget.14391 (PMC5351588; doi:10.18632/oncotarget.14391)
Supplement: Supplementary file 1 [file oncotarget-08-5774-s001.pdf]

RHCG and *TCAF1* promoter hypermethylation predicts biochemical recurrence in prostate cancer patients treated by radical prostatectomy

Supplementary Materials and Methods

Infinium HumanMethylation450 BeadChip

Patient material: Fresh-frozen tissue samples were collected at Dept. of Urology, Aarhus University Hospital, DK (2004-2011). Malignant prostate tissue (T) was obtained from 20 clinically localized prostate cancers removed by RP and one transurethral resection of the prostate. Histologically benign prostate tissue samples (adjacent normal, AN) were obtained from 12 RP patients, and 9 true normal (N) prostate tissue samples from age-matched bladder cancer patients (cystoprostatectomy). Absence of cancer in AN and N samples was confirmed by a trained pathologist. Clinical data is listed in table S1.

Cell lines: Malignant/NM prostate epithelial cell lines were maintained according to providers' protocols (table S11) and cultured to ~80 % confluence before harvesting. Cells were tested negative for mycoplasma shortly before harvesting (PCR Mycoplasma Detection Set, Takara Bio Inc.), and cell line identity was confirmed by short tandem repeat (STR) analysis (IdentiCell).

DNA purification: Genomic DNA (gDNA) was extracted from carefully selected macro dissected 20 µm sections of fresh frozen TissueTek (Sakura, Torrance, CA) embedded prostate tissue samples and cell lines using the PUREGENE DNA purification kit (Gentra systems) with proteinase K treatment (100 U, 30 min at 55 °C), as previously described [1]. Cell line gDNA was extracted after cell harvesting by Trypsin-EDTA (Invitrogen) for all cells except PrEC, which were harvested using Trypsin-EDTA for primary cells (ATCC) and Trypsin Neutralizing Solution (ATCC) according to manufacturer's protocol.

450K data handling and statistical analysis: The 450K array investigates DNA methylation at 485,577 CpG sites in the human genome at single base resolution. Each investigated CpG is assigned a β-value ranging from 0 (unmethylated) to 1 (fully methylated). Raw data was processed through the minfi package [2],

followed by statistical analysis on logit-transformed [3], peak-corrected [4] β-values in R [5] using LIMMA [6] (t-statistics) to identify differential methylation between T and NM samples (Δβ: mean β_T - mean β_{NM}). N and AN samples were pooled into one control group (NM) for further analysis, as analysis for differential methylation (LIMMA applied to peak-corrected M-values) revealed highly similar methylation patterns for the two groups. All samples passed the inclusion criteria of a detection *P*-value $<10^{-5}$. Multi-dimensional scaling analysis was performed using the 10,000 most variable CpG sites across all tissue samples. Correction for multiple testing (adjusted *P*-value, adj. *P*) was performed according to the Benjamini-Hochberg procedure [7]. Differentially methylated CpG sites (DMCs) were defined as CpG sites with $\Delta\beta \geq |0.2|$ and adj. $P<0.05$.

Bisulfite sequencing

Bisulfite sequencing (BS) of genomic DNA from prostatic cell lines was performed as previously described [8]. Briefly, primers (table S13) were designed using MethPrimer [9]. Bisulfite converted DNA was PCR amplified, gel purified and sub-cloned using the TOPO®TA Cloning® Kit for sequencing (Invitrogen). A minimum of 5 colonies were PCR amplified and sequenced using the M13 forward and reverse primers (included in the TOPO cloning kit), followed by manual inspection of C/T status at each CpG site. Results were visualized and analyzed using QUMA [10].

RNA-seq

A total of 14 T, 6 N, and 6 AN fresh-frozen prostate tissue samples were collected at Dept. of Urology, Aarhus University Hospital, DK (2004-2011). Of these, 6 T samples were also included in a previous RNA-seq study [11]. Total RNA was isolated from tissue samples using the RNeasy Mini Kit (Qiagen) according to manufacturer's instructions, except that at the time of extraction, 1.5x (vol.) 100% EtOH was added to the tissue samples. All included samples had a RIN score >7, according to RNA Pico chip analysis on a 2100 Bioanalyzer

(Agilent Technologies). Whole transcriptome, strand-specific RNA-seq libraries for multiplexed paired-end sequencing were prepared using Ribo-Zero Gold and ScriptSeq v2 kit (Epicentre), as previously described [11]. Di-tagged cDNA was amplified (10 cycles, FailSafe PCR Enzyme (Epicentre)) and purified (Agencourt XP Kit, Beckman Coulter). RNA-seq libraries were combined into 2 nM pooled stocks, denatured and diluted to 10 pM before loading into TruSeq PE v3 flowcells on an Illumina cBot followed by indexed paired-end sequencing (101+7+101 bp) on Illumina HiSeq 2000 using TruSeq SBS Kit v3 chemistry (Illumina). Paired de-multiplexed fastq files were generated using CASAVA software (Illumina).

RNA-seq data handling and statistical analysis: Paired-end RNA-seq reads were mapped to the human genome (hg19) using TopHat [12] with the Bowtie aligner [13]. HTSeq [14] was used to summarize reads per gene of interest with the "union" overlap resolution mode. Differential expression analysis was performed using edgeR [15] with the most complex dispersion found for each gene. Correction for multiple testing (adjusted *P*-value, adj. *P*) was performed according to the Benjamini-Hochberg procedure [7].

External datasets

450K and RNA-seq data sets (297 T, 34 AN) from The Cancer Genome Atlas (TCGA) were downloaded from the TCGA data portal [16, 17] and processed as described above.

Marmal-aid data was downloaded from the Marmal-aid database [18]. Raw beta-values were batch- and peak corrected using ChAMP [19]. Methylation in malignant and NM bladder (cancer: N= 85. NM: N= 10) and kidney (cancer: N=244. NM: N=136) tissue was assessed by calculating the mean β-values for the CpG sites identified as hypermethylated in PC (probe IDs listed in supplementary table S3) for each candidate gene in each cancer type and corresponding NM tissue.

References

1. Sorensen KD, Borre M, Orntoft TF, Dyrskjot L, Torring N. Chromosomal deletion, promoter hypermethylation and downregulation of FYN in prostate cancer. Int J Cancer. 2008; 122: 509-19. doi: 10.1002/ijc.23136.
2. Aryee MJ, Jaffe AE, Corrada-Bravo H, Ladd-Acosta C, Feinberg AP, Hansen KD, Irizarry RA. Minfi: a flexible and comprehensive Bioconductor package for the analysis of Infinium DNA methylation microarrays. Bioinformatics. 2014; 30: 1363-9. doi: 10.1093/bioinformatics/btu049.
3. Du P, Zhang X, Huang CC, Jafari N, Kibbe WA, Hou L, Lin SM. Comparison of Beta-value and M-value methods for quantifying methylation levels by microarray analysis. BMC Bioinformatics. 2010; 11: 587. doi: 10.1186/1471-2105-11-587.
4. Dedeurwaerder S, Defrance M, Calonne E, Denis H, Sotiriou C, Fuks F. Evaluation of the Infinium Methylation 450K technology. Epigenomics. 2011; 3: 771-84. doi: 10.2217/epi.11.105.
5. The R project for statistical computing. (https://www.r-project.org/).
6. Jeanmougin M, de Reynies A, Marisa L, Paccard C, Nuel G, Guedj M. Should we abandon the t-test in the analysis of gene expression microarray data: a comparison of variance modeling strategies. PLoS One. 2010; 5: e12336. doi: 10.1371/journal.pone.0012336.
7. Benjamini Y, Hochberg Y. Controlling the False Discovery Rate: A Practical and Powerful Approach to Multiple Testing. Journal of the Royal Statistical Society Series B (Methodological). 1995; 57: 289-300. doi:
8. Vestergaard EM, Nexo E, Torring N, Borre M, Orntoft TF, Sorensen KD. Promoter hypomethylation and upregulation of trefoil factors in prostate cancer. Int J Cancer. 2010; 127: 1857-65. doi: 10.1002/ijc.25209.
9. Li LC, Dahiya R. MethPrimer: designing primers for methylation PCRs. Bioinformatics. 2002; 18: 1427-31. doi:
10. Kumaki Y, Oda M, Okano M. QUMA: quantification tool for methylation analysis. Nucleic Acids Res. 2008; 36: W170-5. doi: 10.1093/nar/gkn294.
11. Hedegaard J, Thorsen K, Lund MK, Hein AM, Hamilton-Dutoit SJ, Vang S, Nordentoft I, Birkenkamp-Demtroder K, Kruhoffer M, Hager H, Knudsen B, Andersen CL, Sorensen KD, et al. Next-generation sequencing of RNA and DNA isolated from paired fresh-frozen and formalin-fixed paraffin-embedded samples of human cancer and normal tissue. PLoS One. 2014; 9: e98187. doi: 10.1371/journal.pone.0098187.
12. Trapnell C, Pachter L, Salzberg SL. TopHat: discovering splice junctions with RNA-Seq. Bioinformatics. 2009; 25: 1105-11. doi: 10.1093/bioinformatics/btp120.
13. Langmead B, Trapnell C, Pop M, Salzberg SL. Ultrafast and memory-efficient alignment of short DNA sequences to the human genome. Genome Biol. 2009; 10: R25. doi: 10.1186/gb-2009-10-3-r25.
14. Anders S, Pyl PT, Huber W. HTSeq--a Python framework to work with high-throughput sequencing data. Bioinformatics. 2015; 31: 166-9. doi: 10.1093/bioinformatics/btu638.
15. Robinson MD, McCarthy DJ, Smyth GK. edgeR: a Bioconductor package for differential expression analysis of digital gene expression data. Bioinformatics. 2010; 26: 139-40. doi: 10.1093/bioinformatics/btp616.
16. Zhu Y, Qiu P, Ji Y. TCGA-assembler: open-source software for retrieving and processing TCGA data. Nat Methods. 2014; 11: 599-600. doi: 10.1038/nmeth.2956.
17. Zhu Y, Xu Y, Helseth DL, Jr., Gulukota K, Yang S, Pesce LL, Mitra R, Muller P, Sengupta S, Guo W, Silverstein JC, Foster I, Parsad N, et al. Zodiac: A Comprehensive Depiction of Genetic Interactions in Cancer by Integrating TCGA Data. J Natl Cancer Inst. 2015; 107. doi: 10.1093/jnci/djv129.
18. Lowe R, Rakyan VK. Marmal-aid--a database for Infinium HumanMethylation450. BMC Bioinformatics. 2013; 14: 359. doi: 10.1186/1471-2105-14-359.
19. Morris TJ, Butcher LM, Feber A, Teschendorff AE, Chakravarthy AR, Wojdacz TK, Beck S. ChAMP: 450k Chip Analysis Methylation Pipeline. Bioinformatics. 2014; 30: 428-30. doi: 10.1093/bioinformatics/btt684.

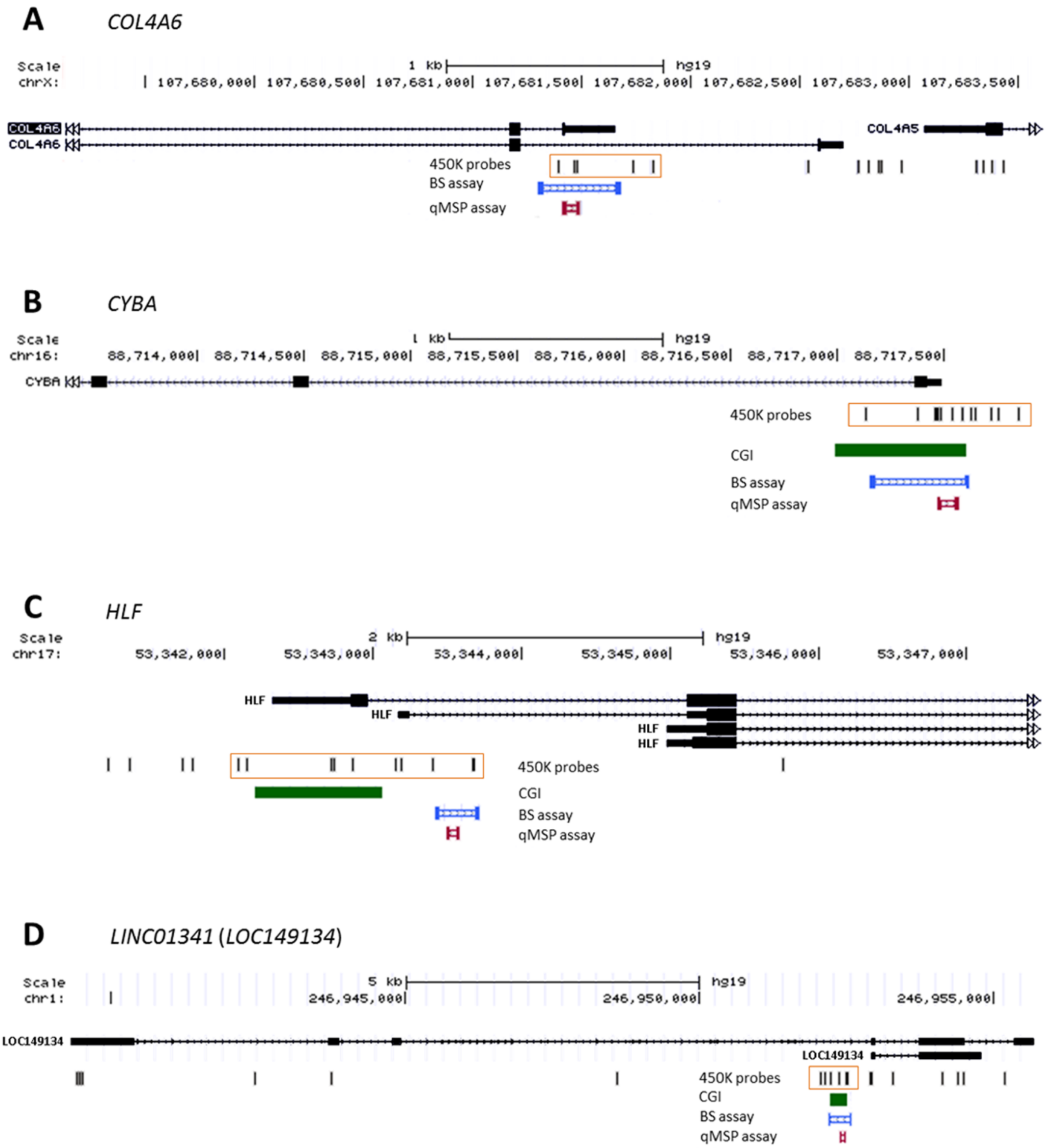
A
COL4A6
Scale
chrX:
1 kb
hg19
107,680,000| 107,680,500| 107,681,000| 107,681,500| 107,682,000| 107,682,500| 107,683,000| 107,683,500|
COL4A6
COL4A6
COL4A5
450K probes
BS assay
qMSP assay
B
CYBA
Scale
chr16:
1 kb
hg19
88,714,000| 88,714,500| 88,715,000| 88,715,500| 88,716,000| 88,716,500| 88,717,000| 88,717,500|
CYBA
450K probes
CGI
BS assay
qMSP assay
C
HLF
Scale
chr17:
2 kb
hg19
53,342,000| 53,343,000| 53,344,000| 53,345,000| 53,346,000| 53,347,000|
HLF
HLF
HLF
HLF
450K probes
CGI
BS assay
qMSP assay
D
LINC01341 (LOC149134)
Scale
chr1:
5 kb
hg19
246,945,000| 246,950,000| 246,955,000|
LOC149134
LOC149134
450K probes
CGI
BS assay
qMSP assay

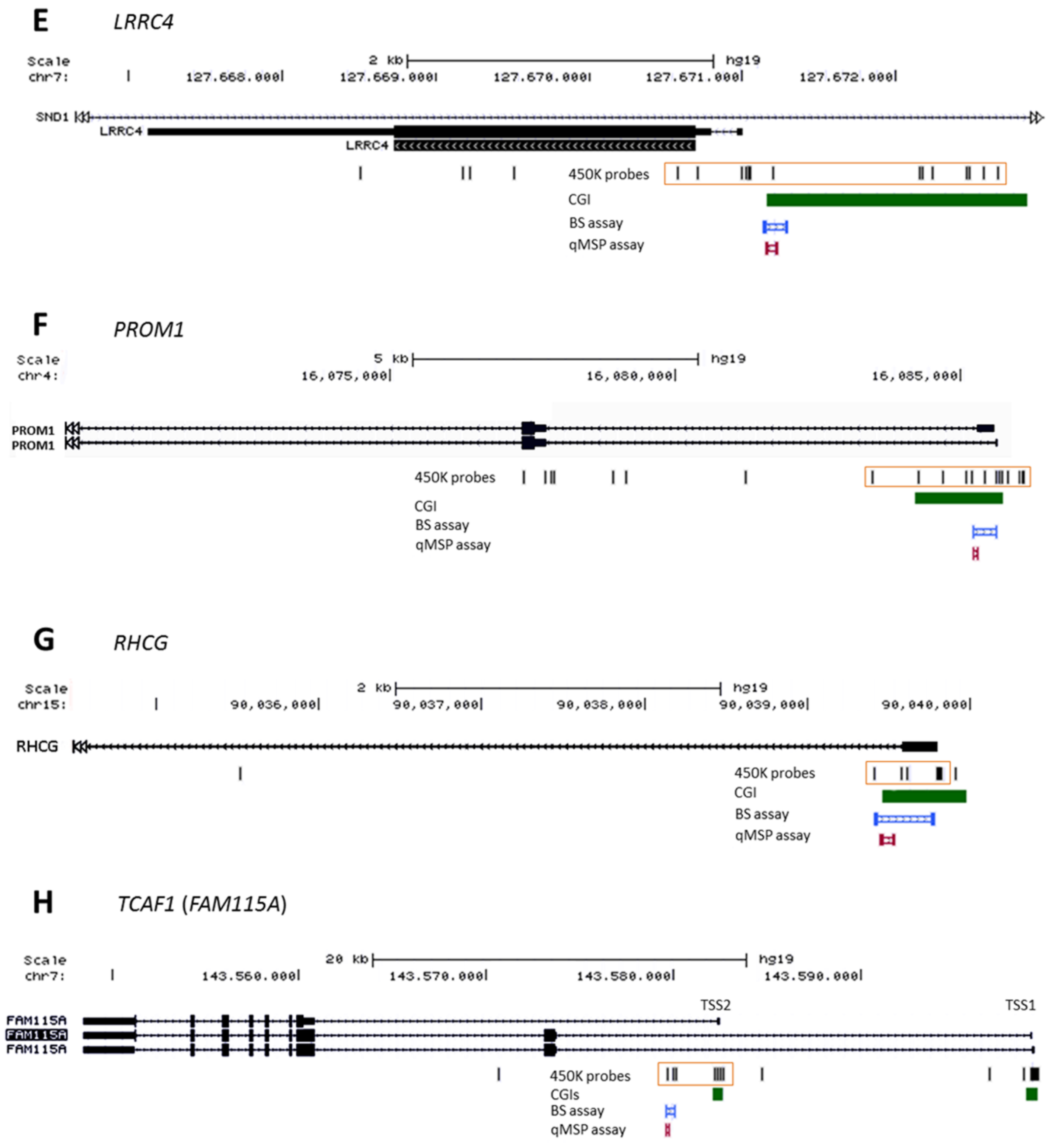

Fig. S1: Gene structure of the 8 candidate markers. Overview including the promoter region, associated candidate methylation marker loci, and location of BS/qMSP assays. Modified from UCSC Genome Browser (RefSeq/GENCODE, hg 19, screen shot). **A:** *COL4A6*. **B:** *CYBA*. **C:** *HLF*. **D:** *LINC01341* (*LOC149134*). **E:** *LRRC4*. **F:** *PROM1*. **G:** *RHCG*. **H:** *TCAF1* (*FAM115A*). Black vertical bars: 450K probe sites. Orange box:

Significantly hypermethylated DMCs according to 450K analysis. Green bars: CGIs. Blue bars: Location of BS assays. Red bars: Location of qMSP assays.

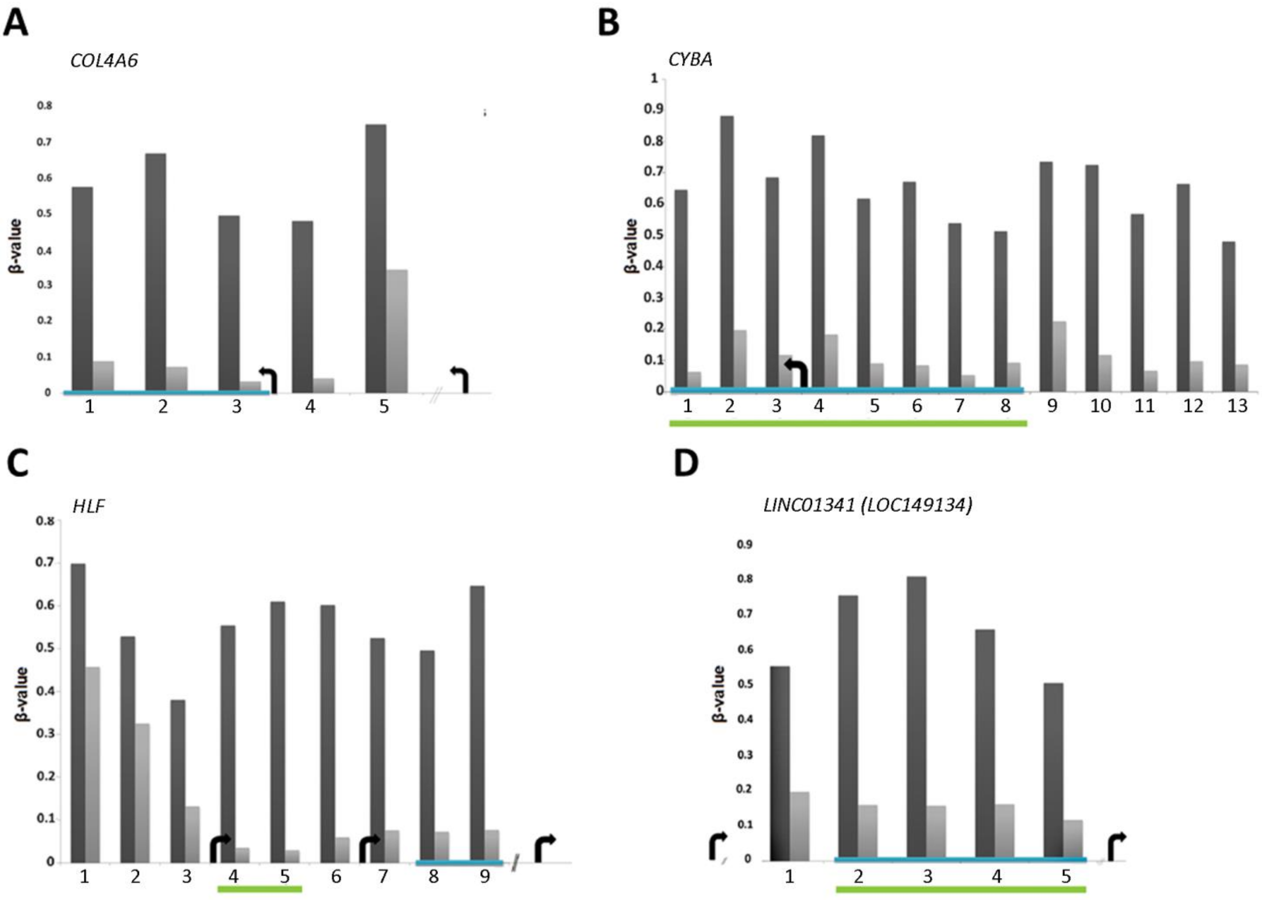

A
COL4A6
β-value
B
CYBA
β-value
C
HLF
β-value
D
LINC01341 (LOC149134)
β-value

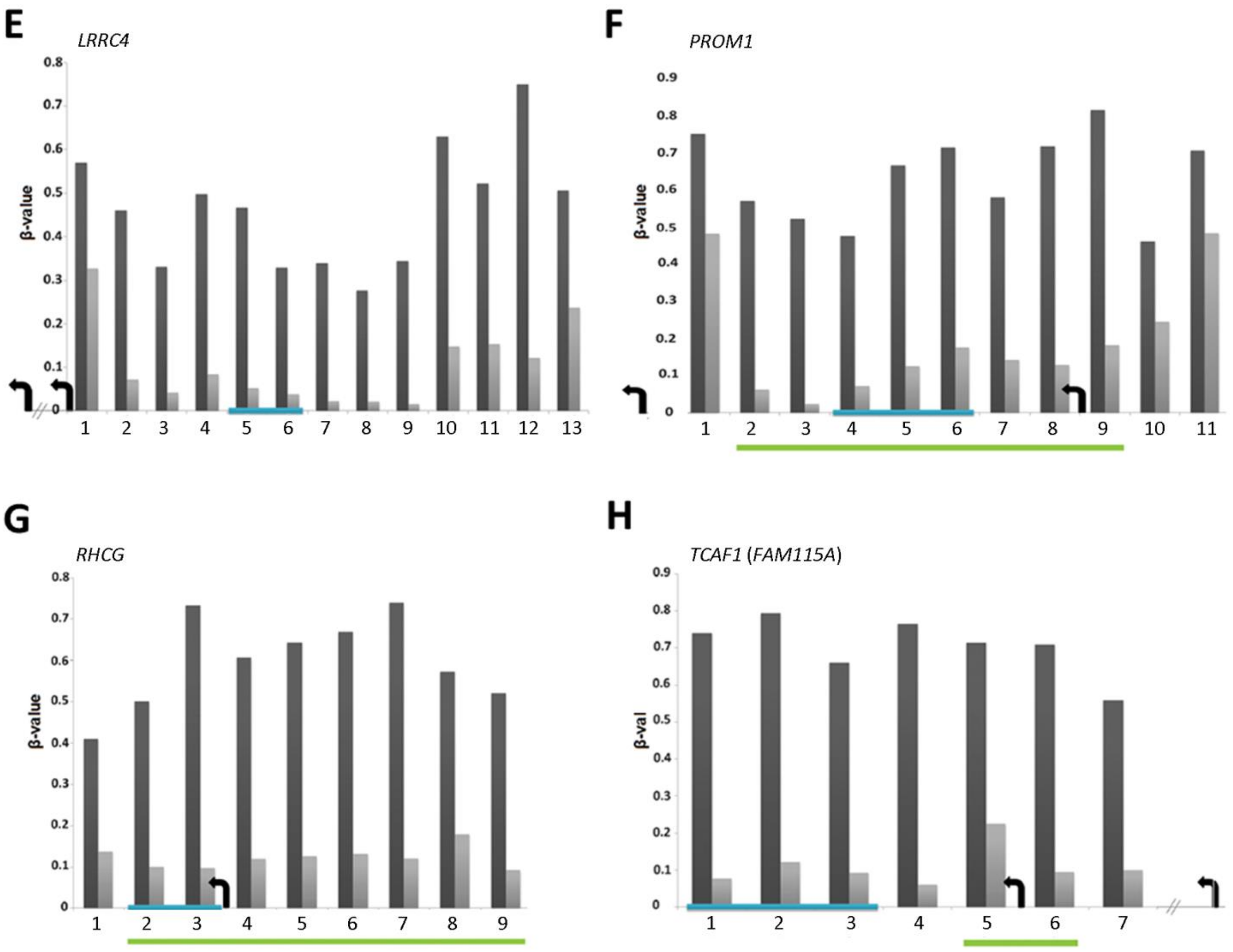

Fig. S2: Candidate marker methylation levels. Mean 450K β-values in 21 T (dark grey) and 21 NM (light grey) samples for DMCs (mean $\Delta\beta \geq |0.2|$ and adj. $P<0.05$) associated to the 8 candidate methylation loci. **A:** *COL4A6*. **B:** *CYBA*. **C:** *HLF*. **D:** *LINC01341* (*LOC149134*). **E:** *LRRC4*. **F:** *PROM1*. **G:** *RHCG*. **H:** *TCAF1* (*FAM115A*). Blue bars: CpG sites investigated by BS. Green bars: CGIs. Arrows: TSS, indicating transcriptional direction. CpG site numbers refer to CpG sites in fig. S3 and table S3.

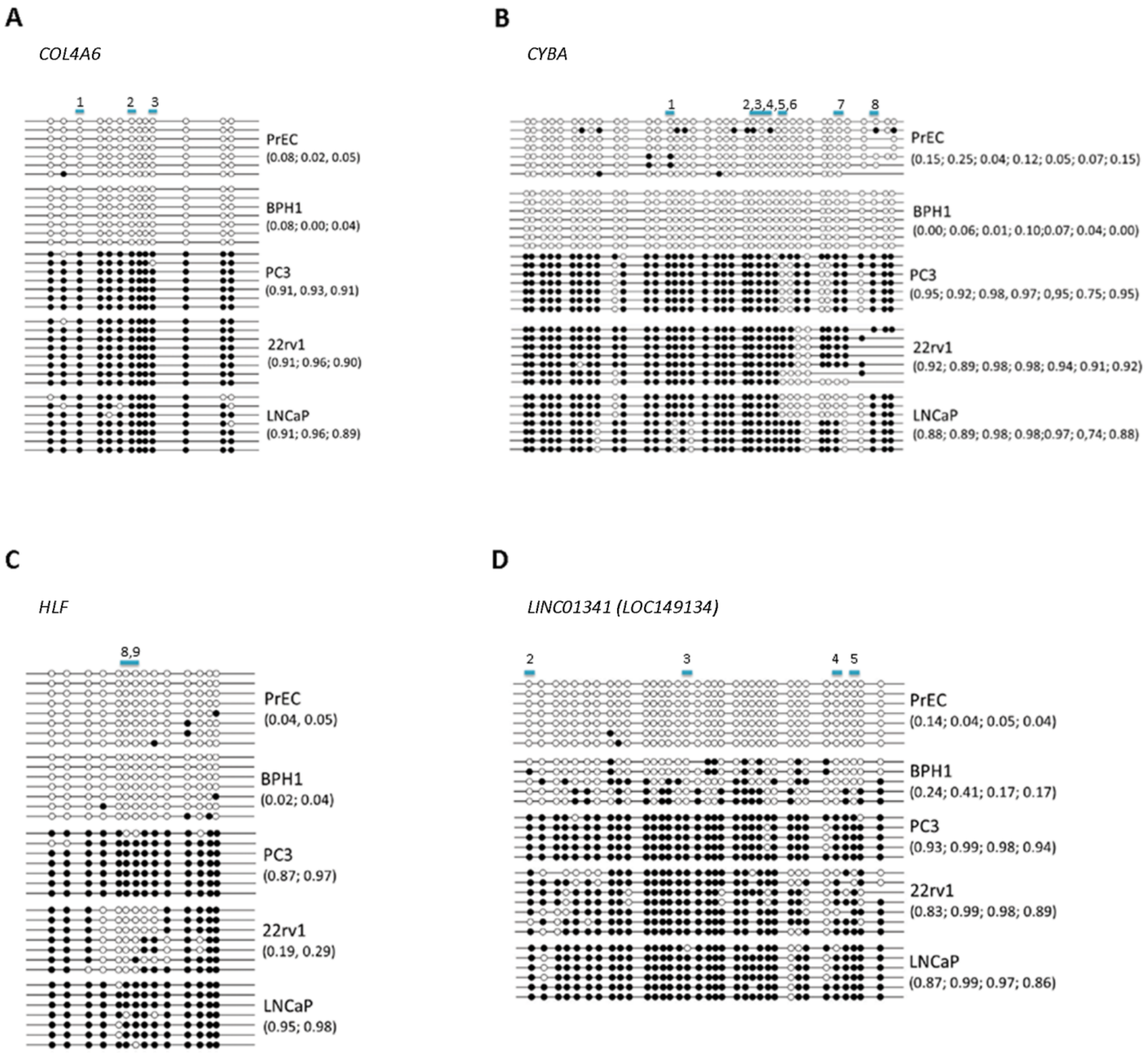
A
COL4A6
1
2
3
PrEC
(0.08; 0.02, 0.05)
BPH1
(0.08; 0.00; 0.04)
PC3
(0.91, 0.93, 0.91)
22rv1
(0.91; 0.96; 0.90)
LNCaP
(0.91; 0.96; 0.89)
B
CYBA
1
2,3,4,5,6
7
8
PrEC
(0.15; 0.25; 0.04; 0.12; 0.05; 0.07; 0.15)
BPH1
(0.00; 0.06; 0.01; 0.10;0.07; 0.04; 0.00)
PC3
(0.95; 0.92; 0.98; 0.97; 0,95; 0.75; 0.95)
22rv1
(0.92; 0.89; 0.98; 0.98; 0.94; 0.91; 0.92)
LNCaP
(0.88; 0.89; 0.98; 0.98;0.97; 0,74; 0.88)
C
HLF
8,9
PrEC
(0.04, 0.05)
BPH1
(0.02; 0.04)
PC3
(0.87; 0.97)
22rv1
(0.19, 0.29)
LNCaP
(0.95; 0.98)
D
LINC01341 (LOC149134)
2
3
4
5
PrEC
(0.14; 0.04; 0.05; 0.04)
BPH1
(0.24; 0.41; 0.17; 0.17)
PC3
(0.93; 0.99; 0.98; 0.94)
22rv1
(0.83; 0.99; 0.98; 0.89)
LNCaP
(0.87; 0.99; 0.97; 0.86)

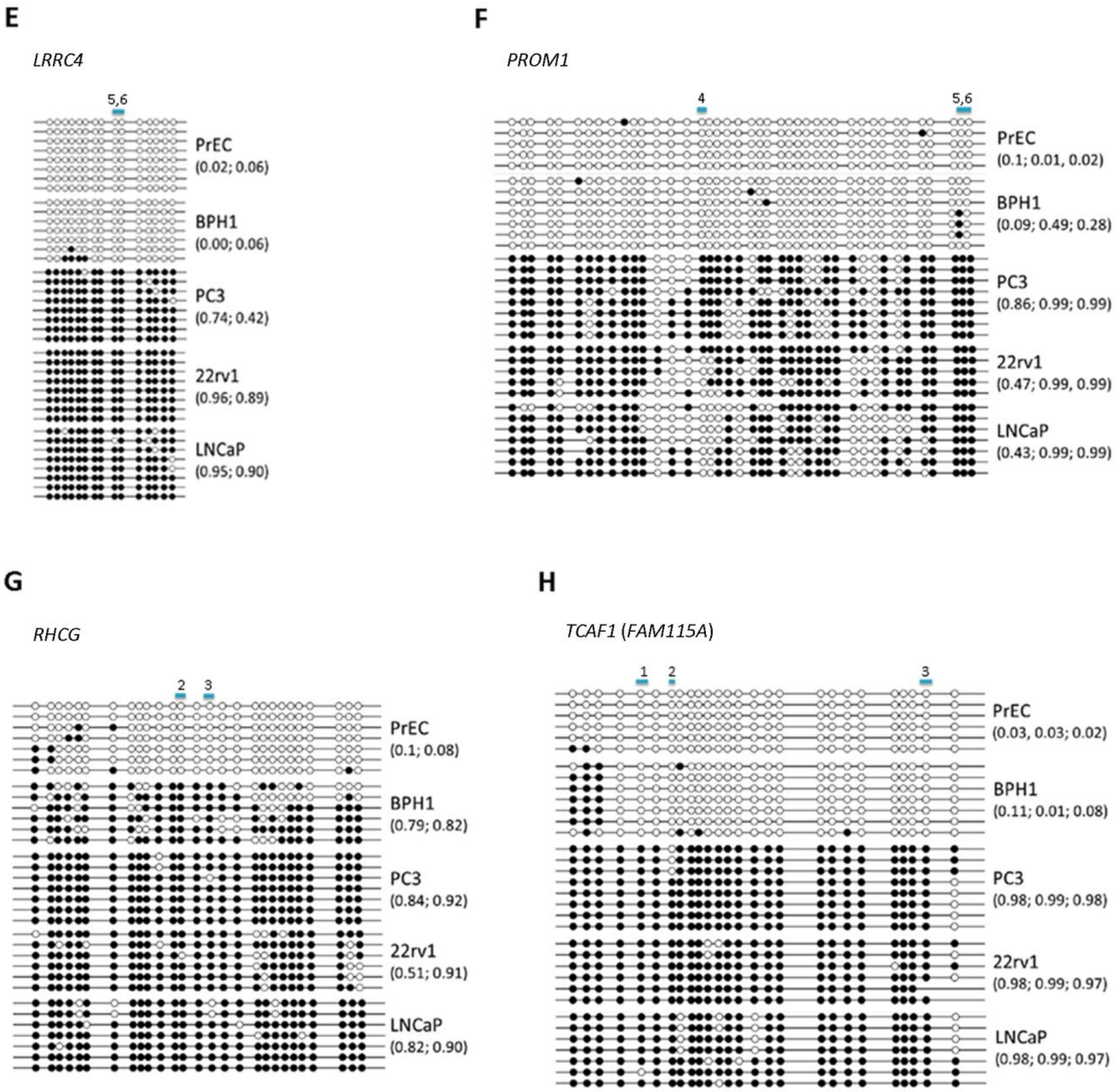

Fig. S3: Validation of 450K results by BS of cell line DNA. PrEC, BPH1: NM prostate epithelial cell lines; PC3, LNCaP, 22rv1: Malignant prostate cell lines. Filled and open circles represent methylated and unmethylated CpG sites, respectively. Each line represents one clone. Blue bars mark CpG sites investigated by the 450K array, and β-values (450K) for these sites are listed in brackets next to the bisulfite sequencing results for the respective cell lines. CpG site numbers refer to CpG sites in fig. S2 and table S3. **A:** *COL4A6*. **B***:* *CYBA*. **C:** *HLF*. **D:** *LINC01341* (*LOC149134*). **E:** *LRRC4.* **F***:* *PROM1*. **G:** *RHCG*. **H:** *TCAF1* (*FAM115A*).

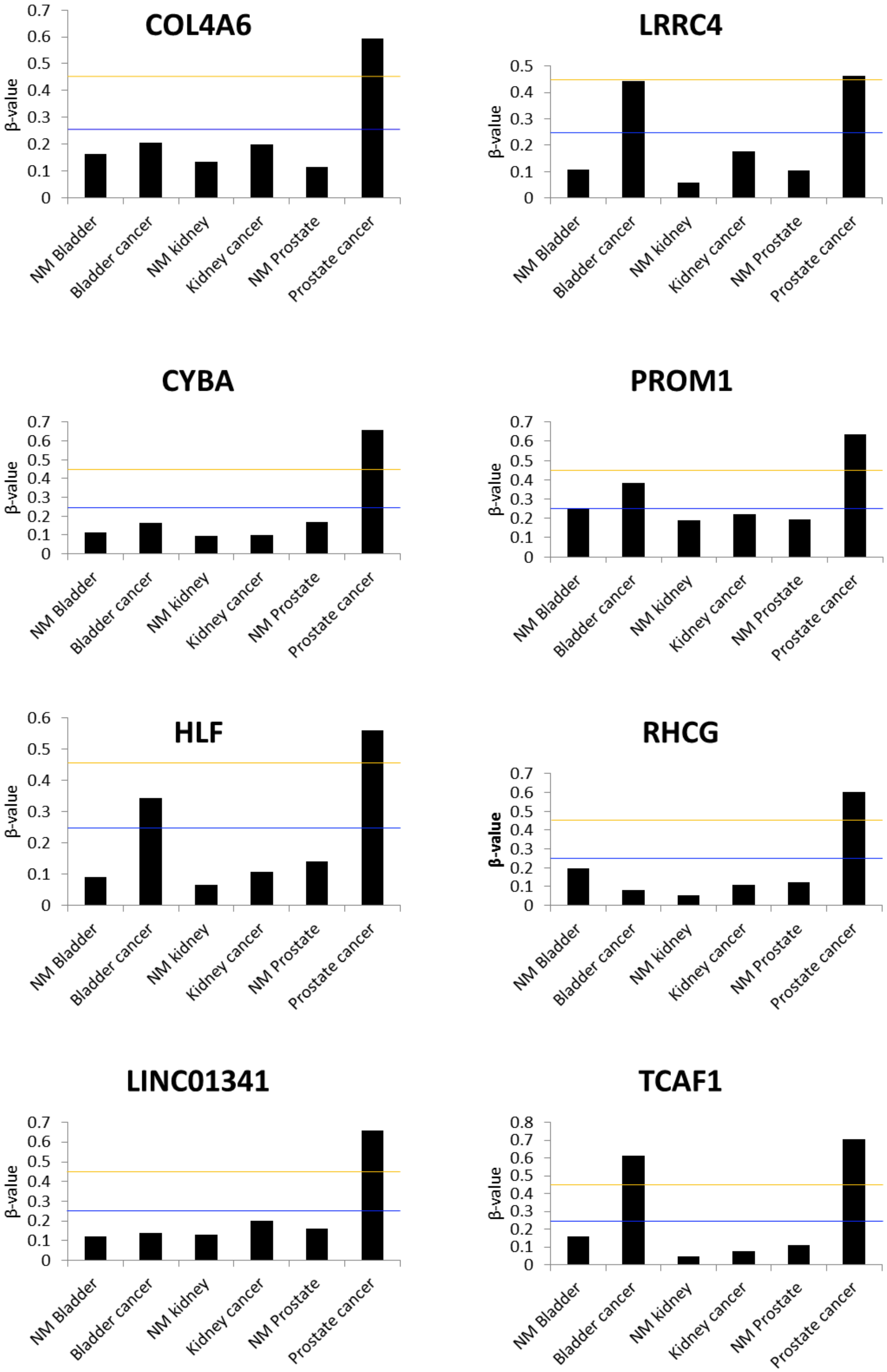
COL4A6
LRRC4
CYBA
PROM1
HLF
RHCG
LINC01341
TCAF1
β-value
NM Bladder
Bladder cancer
NM kidney
Kidney cancer
NM Prostate
Prostate cancer

Fig. S4: Mean β-value of all CpG sites identified as being hypermethylated in prostate cancer (supplementary table S3) for each candidate in malignant and non-malignant bladder and kidney tissue specimens from the Marmal-aid database, in addition to prostate specimens from the present analysis. The lines mark arbitrary thresholds for hypomethylation (blue, mean $\beta<0.25$) and hypermethylation (orange, mean $\beta>0.45$). Bladder cancer: N=85. NM Bladder: N=10. Kidney cancer: N=244. NM kidney N=136. Prostate cancer: N=21. NM prostate: N=21.

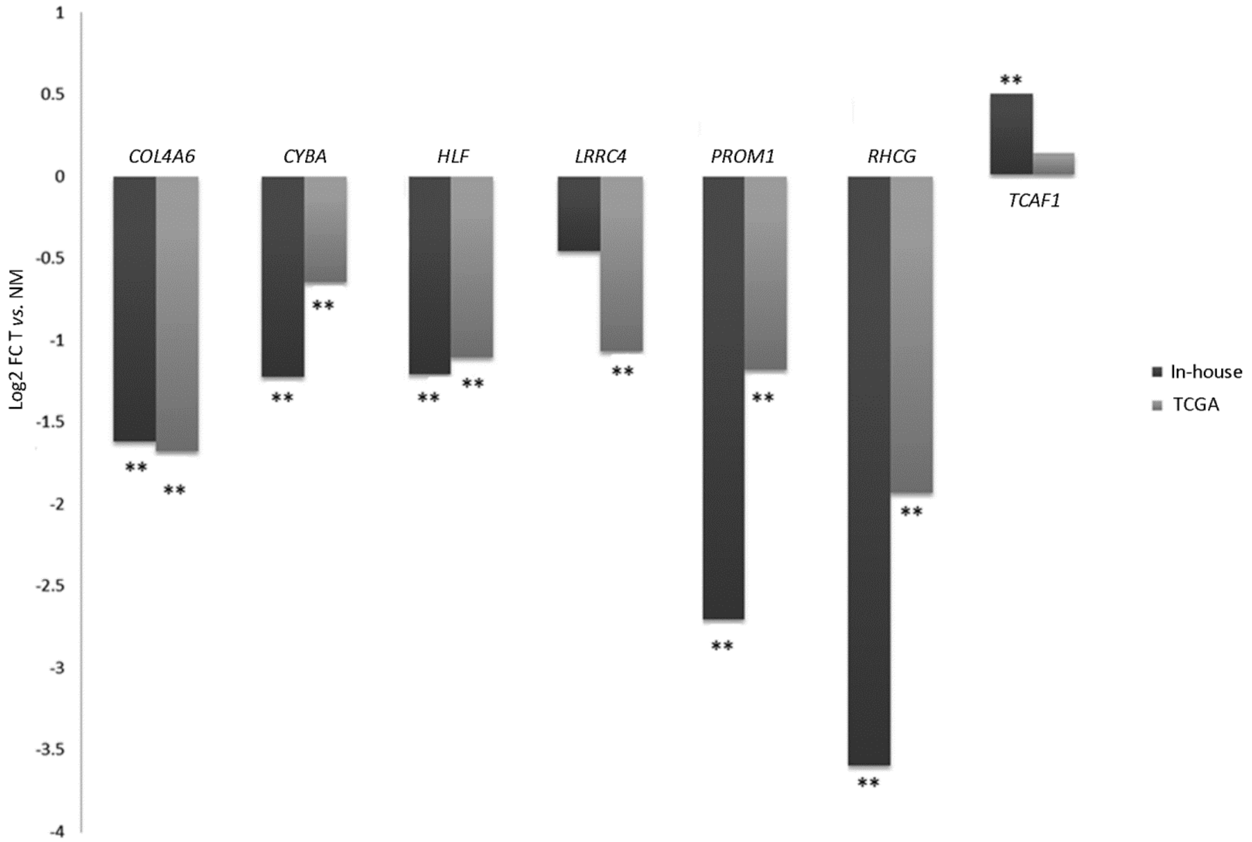

Fig. S5: Differential candidate gene expression in T *vs.* NM prostate samples. TCGA and in-house RNA-seq data for 7 of the 8 candidate genes. *LINC01341* was not annotated in either data set. Log2 fold-change (FC) T *vs.* NM samples (EdgeR analysis). (**) adj. $P<0.01$. TCGA: T, $N=297$; NM, $N=34$. In-house: T, $N=14$; NM, $N=12$.

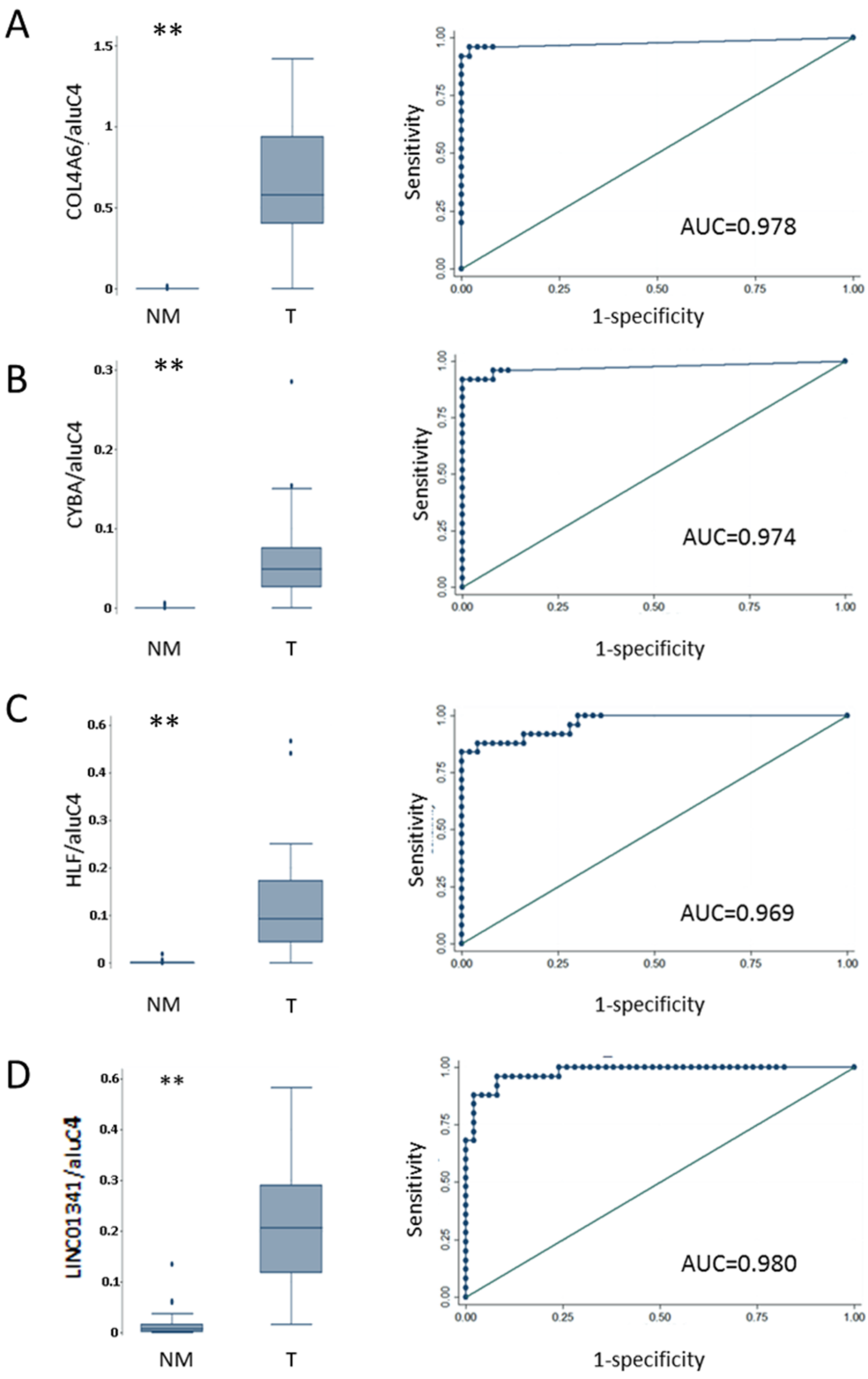
A
**
COL4A6/aluC4
1.5
1
0.5
0
NM
T
Sensitivity
1.00
0.75
0.50
0.25
0.00
AUC=0.978
1-specificity
B
**
CYBA/aluC4
0.3
0.2
0.1
0
NM
T
AUC=0.974
1-specificity
C
**
HLF/aluC4
0.6
0.4
0.3
0.2
0.1
0
NM
T
AUC=0.969
1-specificity
D
**
LINC01341/aluC4
0.6
0.4
0.3
0.2
0.1
0
NM
T
AUC=0.980
1-specificity

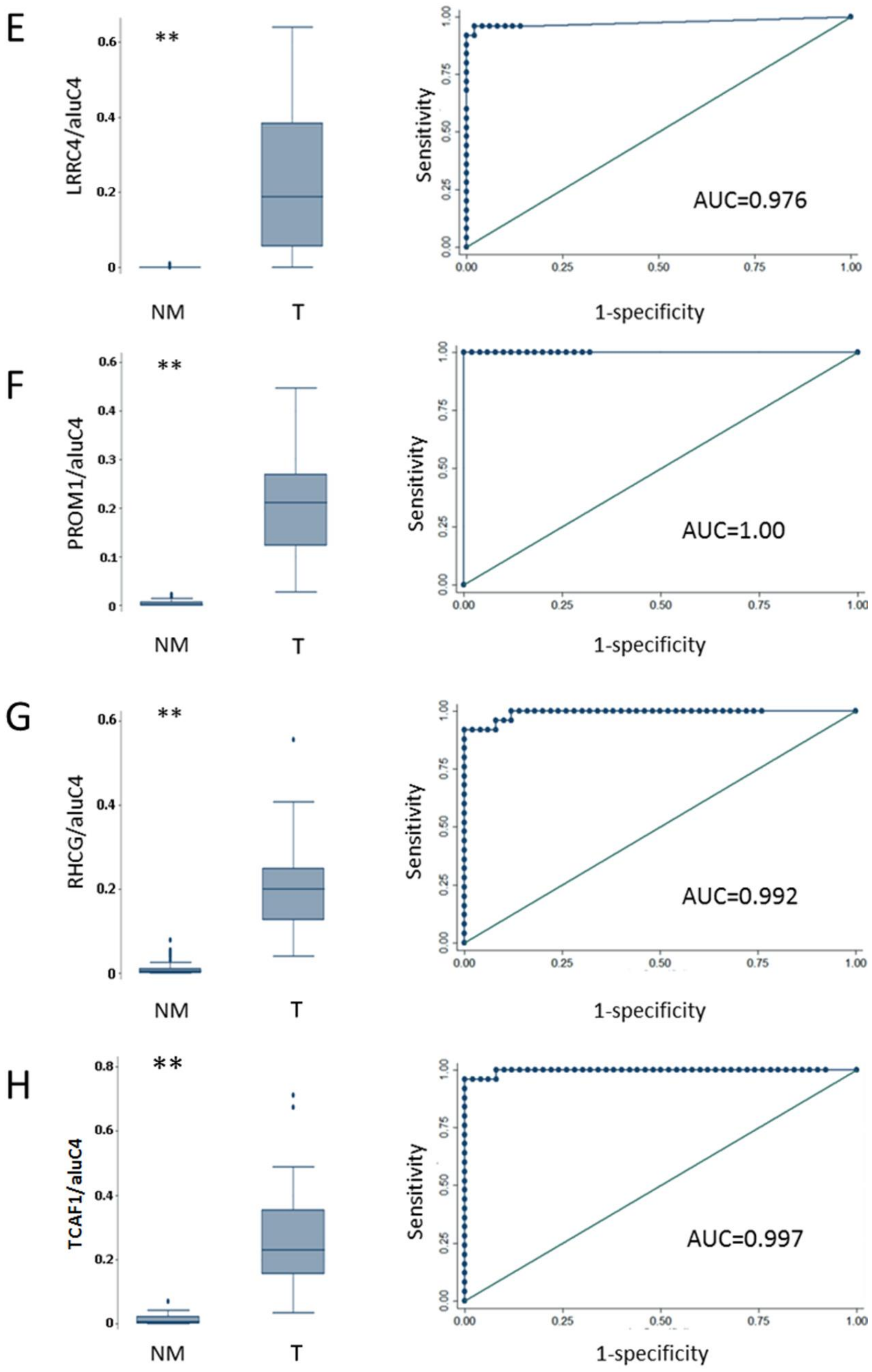
E
**
LRRC4/aluC4
NM
T
Sensitivity
1-specificity
AUC=0.976
F
**
PROM1/aluC4
NM
T
Sensitivity
1-specificity
AUC=1.00
G
**
RHCG/aluC4
NM
T
Sensitivity
1-specificity
AUC=0.992
H
**
TCAF1/aluC4
NM
T
Sensitivity
1-specificity
AUC=0.997

Fig. S6: Diagnostic potential of candidate methylation markers in DNBs. Receiver operating characteristics (ROC) analysis of NM samples (AN and N, *N*=50) vs. T samples (*N*=25). Left: Box plots of methylation levels of NM and T biopsy specimens. (**) *P*<0.001, rank-sum test. Right: ROC curves of data displayed in box plots. **A:** *COL4A6*. **B:** *CYBA*. **C:** *HLF*. **D:** *LINC01341* (*LOC149134*). **E:** *LRRC4*. **F:** *PROM1*. **G:** *RHCG*. **H:** *TCAF1* (*FAM115A*).

Cohort 1

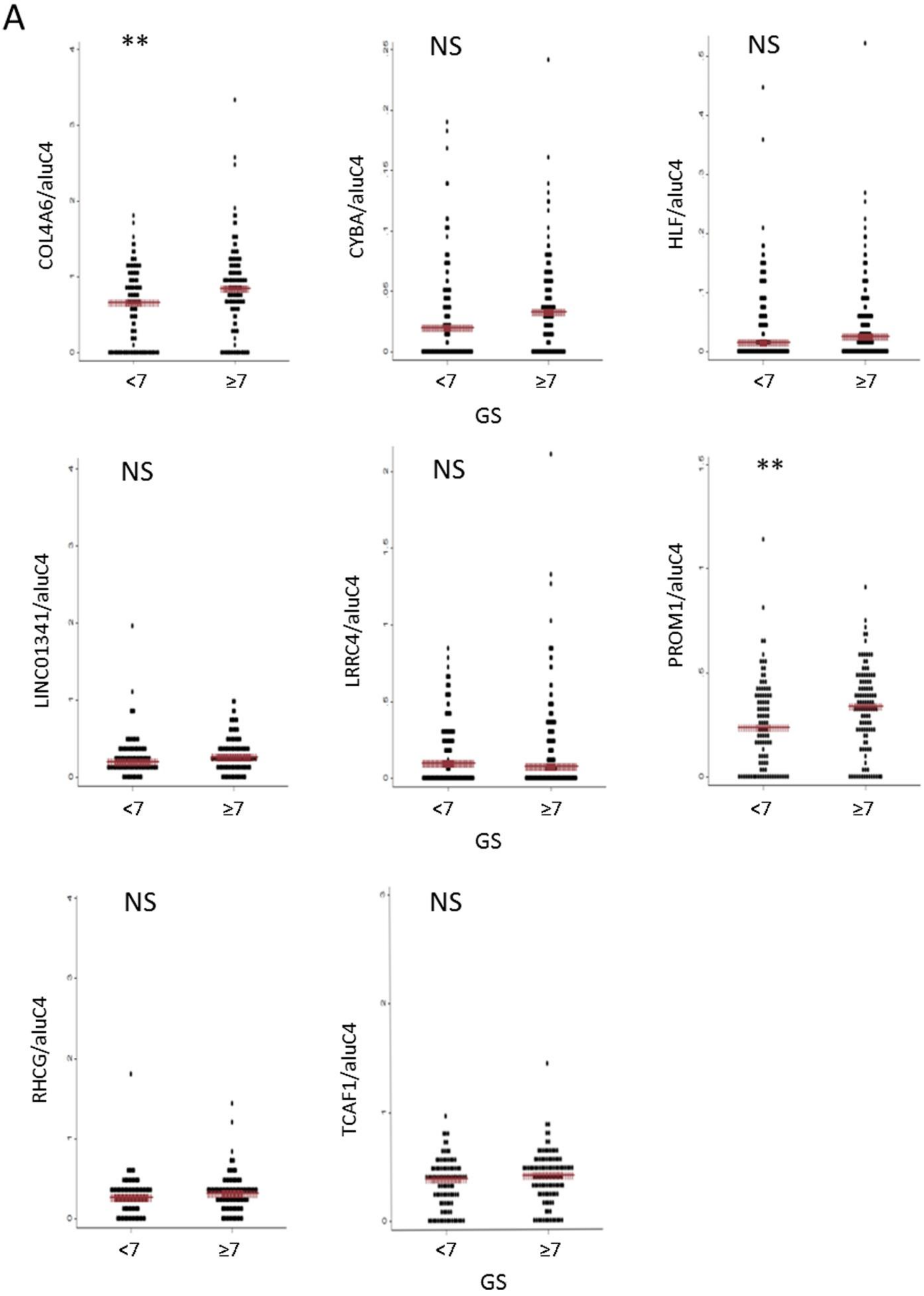

A
**
COL4A6/aluC4
NS
CYBA/aluC4
NS
HLF/aluC4
NS
LINC01341/aluC4
NS
LRRC4/aluC4
**
PROM1/aluC4
NS
RHCG/aluC4
NS
TCAF1/aluC4
<7
≥7
GS

Cohort 2

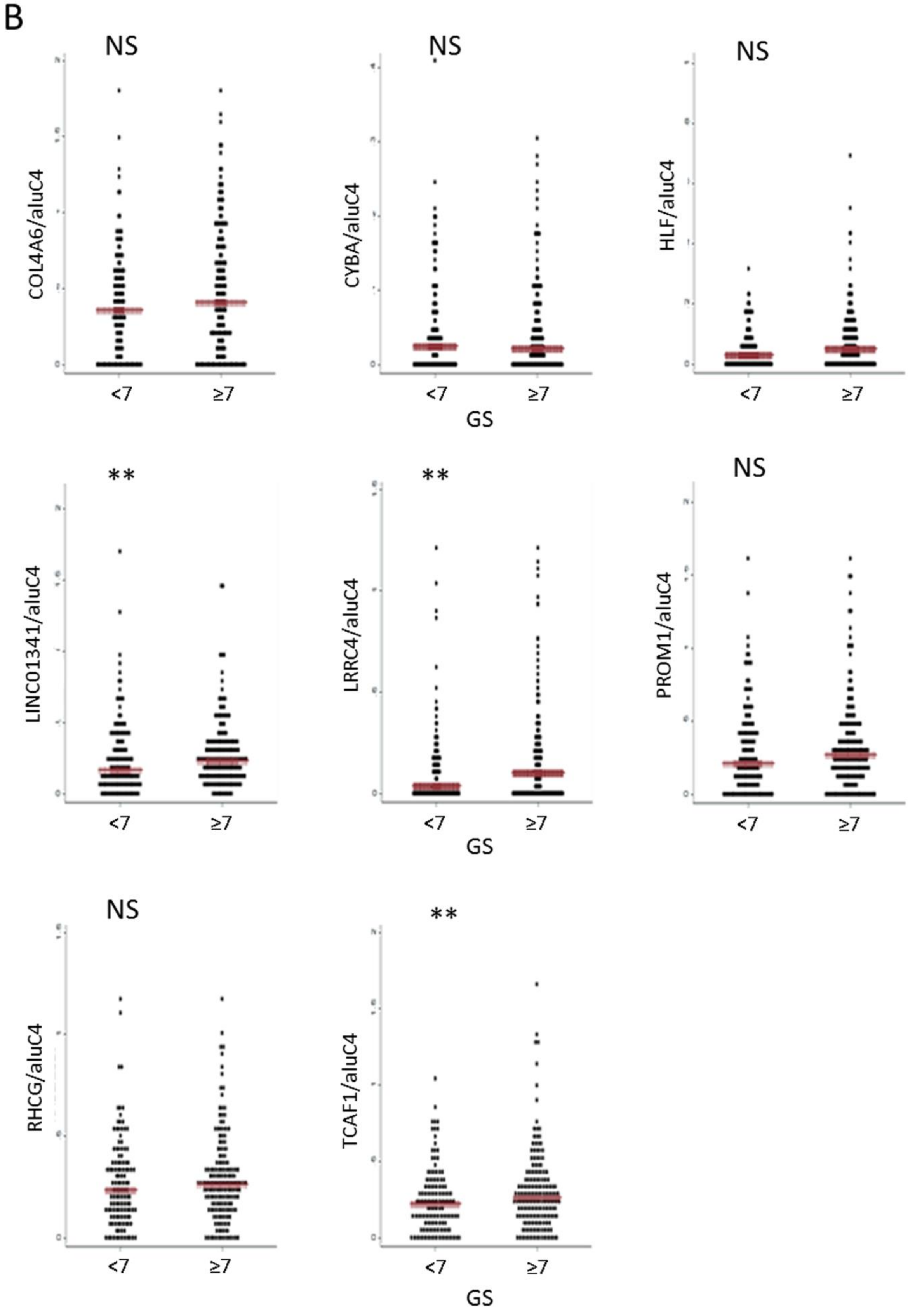
B
NS
COL4A6/aluC4
<7
≥7
NS
CYBA/aluC4
<7
≥7
GS
NS
HLF/aluC4
<7
≥7
**
LINC01341/aluC4
<7
≥7
**
LRRC4/aluC4
<7
≥7
GS
NS
PROM1/aluC4
<7
≥7
NS
RHCG/aluC4
<7
≥7
**
TCAF1/aluC4
<7
≥7
GS

Cohort 1

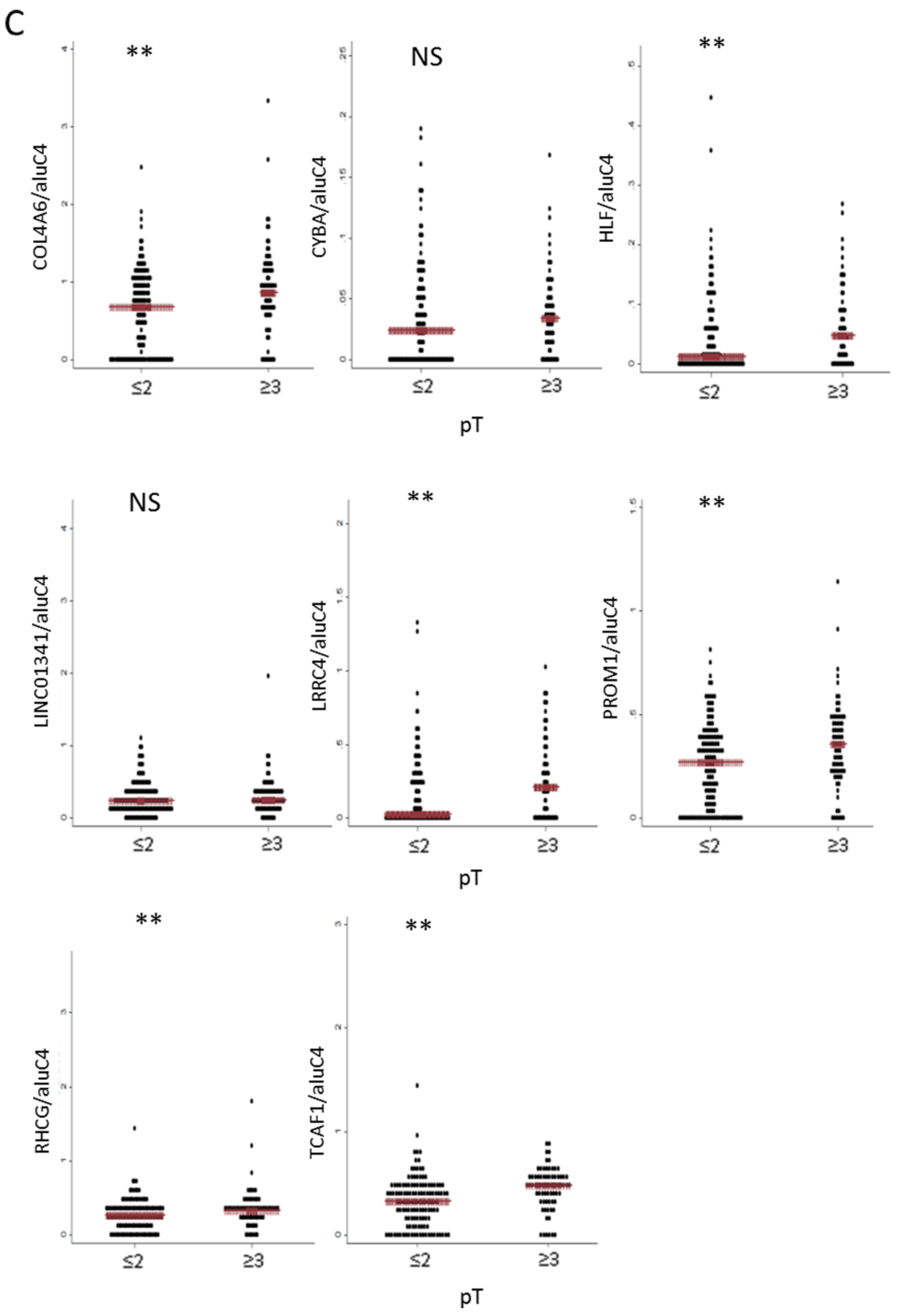
C
**
COL4A6/aluC4
≤2
≥3
NS
CYBA/aluC4
≤2
≥3
**
HLF/aluC4
≤2
≥3
pT
NS
LINC01341/aluC4
≤2
≥3
**
LRRC4/aluC4
≤2
≥3
**
PROM1/aluC4
≤2
≥3
pT
**
RHCG/aluC4
≤2
≥3
**
TCAF1/aluC4
≤2
≥3
pT

Cohort 2

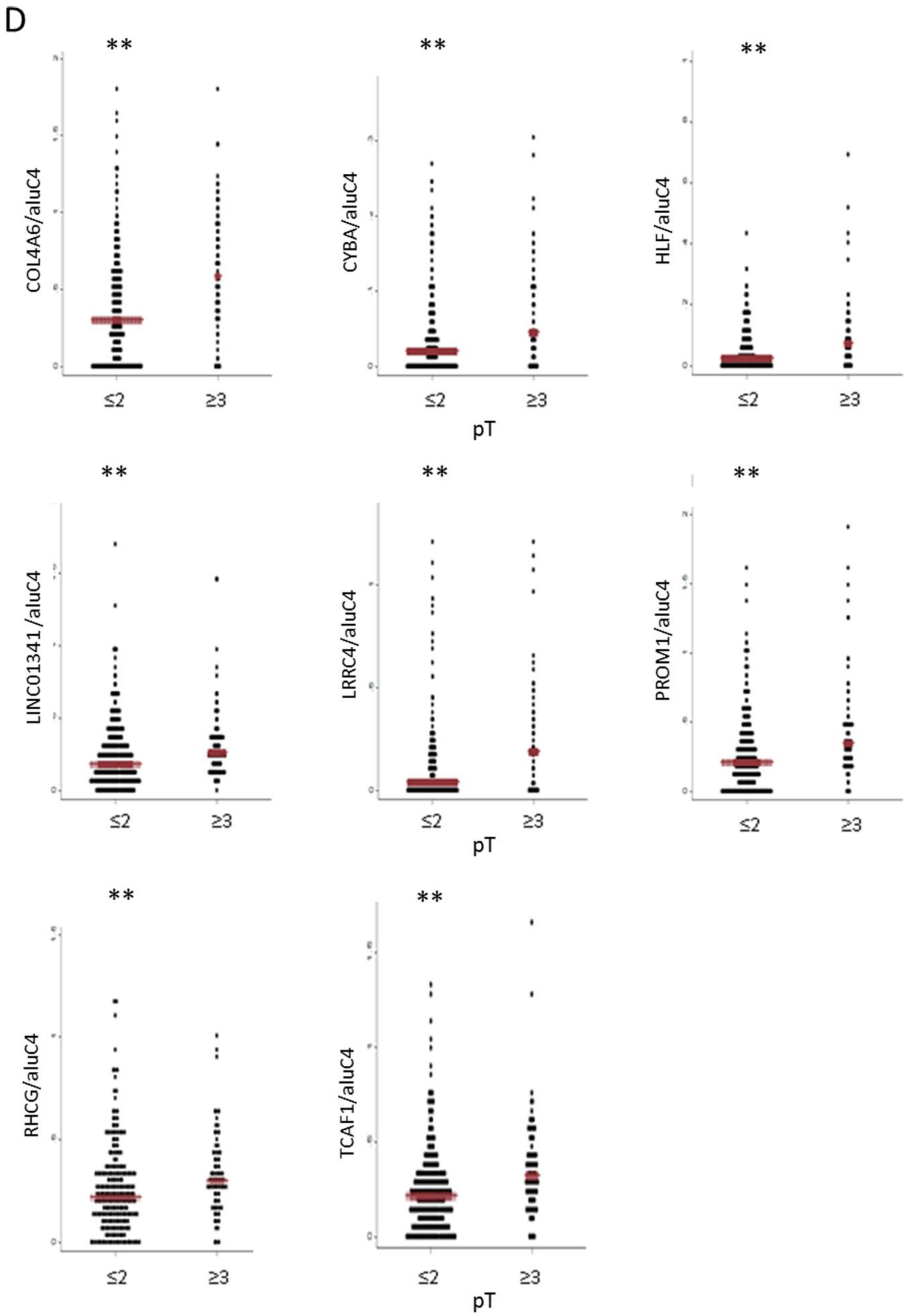

D
COL4A6/aluC4
CYBA/aluC4
HLF/aluC4
LINC01341/aluC4
LRRC4/aluC4
PROM1/aluC4
RHCG/aluC4
TCAF1/aluC4
**
≤2
≥3
pT

Cohort 1

E

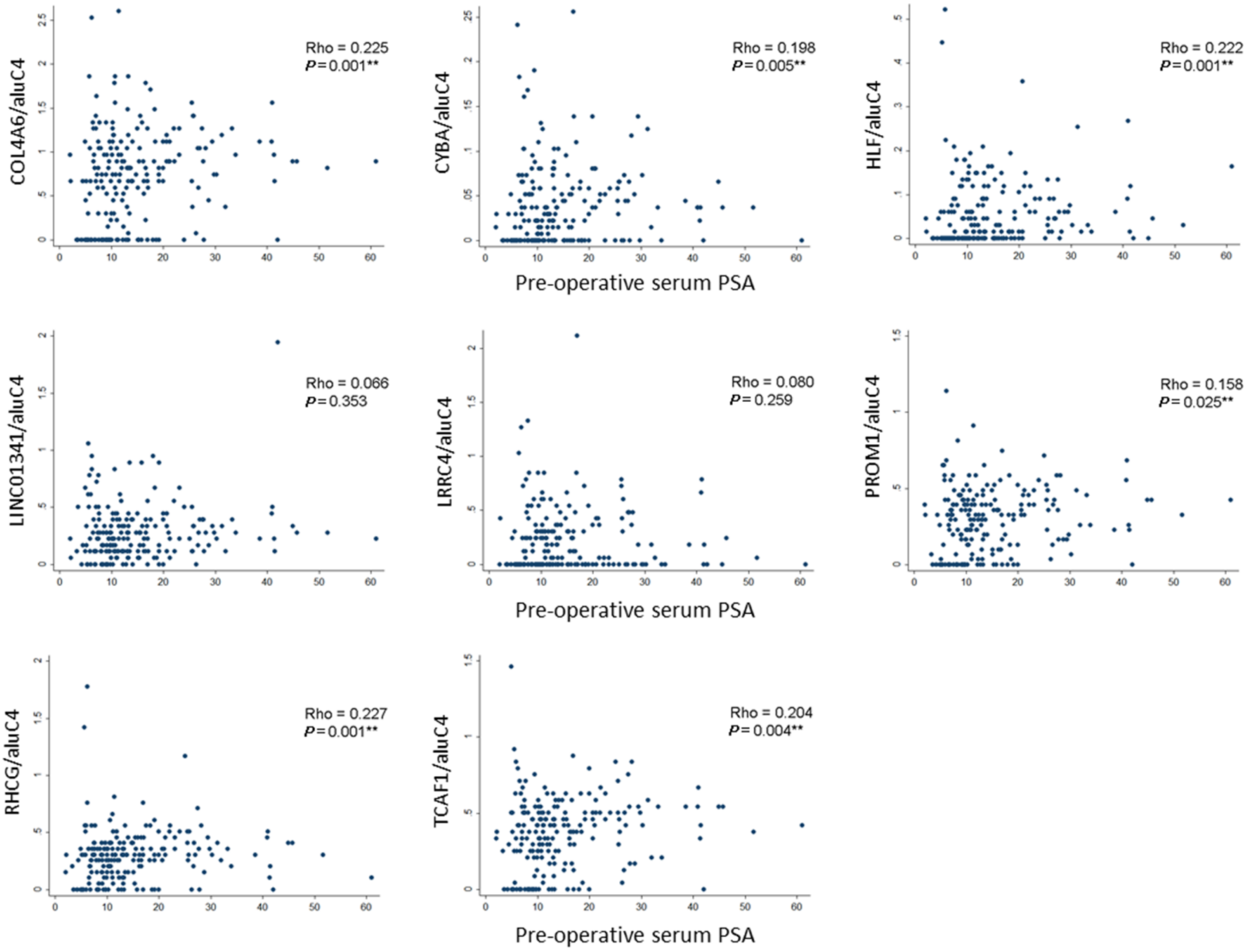

COL4A6/aluC4
Rho = 0.225
P=0.001**
CYBA/aluC4
Rho = 0.198
P=0.005**
HLF/aluC4
Rho = 0.222
P=0.001**
Pre-operative serum PSA
LINC01341/aluC4
Rho = 0.066
P=0.353
LRRC4/aluC4
Rho = 0.080
P=0.259
PROM1/aluC4
Rho = 0.158
P=0.025**
Pre-operative serum PSA
RHCG/aluC4
Rho = 0.227
P=0.001**
TCAF1/aluC4
Rho = 0.204
P=0.004**
Pre-operative serum PSA

Cohort 2

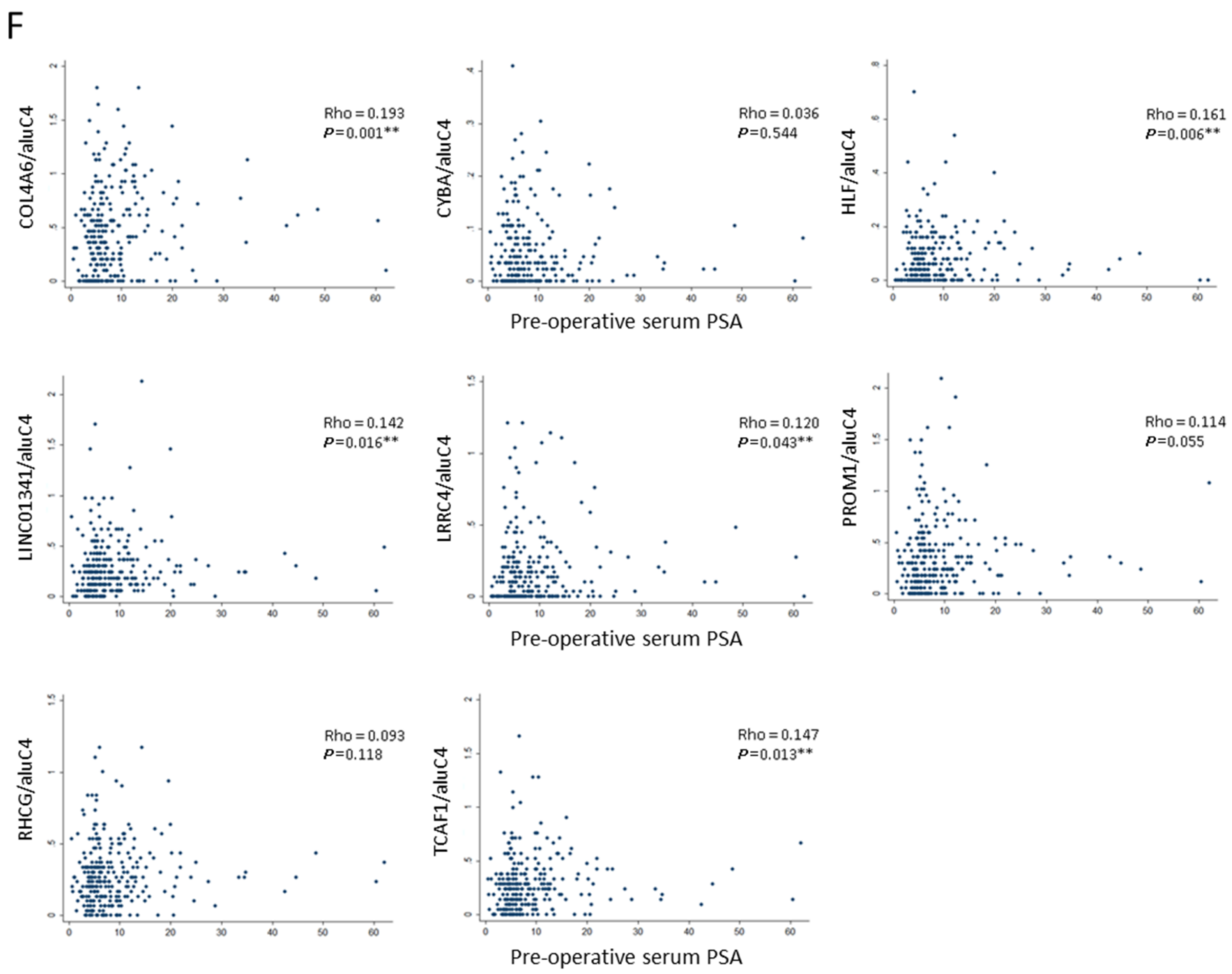

Fig. S7: Candidate marker methylation levels *vs.* clinicopathological variables. A, B: Methylation levels of individual candidate markers in cohort 1 (**A**) and 2 (**B**) stratified by Gleason score (<7 *vs.* ≥7). Red bar: Median. *P*-values from rank-sum test. **C, D:** Methylation levels of individual candidate markers in cohort 1 (**C**) and 2 (**D**) stratified by pathological tumor stage (≤2 *vs.* ≥3). Red bar: Median. *P*-values from rank-sum test. **E, F:** Dotplot of association between methylation levels of individual candidate markers in cohort 1 (**E**) and 2 (**F**) and pre-operative PSA levels. Spearman's Rho and *P*-values are given for each gene.

Dots: Relative quantity of methylated DNA/relative quantity of aluC4. (**) *P*<0.05. NS: Not significant.

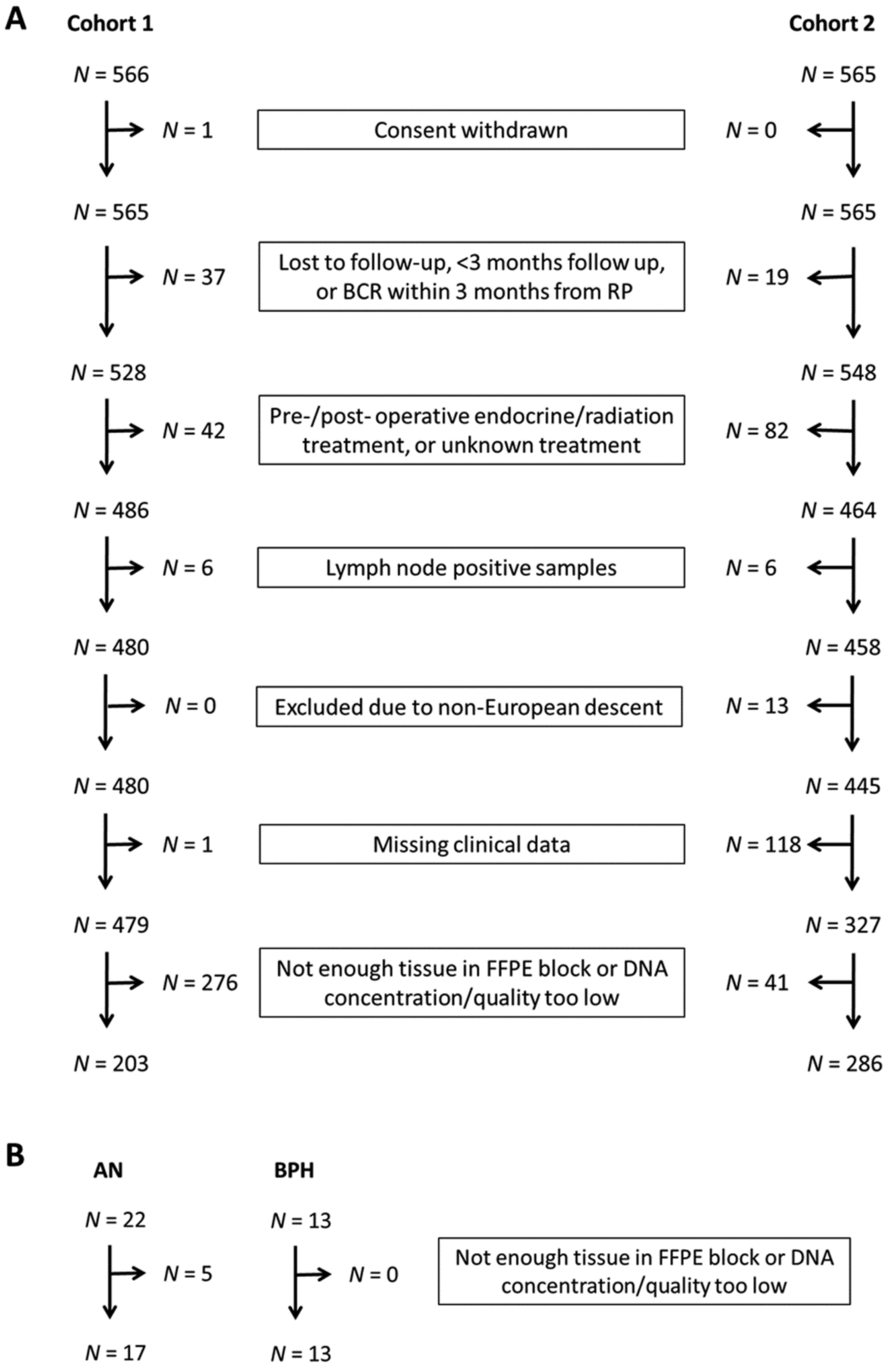

Fig. S8: Sample inclusion/exclusion process for qMSP analysis according to REMARK criteria. A: T samples in cohort 1 and 2. **B:** NM samples in cohort 1.
