# Supplementary material for: RHCG and TCAF1 promoter hypermethylation predicts biochemical recurrence in prostate cancer patients treated by radical prostatectomy
Source: Oncotarget. 2016 Dec 30;8(4):5774–88. doi: 10.18632/oncotarget.14391 (PMC5351588; doi:10.18632/oncotarget.14391)
Supplement: Supplementary file 2 [file oncotarget-08-5774-s002.docx]

|  | **T** | **AN** | **N** |
| --- | --- | --- | --- |
| *N* | 21 | 12 | 9 |
| Age at RP, median (range) | 60.1 (49 -72) | 61.6 (47 - 69) | 65.1 (58 - 80) |
| **Pathological Gleason score** |  |  |  |
| <7, *N* (%) | 2 (9.5 %) |  |  |
| =7, *N* (%) | 13 (61.9 %) |  |  |
| >7, *N* (%) | 5 (23.8 %) |  |  |
| Unknown, *N* (%) | 1 (4.8 %) |  |  |
| **Pathological T-stage (n)** |  |  |  |
| ≤pT2c, *N* (%) | 11 (52.4 %) |  |  |
| ≥pT3a, *N* (%) | 9 (42.9 %) |  |  |
| Unknown, *N* (%) | 1 (4.8 %) |  |  |
| **Pre-operative PSA** |  |  |  |
| PSA ng/ml, median (range) | 16.0 (5.0 - 37.0) |  |  |
| Unknown, *N* (%) | 1 (4.8 %) |  |  |
| **Surgical margin status** |  |  |  |
| Negative, *N* (%) | 11 (52.4 %) |  |  |
| Positive, *N* (%) | 9 (42.9 %) |  |  |
| Unknown, *N* (%) | 1 (4.8 %) |  |  |
| **Lymph node status** |  |  |  |
| Positive, *N* (%) | 0 (0.0 %) |  |  |
| Negative, *N* (%) | 1 (4.8 %) |  |  |
| Unknown, *N* (%) | 20 (95.2 %) |  |  |

**Table S1:** **Clinicopathological characteristics of patients analyzed for methylation (450K).**

| **UCSC Refgene name** | **Probe ID** | **adj. *P*-value** | **mean Δβ** | **Mean β (T)** | **Mean β (NM)** | **UCSC Refgene group** | **Mapinfo^a^** | **Chr^b^** | **UCSC CGI^c^** |
| --- | --- | --- | --- | --- | --- | --- | --- | --- | --- |
| GRHL3;GRHL3;GRHL3 | cg14616251 | 2.24E-18 | 0.588 | 0.668 | 0.080 | TSS1500;Body;TSS1500 | 24648696 | 1 | chr1:24648202-24648985 |
| EPHA10 | cg11664987 | 2.60E-19 | 0.587 | 0.687 | 0.100 | Body | 38201123 | 1 | chr1:38200919-38201200 |
| BEST4 | cg04108939 | 3.31E-09 | 0.554 | 0.619 | 0.065 | Body | 45250181 | 1 | chr1:45249860-45250352 |
| FOXE3 | cg22796507 | 1.86E-12 | 0.551 | 0.707 | 0.156 | 1stExon | 47882739 | 1 | chr1:47881896-47883065 |
| SPATA6 | cg01710865 | 2.56E-06 | 0.562 | 0.662 | 0.100 | Body | 48937404 | 1 | chr1:48937304-48937683 |
| SPATA6 | cg18267506 | 2.13E-17 | 0.604 | 0.662 | 0.057 | Body | 48937445 | 1 | chr1:48937304-48937683 |
| PODN | cg04765848 | 3.55E-18 | 0.571 | 0.639 | 0.068 | TSS1500 | 53527576 | 1 | chr1:53527572-53528974 |
| SGIP1 | cg27558095 | 3.03E-13 | 0.556 | 0.649 | 0.093 | TSS1500 | 66999588 | 1 | chr1:66998728-66999060 |
| GSTM2;GSTM2 | cg16670497 | 2.15E-15 | 0.555 | 0.616 | 0.061 | Body;Body | 110210913 | 1 | chr1:110210581-110210956 |
| GSTM2;GSTM2 | cg03942855 | 5.52E-15 | 0.634 | 0.742 | 0.108 | Body;Body | 110210925 | 1 | chr1:110210581-110210956 |
| GSTM1;GSTM1 | cg06632762 | 4.06E-13 | 0.568 | 0.631 | 0.063 | Body;Body | 110230545 | 1 | chr1:110230238-110230614 |
| TBX15 | cg02177231 | 7.00E-13 | 0.580 | 0.713 | 0.133 | 5'UTR | 119529930 | 1 | chr1:119529819-119530712 |
| C1orf114;C1orf114 | cg00100121 | 2.29E-06 | 0.562 | 0.591 | 0.028 | 1stExon;5'UTR | 169396635 | 1 | chr1:169396621-169396869 |
| C1orf114;C1orf114 | cg13958426 | 5.94E-17 | 0.570 | 0.618 | 0.048 | 1stExon;5'UTR | 169396637 | 1 | chr1:169396621-169396869 |
| C1orf114 | cg23818870 | 4.40E-17 | 0.562 | 0.622 | 0.060 | TSS200 | 169396785 | 1 | chr1:169396621-169396869 |
| ESRRG | cg06634716 | 9.97E-17 | 0.569 | 0.746 | 0.176 | TSS200 | 217311151 | 1 | chr1:217310749-217311178 |
| ESRRG | cg21784383 | 1.00E-18 | 0.610 | 0.775 | 0.164 | TSS200 | 217311172 | 1 | chr1:217310749-217311178 |
| ESRRG | cg25975621 | 5.34E-16 | 0.607 | 0.742 | 0.135 | TSS200 | 217311177 | 1 | chr1:217310749-217311178 |
| LOC149134 (LINC01341) | cg15176829 | 6.41E-17 | 0.597 | 0.758 | 0.161 | TSS1500 | 246952215 | 1 | chr1:246952214-246952511 |
| LOC149134 (LINC01341) | cg03596016 | 2.43E-17 | 0.654 | 0.812 | 0.158 | TSS1500 | 246952362 | 1 | chr1:246952214-246952511 |
| EFEMP1;EFEMP1;EFEMP1;EFEMP1 | cg20786074 | 2.93E-16 | 0.550 | 0.741 | 0.191 | 1stExon;5'UTR;5'UTR;5'UTR | 56150255 | 2 | chr2:56150340-56151180 |
| EFEMP1;EFEMP1;EFEMP1 | cg16100120 | 2.33E-16 | 0.556 | 0.620 | 0.064 | TSS200;5'UTR;5'UTR | 56150475 | 2 | chr2:56150340-56151180 |
| EFEMP1;EFEMP1;EFEMP1 | cg05385513 | 6.11E-17 | 0.586 | 0.678 | 0.092 | TSS200;5'UTR;5'UTR | 56150478 | 2 | chr2:56150340-56151180 |
| C2orf55 | cg19043574 | 9.29E-15 | 0.554 | 0.665 | 0.110 | Body | 99439533 | 2 | chr2:99438297-99439884 |
| NXPH2 | cg02081701 | 1.35E-15 | 0.602 | 0.668 | 0.066 | Body | 139537197 | 2 | chr2:139537692-139538650 |
| C2orf88;C2orf88;C2orf88;C2orf88 | cg10168635 | 1.10E-15 | 0.589 | 0.727 | 0.138 | TSS1500;5'UTR;TSS1500;5'UTR | 191044980 | 2 | chr2:191044979-191045829 |
| C2orf88;C2orf88;C2orf88;C2orf88 | cg08350814 | 2.06E-07 | 0.565 | 0.590 | 0.026 | TSS1500;5'UTR;TSS1500;5'UTR | 191045309 | 2 | chr2:191044979-191045829 |
| AOX1;AOX1 | cg12627583 | 3.36E-18 | 0.603 | 0.656 | 0.053 | 1stExon;5'UTR | 201450731 | 2 | chr2:201450526-201451027 |
| AOX1;AOX1 | cg13875120 | 4.81E-15 | 0.626 | 0.673 | 0.047 | 1stExon;5'UTR | 201450743 | 2 | chr2:201450526-201451027 |
| AOX1 | cg14383422 | 1.67E-14 | 0.576 | 0.713 | 0.137 | Body | 201450962 | 2 | chr2:201450526-201451027 |
| CRYGD;CRYGD | cg22399133 | 5.41E-16 | 0.619 | 0.776 | 0.157 | 1stExon;5'UTR | 208989248 | 2 | chr2:208988834-208989269 |
| FEV | cg23108709 | 1.06E-17 | 0.567 | 0.717 | 0.150 | Body | 219847136 | 2 | chr2:219846291-219847137 |
| FEV | cg00260634 | 2.13E-17 | 0.551 | 0.632 | 0.081 | Body | 219847233 | 2 | chr2:219848919-219850541 |
| RARB;RARB;RARB;RARB | cg24396624 | 9.95E-16 | 0.688 | 0.807 | 0.119 | 5'UTR;1stExon;1stExon;5'UTR | 25469860 | 3 | NA |
| NBEAL2 | cg02534363 | 1.89E-15 | 0.639 | 0.758 | 0.119 | 3'UTR | 47050950 | 3 | chr3:47050486-47051609 |
| IL17RD | cg14936968 | 1.04E-12 | 0.551 | 0.659 | 0.108 | Body | 57198533 | 3 | chr3:57198243-57199378 |
| ADAMTS9 | cg25859972 | 4.76E-17 | 0.564 | 0.714 | 0.150 | Body | 64670515 | 3 | chr3:64671087-64673957 |
| FAM19A4;FAM19A4 | cg03186486 | 1.60E-16 | 0.557 | 0.595 | 0.038 | 5'UTR;5'UTR | 68980947 | 3 | chr3:68980783-68982035 |
| FAM19A4;FAM19A4 | cg15356923 | 2.38E-18 | 0.565 | 0.634 | 0.069 | 5'UTR;5'UTR | 68981011 | 3 | chr3:68980783-68982035 |
| PARP15 | cg19166302 | 1.88E-15 | 0.608 | 0.771 | 0.162 | 1stExon | 122296613 | 3 | chr3:122296612-122296828 |
| ALDH1L1 | cg07330230 | 2.88E-06 | 0.584 | 0.622 | 0.038 | TSS1500 | 125899925 | 3 | chr3:125898662-125899568 |
| SOX2OT | cg14311670 | 1.54E-15 | 0.631 | 0.730 | 0.099 | Body | 181444999 | 3 | chr3:181444409-181445000 |
| SOX2OT | cg20177650 | 4.96E-16 | 0.612 | 0.724 | 0.112 | Body | 181445156 | 3 | chr3:181444409-181445000 |
| BCL6;BCL6 | cg00480331 | 2.59E-14 | 0.580 | 0.693 | 0.113 | 5'UTR;TSS1500 | 187455371 | 3 | chr3:187455330-187455843 |
| CPLX1 | cg07434284 | 9.69E-16 | 0.583 | 0.721 | 0.137 | 3'UTR | 779691 | 4 | chr4:778661-780592 |
| HS3ST1 | cg01089498 | 5.73E-14 | 0.557 | 0.707 | 0.150 | 5'UTR | 11428985 | 4 | chr4:11429414-11429633 |
| HS3ST1 | cg14820199 | 2.46E-19 | 0.645 | 0.746 | 0.100 | 5'UTR | 11429415 | 4 | chr4:11429414-11429633 |
| NKX3-2 | cg12973941 | 2.50E-15 | 0.568 | 0.710 | 0.142 | 1stExon | 13545639 | 4 | chr4:13543562-13546494 |
| PROM1 | cg04203238 | 2.48E-17 | 0.590 | 0.720 | 0.130 | TSS200;TSS200 | 16085702 | 4 | chr4:16084195-16085735 |
| PROM1 | cg26260038 | 9.42E-17 | 0.633 | 0.817 | 0.184 | TSS200;TSS200 | 16085716 | 4 | chr4:16084195-16085735 |
| TBC1D1 | cg24651977 | 1.48E-17 | 0.579 | 0.671 | 0.092 | Body | 37978758 | 4 | chr4:37978702-37979595 |
| HOPX;HOPX;HOPX;HOPX;HOPX;HOPX | cg21899596 | 1.08E-16 | 0.666 | 0.790 | 0.124 | 1stExon;5'UTR;5'UTR;Body;Body;5'UTR | 57522493 | 4 | chr4:57521621-57522703 |
| UCP1 | cg05644921 | 2.89E-14 | 0.565 | 0.702 | 0.137 | Body | 141489114 | 4 | chr4:141489962-141490378 |
| C5orf49 | cg25214789 | 3.08E-16 | 0.640 | 0.677 | 0.036 | Body | 7850070 | 5 | chr5:7849945-7850439 |
| C5orf49 | cg12539796 | 4.46E-08 | 0.696 | 0.768 | 0.072 | Body | 7850203 | 5 | chr5:7849945-7850439 |
| ADAMTS12 | cg26573704 | 2.59E-15 | 0.636 | 0.692 | 0.056 | TSS200 | 33892223 | 5 | chr5:33892191-33892403 |
| EGFLAM | cg16317273 | 2.97E-15 | 0.591 | 0.678 | 0.087 | TSS1500 | 38258028 | 5 | chr5:38257825-38259136 |
| CARTPT | cg01187920 | 5.37E-16 | 0.577 | 0.721 | 0.144 | 1stExon | 71015162 | 5 | chr5:71014917-71015715 |
| SLCO4C1 | cg19788741 | 3.44E-17 | 0.585 | 0.695 | 0.110 | TSS200 | 101632310 | 5 | chr5:101632049-101632373 |
| SLCO4C1 | cg11267955 | 1.69E-16 | 0.564 | 0.651 | 0.087 | TSS200 | 101632327 | 5 | chr5:101632049-101632373 |
| SLCO4C1 | cg04621020 | 1.50E-15 | 0.559 | 0.623 | 0.064 | TSS200 | 101632341 | 5 | chr5:101632049-101632373 |
| 01-03-2003 | cg23155911 | 1.58E-20 | 0.558 | 0.680 | 0.123 | 3'UTR | 126205009 | 5 | NA |
| SPARC | cg23146663 | 2.73E-18 | 0.550 | 0.650 | 0.100 | TSS200 | 151066662 | 5 | NA |
| C5orf4;C5orf4 | cg00604840 | 2.32E-18 | 0.598 | 0.713 | 0.114 | 5'UTR;1stExon | 154230173 | 5 | NA |
| C5orf4 | cg18188717 | 5.18E-18 | 0.573 | 0.696 | 0.122 | TSS200 | 154230223 | 5 | NA |
| DOCK2 | cg08862890 | 1.77E-17 | 0.688 | 0.790 | 0.102 | Body | 169064451 | 5 | chr5:169064270-169064702 |
| DOCK2 | cg23338503 | 4.21E-18 | 0.578 | 0.653 | 0.076 | Body | 169064530 | 5 | chr5:169064270-169064702 |
| FGF18 | cg07100000 | 3.41E-17 | 0.571 | 0.683 | 0.112 | Body | 170877840 | 5 | chr5:170877798-170878210 |
| FGF18 | cg09856068 | 5.99E-06 | 0.603 | 0.680 | 0.076 | Body | 170878246 | 5 | chr5:170877798-170878210 |
| SCGB3A1 | cg21919857 | 7.90E-18 | 0.656 | 0.756 | 0.100 | Body | 180018102 | 5 | chr5:180017099-180019062 |
| SCGB3A1 | cg19897330 | 2.59E-15 | 0.567 | 0.694 | 0.127 | Body | 180018201 | 5 | chr5:180017099-180019062 |
| FLT4;FLT4 | cg00489401 | 1.45E-17 | 0.607 | 0.778 | 0.172 | Body;Body | 180075875 | 5 | chr5:180075688-180076906 |
| GMDS | cg04497116 | 3.46E-05 | 0.570 | 0.651 | 0.081 | Body | 1625574 | 6 | chr6:1624185-1625468 |
| GCM2 | cg14000361 | 4.14E-15 | 0.596 | 0.731 | 0.135 | TSS1500 | 10882336 | 6 | chr6:10882926-10883149 |
| GCM2 | cg15244049 | 7.74E-17 | 0.580 | 0.703 | 0.122 | TSS1500 | 10883376 | 6 | chr6:10881846-10882051 |
| HIST1H1A | cg10146929 | 2.72E-12 | 0.574 | 0.683 | 0.108 | 1stExon | 26017939 | 6 | chr6:26020671-26021125 |
| HIST1H3G;HIST1H2BI | cg00036011 | 5.05E-17 | 0.558 | 0.600 | 0.041 | TSS200;TSS1500 | 26271718 | 6 | chr6:26272470-26272696 |
| HLA-H | cg00157477 | 3.52E-18 | 0.602 | 0.782 | 0.180 | Body | 29855473 | 6 | chr6:29855295-29856565 |
| HCG4P6 | cg15931205 | 0.001250446 | 0.622 | 0.712 | 0.090 | TSS1500 | 29894820 | 6 | chr6:29894140-29895117 |
| HCG4P6 | cg05019905 | 3.17E-17 | 0.590 | 0.650 | 0.060 | TSS1500 | 29894831 | 6 | chr6:29894140-29895117 |
| HLA-J;NCRNA00171 | cg09003023 | 4.97E-14 | 0.555 | 0.742 | 0.187 | Body;Body | 29974253 | 6 | chr6:29974220-29975369 |
| HLA-J;NCRNA00171 | cg08879910 | 1.73E-14 | 0.603 | 0.754 | 0.152 | Body;Body | 29974319 | 6 | chr6:29974220-29975369 |
| HLA-J;NCRNA00171 | cg24725574 | 1.75E-08 | 0.606 | 0.662 | 0.056 | Body;Body | 29974717 | 6 | chr6:29974220-29975369 |
| HLA-J;NCRNA00171 | cg08163199 | 4.95E-18 | 0.622 | 0.740 | 0.117 | Body;Body | 29974858 | 6 | chr6:29974220-29975369 |
| HLA-J;NCRNA00171 | cg25318809 | 7.71E-18 | 0.609 | 0.723 | 0.114 | Body;Body | 29974863 | 6 | chr6:29974220-29975369 |
| HLA-J;NCRNA00171 | cg08325845 | 3.29E-17 | 0.574 | 0.716 | 0.142 | Body;Body | 29974886 | 6 | chr6:29974220-29975369 |
| HLA-J;NCRNA00171 | cg15726260 | 5.90E-17 | 0.560 | 0.646 | 0.086 | Body;Body | 29974900 | 6 | chr6:29974220-29975369 |
| HLA-J;NCRNA00171 | cg16794576 | 2.24E-18 | 0.598 | 0.670 | 0.073 | Body;Body | 29974971 | 6 | chr6:29974220-29975369 |
| HLA-J;NCRNA00171 | cg24666993 | 6.60E-19 | 0.608 | 0.699 | 0.090 | Body;Body | 29974991 | 6 | chr6:29974220-29975369 |
| HLA-J;NCRNA00171 | cg12976581 | 5.35E-19 | 0.557 | 0.605 | 0.048 | Body;Body | 29975032 | 6 | chr6:29974220-29975369 |
| HLA-J;NCRNA00171 | cg21330423 | 1.79E-18 | 0.669 | 0.727 | 0.058 | Body;Body | 29975068 | 6 | chr6:29974220-29975369 |
| HLA-J;NCRNA00171 | cg05998089 | 3.48E-19 | 0.586 | 0.649 | 0.063 | Body;Body | 29975141 | 6 | chr6:29974220-29975369 |
| PPT2;PPT2 | cg14130039 | 5.32E-16 | 0.647 | 0.758 | 0.112 | TSS200;TSS1500 | 32121225 | 6 | chr6:32121829-32122529 |
| TULP1 | cg15219228 | 3.68E-11 | 0.566 | 0.672 | 0.106 | Body | 35479628 | 6 | chr6:35479388-35479678 |
| TFAP2B | cg27260772 | 2.56E-13 | 0.583 | 0.751 | 0.168 | Body | 50791202 | 6 | chr6:50791110-50791573 |
| EYA4;EYA4;EYA4 | cg11664500 | 2.30E-18 | 0.553 | 0.592 | 0.039 | TSS200;TSS200;TSS200 | 133562479 | 6 | chr6:133562086-133563586 |
| OLIG3 | cg01972751 | 1.26E-17 | 0.578 | 0.753 | 0.175 | 1stExon | 137814728 | 6 | chr6:137814355-137815202 |
| FBXO30 | cg23095615 | 4.07E-15 | 0.608 | 0.660 | 0.051 | TSS1500 | 146136563 | 6 | chr6:146136325-146136564 |
| FBXO30 | cg09094393 | 2.01E-15 | 0.614 | 0.714 | 0.100 | TSS1500 | 146136749 | 6 | chr6:146135434-146136165 |
| SOSTDC1 | cg06363129 | 2.98E-18 | 0.571 | 0.802 | 0.231 | TSS200 | 16505602 | 7 | NA |
| SOSTDC1 | cg06126713 | 2.68E-17 | 0.579 | 0.680 | 0.101 | TSS200 | 16505617 | 7 | NA |
| HOXA7;HOXA7 | cg07302069 | 1.05E-06 | 0.569 | 0.649 | 0.079 | 5'UTR;1stExon | 27196286 | 7 | chr7:27195601-27196567 |
| LOC646999 | cg06890747 | 1.69E-15 | 0.557 | 0.737 | 0.179 | Body | 39649290 | 7 | chr7:39649253-39649510 |
| PON3 | cg08898155 | 4.77E-20 | 0.580 | 0.687 | 0.107 | TSS1500 | 95026097 | 7 | chr7:95025559-95026122 |
| TAC1;TAC1;TAC1;TAC1 | cg09910635 | 1.28E-17 | 0.592 | 0.768 | 0.176 | TSS1500;TSS1500;TSS1500;TSS1500 | 97361026 | 7 | chr7:97361132-97363018 |
| TAC1;TAC1;TAC1;TAC1 | cg09236284 | 6.14E-18 | 0.632 | 0.719 | 0.088 | TSS200;TSS200;TSS200;TSS200 | 97361114 | 7 | chr7:97361132-97363018 |
| TAC1;TAC1;TAC1;TAC1 | cg01287975 | 6.41E-16 | 0.648 | 0.815 | 0.166 | TSS200;TSS200;TSS200;TSS200 | 97361241 | 7 | chr7:97361132-97363018 |
| TAC1;TAC1;TAC1;TAC1 | cg17437939 | 6.20E-17 | 0.678 | 0.820 | 0.142 | TSS200;TSS200;TSS200;TSS200 | 97361252 | 7 | chr7:97361132-97363018 |
| TAC1;TAC1;TAC1;TAC1;TAC1;TAC1;TAC1;TAC1 | cg16288089 | 1.30E-15 | 0.606 | 0.732 | 0.126 | 5'UTR;1stExon;1stExon;1stExon;5'UTR;5'UTR;1stExon;5'UTR | 97361408 | 7 | chr7:97361132-97363018 |
| ACHE;ACHE | cg04156293 | 4.87E-18 | 0.559 | 0.668 | 0.109 | Body;Body | 100488942 | 7 | chr7:100489981-100490365 |
| CAV2;CAV2 | cg25274503 | 2.51E-17 | 0.581 | 0.619 | 0.038 | Body;Body | 116140128 | 7 | chr7:116139774-116140352 |
| CAV2;CAV2 | cg16260298 | 4.77E-20 | 0.600 | 0.642 | 0.043 | Body;Body | 116140342 | 7 | chr7:116139774-116140352 |
| CFTR | cg21461649 | 1.79E-12 | 0.565 | 0.722 | 0.157 | TSS200 | 117119938 | 7 | NA |
| CFTR | cg00735923 | 2.20E-05 | 0.580 | 0.771 | 0.191 | TSS200 | 117119963 | 7 | NA |
| SND1 (LRRC4) | cg09296001 | 6.22E-16 | 0.628 | 0.751 | 0.123 | Body | 127672564 | 7 | chr7:127671158-127672853 |
| FAM115A (TCAF1) | cg03514404 | 8.14E-08 | 0.662 | 0.740 | 0.077 | 5'UTR | 143579665 | 7 | chr7:143582125-143582610 |
| FAM115A (TCAF1) | cg01030534 | 5.48E-18 | 0.671 | 0.793 | 0.121 | 5'UTR | 143579698 | 7 | chr7:143582125-143582610 |
| FAM115A (TCAF1) | cg03225210 | 5.98E-16 | 0.567 | 0.660 | 0.094 | 5'UTR | 143579951 | 7 | chr7:143582125-143582610 |
| FAM115A (TCAF1) | cg02245020 | 7.35E-18 | 0.705 | 0.765 | 0.060 | 5'UTR | 143580147 | 7 | chr7:143582125-143582610 |
| FAM115A (TCAF1) | cg16807089 | 2.04E-16 | 0.614 | 0.708 | 0.095 | 5'UTR | 143582499 | 7 | chr7:143582125-143582610 |
| KCNH2;KCNH2;KCNH2 | cg20883831 | 1.46E-17 | 0.664 | 0.718 | 0.054 | TSS200;Body;Body | 150652948 | 7 | chr7:150652807-150653080 |
| KCNH2;KCNH2;KCNH2 | cg18423852 | 1.36E-19 | 0.564 | 0.660 | 0.096 | TSS200;Body;Body | 150653001 | 7 | chr7:150652807-150653080 |
| KCNH2;KCNH2;KCNH2 | cg15472092 | 2.53E-19 | 0.638 | 0.699 | 0.061 | TSS200;Body;Body | 150653079 | 7 | chr7:150652807-150653080 |
| PTPRN2;PTPRN2;PTPRN2 | cg16964025 | 2.60E-19 | 0.567 | 0.653 | 0.086 | Body;Body;Body | 157482215 | 7 | chr7:157476886-157486719 |
| TNFRSF10C | cg26029345 | 4.87E-13 | 0.615 | 0.690 | 0.075 | Body | 22960820 | 8 | chr8:22960384-22960927 |
| FGFR1;FGFR1;FGFR1;FGFR1;FGFR1;FGFR1;FGFR1 | cg13123964 | 5.67E-13 | 0.576 | 0.652 | 0.076 | 5'UTR;5'UTR;5'UTR;5'UTR;5'UTR;5'UTR;5'UTR | 38323238 | 8 | chr8:38323719-38324070 |
| RGS20 | cg03348397 | 8.90E-14 | 0.552 | 0.648 | 0.095 | Body | 54789648 | 8 | chr8:54789581-54790665 |
| RGS20;RGS20 | cg02639634 | 1.15E-17 | 0.592 | 0.688 | 0.096 | Body;TSS1500 | 54792161 | 8 | chr8:54791845-54795141 |
| LYN;LYN | cg17643864 | 5.15E-14 | 0.551 | 0.686 | 0.135 | 5'UTR;5'UTR | 56852148 | 8 | NA |
| FAM110B | cg13247663 | 1.79E-18 | 0.619 | 0.772 | 0.154 | 5'UTR | 59058254 | 8 | chr8:59058167-59059414 |
| CA3 | cg18674980 | 8.79E-16 | 0.588 | 0.684 | 0.096 | TSS1500 | 86350581 | 8 | chr8:86350765-86351196 |
| ENPP2;ENPP2;ENPP2 | cg06998282 | 4.22E-07 | 0.577 | 0.646 | 0.070 | TSS1500;TSS1500;TSS1500 | 120651398 | 8 | NA |
| MOBKL2B | cg22262168 | 8.71E-12 | 0.560 | 0.647 | 0.087 | 5'UTR | 27528999 | 9 | chr9:27528976-27529885 |
| C9orf129;C9orf129 | cg10065823 | 1.85E-15 | 0.561 | 0.740 | 0.180 | 1stExon;5'UTR | 96108467 | 9 | chr9:96108466-96108992 |
| PALM2-AKAP2;AKAP2;PALM2-AKAP2 | cg15083233 | 9.46E-16 | 0.665 | 0.806 | 0.141 | Body;TSS1500;Body | 112810402 | 9 | chr9:112810858-112811128 |
| ITIH5;ITIH5 | cg10119075 | 2.39E-16 | 0.580 | 0.654 | 0.074 | TSS1500;TSS1500 | 7709738 | 10 | NA |
| RASGEF1A | cg02636041 | 3.33E-05 | 0.679 | 0.705 | 0.026 | Body | 43698008 | 10 | chr10:43697777-43698177 |
| ALOX5 | cg21981270 | 2.52E-11 | 0.661 | 0.727 | 0.066 | Body | 45914525 | 10 | chr10:45914374-45914883 |
| ALOX5 | cg10069493 | 9.45E-13 | 0.629 | 0.760 | 0.131 | Body | 45914688 | 10 | chr10:45914374-45914883 |
| ALOX5 | cg00675569 | 2.92E-05 | 0.600 | 0.661 | 0.061 | Body | 45914840 | 10 | chr10:45914374-45914883 |
| CYP26C1 | cg05127821 | 5.27E-07 | 0.632 | 0.736 | 0.104 | Body | 94822908 | 10 | chr10:94820026-94823252 |
| SLIT1 | cg13261825 | 1.10E-15 | 0.555 | 0.695 | 0.140 | Body | 98939742 | 10 | NA |
| TLX1 | cg14861089 | 5.91E-17 | 0.555 | 0.701 | 0.146 | Body | 102895043 | 10 | chr10:102893660-102895059 |
| EFCAB4A | cg22773555 | 1.77E-11 | 0.566 | 0.663 | 0.097 | Body | 830233 | 11 | chr11:829284-833558 |
| CDKN1C;CDKN1C;CDKN1C | cg17265994 | 1.28E-13 | 0.561 | 0.725 | 0.164 | 3'UTR;3'UTR;3'UTR | 2905024 | 11 | chr11:2905023-2907024 |
| OSBPL5;OSBPL5;OSBPL5 | cg23357130 | 0.013315425 | 0.553 | 0.677 | 0.124 | 5'UTR;5'UTR;5'UTR | 3181928 | 11 | chr11:3181574-3182120 |
| FIBIN;FIBIN | cg04622802 | 1.99E-17 | 0.555 | 0.666 | 0.112 | 5'UTR;1stExon | 27015872 | 11 | NA |
| C11orf95 | cg09907758 | 3.35E-16 | 0.557 | 0.726 | 0.169 | TSS1500 | 63537459 | 11 | chr11:63535652-63537435 |
| GSTP1 | cg09038676 | 8.79E-12 | 0.584 | 0.676 | 0.091 | Body | 67351608 | 11 | chr11:67350928-67351953 |
| GSTP1 | cg11566244 | 6.44E-10 | 0.562 | 0.699 | 0.137 | Body | 67351786 | 11 | chr11:67350928-67351953 |
| SHANK2 | cg04505435 | 8.05E-15 | 0.558 | 0.710 | 0.151 | Body | 70672511 | 11 | chr11:70672834-70673055 |
| LOC100128239 | cg20771240 | 2.59E-14 | 0.565 | 0.669 | 0.104 | Body | 133906693 | 11 | chr11:133906735-133907359 |
| LOC100128239 | cg06769296 | 4.27E-15 | 0.581 | 0.689 | 0.108 | Body | 133906761 | 11 | chr11:133906735-133907359 |
| BCAT1 | cg20399616 | 4.18E-12 | 0.561 | 0.634 | 0.073 | Body | 25055967 | 12 | chr12:25055599-25056246 |
| GRASP | cg04034767 | 2.29E-14 | 0.637 | 0.710 | 0.073 | 1stExon | 52400907 | 12 | chr12:52400467-52401696 |
| GRASP | cg00817367 | 2.74E-16 | 0.684 | 0.793 | 0.109 | Body | 52401214 | 12 | chr12:52400467-52401696 |
| ACSS3;ACSS3 | cg13273396 | 2.91E-14 | 0.631 | 0.697 | 0.065 | 1stExon;5'UTR | 81471867 | 12 | chr12:81471569-81472119 |
| DUSP6;DUSP6 | cg05769889 | 8.80E-17 | 0.572 | 0.690 | 0.118 | Body;Body | 89744701 | 12 | chr12:89745168-89748144 |
| USP44;USP44 | cg22538054 | 5.26E-16 | 0.623 | 0.684 | 0.061 | 5'UTR;5'UTR | 95941988 | 12 | chr12:95941906-95942979 |
| TXNRD1 | cg17395064 | 8.46E-17 | 0.561 | 0.667 | 0.106 | TSS200 | 104609476 | 12 | chr12:104609397-104610172 |
| CHST11 | cg23855505 | 9.14E-18 | 0.671 | 0.762 | 0.091 | Body | 104852439 | 12 | chr12:104850253-104852395 |
| CHST11 | cg22260952 | 6.04E-07 | 0.650 | 0.711 | 0.061 | Body | 104852446 | 12 | chr12:104850253-104852395 |
| MSI1 | cg25561140 | 1.54E-16 | 0.561 | 0.708 | 0.147 | Body | 120799725 | 12 | chr12:120799372-120799912 |
| MSI1 | cg20569203 | 3.13E-18 | 0.558 | 0.679 | 0.120 | Body | 120799911 | 12 | chr12:120799372-120799912 |
| KLHL1;KLHL1;ATXN8OS | cg20523861 | 3.13E-18 | 0.610 | 0.782 | 0.172 | 1stExon;5'UTR;Body | 70682324 | 13 | chr13:70681732-70682219 |
| CLDN10;CLDN10;CLDN10 | cg09469554 | 3.56E-18 | 0.598 | 0.670 | 0.071 | TSS200;Body;Body | 96204917 | 13 | chr13:96204691-96205496 |
| CLDN10;CLDN10;CLDN10;CLDN10 | cg25032595 | 9.30E-14 | 0.553 | 0.740 | 0.187 | 5'UTR;1stExon;Body;Body | 96204978 | 13 | chr13:96204691-96205496 |
| NDRG2;NDRG2;NDRG2;NDRG2;NDRG2;NDRG2;NDRG2;NDRG2 | cg20902783 | 9.26E-17 | 0.559 | 0.699 | 0.141 | TSS1500;TSS200;TSS200;TSS1500;TSS1500;TSS1500;TSS200;TSS200 | 21494071 | 14 | chr14:21492735-21494270 |
| SALL2 | cg00800229 | 9.51E-19 | 0.565 | 0.612 | 0.047 | 1stExon | 22004993 | 14 | NA |
| EFS;EFS | cg16585972 | 5.56E-15 | 0.551 | 0.635 | 0.084 | TSS1500;TSS1500 | 23835212 | 14 | chr14:23834435-23835947 |
| EFS;EFS | cg17205324 | 9.28E-16 | 0.558 | 0.700 | 0.142 | TSS1500;TSS1500 | 23835595 | 14 | chr14:23834435-23835947 |
| ADCY4 | cg23179456 | 2.01E-15 | 0.637 | 0.714 | 0.077 | TSS200 | 24803873 | 14 | chr14:24803678-24804353 |
| ADCY4 | cg13631572 | 8.63E-17 | 0.563 | 0.642 | 0.079 | TSS200 | 24803903 | 14 | chr14:24803678-24804353 |
| ADCY4 | cg16215203 | 3.50E-18 | 0.605 | 0.692 | 0.086 | TSS200 | 24803917 | 14 | chr14:24803678-24804353 |
| ADCY4 | cg25556905 | 3.29E-07 | 0.613 | 0.655 | 0.042 | TSS200 | 24803925 | 14 | chr14:24803678-24804353 |
| ADCY4 | cg12265829 | 2.39E-19 | 0.655 | 0.736 | 0.081 | TSS200 | 24804022 | 14 | chr14:24803678-24804353 |
| ADCY4 | cg14287235 | 7.05E-17 | 0.555 | 0.647 | 0.093 | TSS1500 | 24804339 | 14 | chr14:24803678-24804353 |
| SSTR1 | cg16190266 | 6.90E-17 | 0.577 | 0.759 | 0.182 | 3'UTR | 38680855 | 14 | chr14:38678245-38680937 |
| SLC8A3;SLC8A3;SLC8A3;SLC8A3 | cg23241781 | 1.70E-15 | 0.561 | 0.655 | 0.094 | 5'UTR;5'UTR;5'UTR;5'UTR | 70653964 | 14 | chr14:70655095-70656077 |
| FBLN5;FBLN5 | cg21883802 | 2.12E-15 | 0.586 | 0.662 | 0.076 | 5'UTR;1stExon | 92413706 | 14 | chr14:92413581-92414324 |
| GREM1 | cg18145505 | 1.01E-15 | 0.600 | 0.745 | 0.146 | TSS1500 | 33009498 | 15 | chr15:33009530-33011696 |
| SHF | cg24033558 | 6.30E-11 | 0.600 | 0.712 | 0.112 | Body | 45479755 | 15 | chr15:45479509-45480498 |
| ANXA2;ANXA2;ANXA2;ANXA2 | cg20118643 | 1.99E-16 | 0.570 | 0.638 | 0.068 | TSS1500;TSS1500;TSS1500;TSS1500 | 60690852 | 15 | chr15:60690708-60690930 |
| ITGA11 | cg14188232 | 2.97E-15 | 0.571 | 0.661 | 0.089 | Body | 68724065 | 15 | chr15:68723367-68724691 |
| CYP11A1;CYP11A1;CYP11A1 | cg17588266 | 2.37E-15 | 0.594 | 0.692 | 0.099 | 1stExon;Body;5'UTR | 74658330 | 15 | chr15:74658038-74658574 |
| CYP11A1;CYP11A1;CYP11A1 | cg16332610 | 3.01E-16 | 0.661 | 0.747 | 0.086 | 1stExon;Body;5'UTR | 74658547 | 15 | chr15:74658038-74658574 |
| RHCG | cg18726691 | 3.14E-17 | 0.636 | 0.733 | 0.097 | 1stExon | 90039613 | 15 | chr15:90039464-90039984 |
| RHCG | cg01837657 | 5.26E-16 | 0.620 | 0.740 | 0.120 | TSS200 | 90039822 | 15 | chr15:90039464-90039984 |
| GPRC5B | cg07519235 | 4.76E-16 | 0.688 | 0.787 | 0.099 | 5'UTR | 19894978 | 16 | chr16:19894965-19897156 |
| CRYM | cg16596604 | 5.59E-18 | 0.597 | 0.695 | 0.098 | 5'UTR | 21294947 | 16 | chr16:21294980-21295414 |
| PRKCB;PRKCB;PRKCB;PRKCB | cg03306374 | 3.36E-08 | 0.603 | 0.658 | 0.055 | 1stExon;5'UTR;5'UTR;1stExon | 23847325 | 16 | chr16:23846941-23848102 |
| HS3ST4 | cg23235241 | 5.15E-14 | 0.575 | 0.620 | 0.045 | Body | 25704505 | 16 | chr16:25702955-25704631 |
| CYBA | cg03787864 | 1.79E-18 | 0.582 | 0.646 | 0.064 | Body | 88717134 | 16 | chr16:88716989-88717606 |
| CYBA | cg26537639 | 3.87E-14 | 0.685 | 0.882 | 0.197 | 1stExon | 88717374 | 16 | chr16:88716989-88717606 |
| CYBA | cg04879832 | 2.25E-05 | 0.568 | 0.685 | 0.117 | 1stExon;5'UTR | 88717456 | 16 | chr16:88716989-88717606 |
| CYBA | cg08843517 | 3.48E-19 | 0.637 | 0.820 | 0.183 | TSS200 | 88717464 | 16 | chr16:88716989-88717606 |
| CYBA | cg03125427 | 4.96E-16 | 0.588 | 0.672 | 0.084 | TSS200 | 88717482 | 16 | chr16:88716989-88717606 |
| CYBA | cg24046411 | 3.94E-16 | 0.609 | 0.725 | 0.117 | TSS200 | 88717646 | 16 | chr16:88716989-88717606 |
| CYBA | cg19790294 | 1.93E-16 | 0.565 | 0.664 | 0.099 | TSS1500 | 88717755 | 16 | chr16:88716989-88717606 |
| RARA;RARA | cg00236832 | 4.27E-18 | 0.558 | 0.616 | 0.058 | 5'UTR;1stExon | 38465489 | 17 | NA |
| RARA;RARA | cg26654286 | 2.39E-17 | 0.621 | 0.678 | 0.057 | 5'UTR;1stExon | 38465510 | 17 | NA |
| RND2 | cg05270634 | 9.92E-06 | 0.552 | 0.612 | 0.060 | 1stExon | 41177445 | 17 | chr17:41177336-41177593 |
| NBR1;TMEM106A;NBR1;NBR1 | cg08377924 | 1.87E-05 | 0.551 | 0.604 | 0.053 | 3'UTR;TSS1500;3'UTR;3'UTR | 41363628 | 17 | chr17:41363727-41364273 |
| TMEM106A | cg19548479 | 6.43E-15 | 0.651 | 0.704 | 0.053 | TSS200 | 41363737 | 17 | chr17:41363727-41364273 |
| TMEM106A | cg24940138 | 1.40E-05 | 0.565 | 0.624 | 0.059 | TSS200 | 41363741 | 17 | chr17:41363727-41364273 |
| TMEM106A | cg18222083 | 8.45E-06 | 0.670 | 0.798 | 0.127 | 5'UTR | 41364007 | 17 | chr17:41363727-41364273 |
| RUNDC3A;RUNDC3A;RUNDC3A | cg01635086 | 1.16E-18 | 0.641 | 0.669 | 0.028 | Body;Body;Body | 42386326 | 17 | chr17:42385810-42386393 |
| ACBD4;ACBD4;ACBD4;ACBD4 | cg00677574 | 3.55E-18 | 0.641 | 0.758 | 0.118 | 3'UTR;3'UTR;3'UTR;3'UTR | 43221327 | 17 | chr17:43221927-43222388 |
| HLF | cg05452524 | 5.85E-15 | 0.579 | 0.609 | 0.030 | Body | 53343029 | 17 | chr17:53342198-53343061 |
| HLF | cg01451391 | 4.74E-14 | 0.569 | 0.646 | 0.077 | Body | 53343561 | 17 | chr17:53342198-53343061 |
| B3GNTL1 | cg14621217 | 1.03E-17 | 0.555 | 0.653 | 0.099 | Body | 80944134 | 17 | NA |
| KCTD1 | cg26704078 | 2.63E-16 | 0.556 | 0.805 | 0.249 | 5'UTR | 24131115 | 18 | chr18:24126780-24131138 |
| PGLYRP2 | cg18652900 | 1.28E-14 | 0.574 | 0.727 | 0.153 | Body | 15580445 | 19 | chr19:15580382-15580863 |
| TPM4;TPM4 | cg16232979 | 1.12E-09 | 0.568 | 0.695 | 0.127 | Body;Body | 16187631 | 19 | chr19:16186789-16188275 |
| HIF3A | cg14117138 | 2.08E-06 | 0.622 | 0.740 | 0.118 | TSS1500 | 46800085 | 19 | chr19:46800053-46800603 |
| HIF3A;HIF3A | cg10594090 | 1.79E-15 | 0.561 | 0.639 | 0.078 | TSS200;TSS1500 | 46800198 | 19 | chr19:46800053-46800603 |
| HIF3A;HIF3A | cg05286653 | 1.85E-16 | 0.562 | 0.731 | 0.169 | Body;TSS1500 | 46800602 | 19 | chr19:46800053-46800603 |
| CCDC8 | cg03576469 | 5.98E-19 | 0.606 | 0.674 | 0.069 | TSS200 | 46917061 | 19 | chr19:46915311-46915802 |
| CYTH2;CYTH2 | cg01532436 | 1.92E-15 | 0.570 | 0.666 | 0.095 | 3'UTR;3'UTR | 48983942 | 19 | chr19:48983562-48984175 |
| CYTH2;CYTH2 | cg02590401 | 4.04E-15 | 0.591 | 0.700 | 0.109 | 3'UTR;3'UTR | 48984071 | 19 | chr19:48983562-48984175 |
| KLK13 | cg20104456 | 5.82E-19 | 0.590 | 0.701 | 0.111 | TSS200 | 51568523 | 19 | chr19:51568154-51568412 |
| ZNF577;ZNF577;ZNF577 | cg03562414 | 1.85E-14 | 0.570 | 0.702 | 0.132 | Body;5'UTR;5'UTR | 52391078 | 19 | chr19:52390841-52391368 |
| ZNF577;ZNF577;ZNF577 | cg16731240 | 1.38E-14 | 0.633 | 0.760 | 0.126 | TSS200;TSS200;TSS200 | 52391250 | 19 | chr19:52390841-52391368 |
| ZNF577;ZNF577;ZNF577 | cg23010048 | 1.16E-14 | 0.566 | 0.744 | 0.179 | TSS200;TSS200;TSS200 | 52391257 | 19 | chr19:52390841-52391368 |
| ZNF577;ZNF577;ZNF577 | cg11269599 | 2.80E-12 | 0.565 | 0.647 | 0.082 | TSS200;TSS200;TSS200 | 52391304 | 19 | chr19:52390841-52391368 |
| ZNF808 | cg22455450 | 1.93E-15 | 0.579 | 0.727 | 0.148 | 5'UTR | 53038972 | 19 | chr19:53039077-53039920 |
| ZNF154;ZNF154 | cg05661282 | 8.86E-17 | 0.557 | 0.673 | 0.116 | 5'UTR;1stExon | 58220370 | 19 | chr19:58220189-58220517 |
| LOC284798;LOC284798;LOC284798 | cg04299389 | 2.91E-11 | 0.568 | 0.665 | 0.096 | Body;TSS1500;Body | 25129296 | 20 | chr20:25128764-25129610 |
| GDAP1L1 | cg04448487 | 9.29E-15 | 0.561 | 0.652 | 0.092 | TSS200 | 42875777 | 20 | chr20:42875764-42876184 |
| GDAP1L1 | cg09994356 | 1.33E-16 | 0.608 | 0.673 | 0.065 | TSS200 | 42875779 | 20 | chr20:42875764-42876184 |
| WFDC2 | cg00980978 | 1.06E-11 | 0.550 | 0.603 | 0.052 | Body | 44098724 | 20 | chr20:44098280-44099536 |
| CLDN5;CLDN5;CLDN5 | cg20486569 | 4.01E-18 | 0.613 | 0.745 | 0.132 | 1stExon;5'UTR;5'UTR | 19512228 | 22 | chr22:19510871-19512254 |
| TTC28 | cg04294888 | 1.06E-06 | 0.554 | 0.618 | 0.065 | Body | 28839249 | 22 | NA |
| NHS | cg00409000 | 5.98E-17 | 0.687 | 0.735 | 0.049 | Body | 17395816 | X | chrX:17393159-17395817 |
| NHS | cg24605338 | 2.27E-17 | 0.636 | 0.879 | 0.242 | Body | 17395856 | X | chrX:17393159-17395817 |
| MAP7D2;MAP7D2;MAP7D2;MAP7D2 | cg06016684 | 6.75E-07 | 0.574 | 0.612 | 0.038 | Body;Body;Body;5'UTR | 20134557 | X | chrX:20134203-20135350 |
| ARX;ARX | cg25053900 | 9.89E-17 | 0.583 | 0.652 | 0.069 | 1stExon;5'UTR | 25033890 | X | chrX:25033664-25034712 |
| ARX;ARX | cg17036062 | 2.61E-19 | 0.631 | 0.693 | 0.063 | 1stExon;5'UTR | 25034037 | X | chrX:25033664-25034712 |
| ARX | cg21729122 | 8.79E-16 | 0.552 | 0.656 | 0.104 | TSS1500 | 25034488 | X | chrX:25033664-25034712 |
| SYN1;SYN1 | cg25153755 | 7.69E-14 | 0.575 | 0.689 | 0.113 | Body;Body | 47433716 | X | chrX:47433414-47434124 |
| SHROOM4;SHROOM4 | cg18110168 | 1.96E-06 | 0.564 | 0.609 | 0.044 | TSS200;TSS200 | 50557055 | X | chrX:50556224-50557179 |
| SHROOM4;SHROOM4 | cg05152874 | 2.66E-06 | 0.550 | 0.580 | 0.030 | TSS200;TSS200 | 50557122 | X | chrX:50556224-50557179 |
| KLF8;KLF8 | cg19505129 | 5.86E-17 | 0.559 | 0.643 | 0.084 | TSS200;TSS200 | 56258695 | X | chrX:56258951-56259159 |
| KLF8;KLF8 | cg05559795 | 6.52E-15 | 0.555 | 0.616 | 0.061 | TSS200;TSS200 | 56258812 | X | chrX:56258951-56259159 |
| KLF8;KLF8;KLF8;KLF8 | cg09195491 | 2.94E-16 | 0.573 | 0.608 | 0.034 | 5'UTR;1stExon;1stExon;5'UTR | 56258900 | X | chrX:56258951-56259159 |
| KLF8;KLF8;KLF8;KLF8 | cg03610137 | 7.67E-20 | 0.576 | 0.614 | 0.038 | 5'UTR;1stExon;1stExon;5'UTR | 56259040 | X | chrX:56258951-56259159 |
| KLF8;KLF8;KLF8;KLF8 | cg03834574 | 5.28E-19 | 0.627 | 0.682 | 0.055 | 5'UTR;1stExon;1stExon;5'UTR | 56259094 | X | chrX:56258951-56259159 |
| KLF8;KLF8;KLF8 | cg06457357 | 3.48E-07 | 0.625 | 0.716 | 0.091 | 5'UTR;1stExon;5'UTR | 56259243 | X | chrX:56258951-56259159 |
| KLF8;KLF8;KLF8 | cg06655100 | 2.26E-16 | 0.596 | 0.704 | 0.108 | 5'UTR;1stExon;5'UTR | 56259373 | X | chrX:56258951-56259159 |
| MSN | cg22771999 | 1.88E-15 | 0.588 | 0.683 | 0.095 | Body | 64888437 | X | chrX:64887549-64887837 |
| SLC16A2 | cg15236541 | 3.17E-13 | 0.573 | 0.648 | 0.075 | Body | 73642618 | X | chrX:73642494-73642766 |
| SLC16A2 | cg22750044 | 1.87E-13 | 0.556 | 0.663 | 0.106 | Body | 73642662 | X | chrX:73642494-73642766 |
| GPRASP1;GPRASP1;GPRASP1 | cg24818939 | 8.64E-06 | 0.550 | 0.601 | 0.050 | TSS200;TSS200;TSS200 | 101906109 | X | chrX:101906001-101907017 |
| COL4A6 | cg05064925 | 1.64E-17 | 0.597 | 0.670 | 0.073 | 1stExon;Body;5'UTR | 107681469 | X | NA |
| FGF13;FGF13;FGF13;FGF13;FGF13;FGF13 | cg05196026 | 4.69E-14 | 0.571 | 0.628 | 0.056 | Body;1stExon;Body;Body;Body;Body | 137793096 | X | chrX:137793095-137794010 |
| PDZD4 | cg06616051 | 1.59E-15 | 0.573 | 0.793 | 0.220 | Body | 153094332 | X | chrX:153094109-153094620 |
| PDZD4 | cg09329826 | 2.15E-17 | 0.614 | 0.722 | 0.108 | Body | 153094594 | X | chrX:153094109-153094620 |
| TMLHE | cg00763725 | 2.77E-12 | 0.551 | 0.601 | 0.050 | TSS200 | 154842689 | X | chrX:154842112-154842719 |
| PRKY | cg05618150 | 9.77E-12 | 0.597 | 0.670 | 0.073 | TSS1500 | 7140718 | Y | chrY:7141521-7143224 |

**Table S2:** **Shortlist of DMCs with mean Δβ>|0.55| and adj. *P*<0.05 for candidate selection.** Yellow: Selected candidate genes. Annotations according to Illumina GenomeStudio. ^a^Coordinates of investigated CpG site (hg19). ^b^Chromosome. ^c^Coordinates of annotated CpG island (hg19). NA: Not applicable.

|  |  |  | **DNA methylation (450K)** | | | | | | | | **Gene expression** | | | |
| --- | --- | --- | --- | --- | --- | --- | --- | --- | --- | --- | --- | --- | --- | --- |
|  |  |  | **Discovery (21 T *vs.* 21 NM)** | | | | **TCGA (297 T *vs.* 34 NM)** | | | | **In-house (14 T *vs.* 12 NM)** | | **TCGA (297 T *vs.* 34 NM)** | |
| **Gene** | **Probe no.^a^** | **Probe ID** | **T mean β** | **NM mean β** | **mean Δβ** | **Adj. *P*-value** | **T mean β** | **NM mean β** | **mean Δβ** | **Adj. *P*-value** | **log2 FC** | **Adj. *P*-value** | **log2 FC** | **Adj. *P*-value** |
| **COL4A6** |  |  |  |  |  |  |  |  |  |  | -1.620 | 9.01E-08 | -1.678 | 1.9E-16 |
|  | **1** | cg24275475 | 0.577 | 0.089 | 0.489 | 2.30E-16 | 0.581 | 0.156 | 0.425 | 1.42E-29 |  |  |  |  |
|  | **2** | cg05064925 | 0.670 | 0.073 | 0.597 | 1.64E-17 | 0.629 | 0.147 | 0.482 | 2.17E-33 |  |  |  |  |
|  | **3** | cg12737160 | 0.497 | 0.033 | 0.464 | 1.46E-17 | 0.554 | 0.180 | 0.374 | 6.93E-25 |  |  |  |  |
|  | **4** | cg00815013 | 0.481 | 0.041 | 0.440 | 2.06E-18 | 0.544 | 0.202 | 0.342 | 3.36E-24 |  |  |  |  |
|  | **5** | cg13643594 | 0.751 | 0.345 | 0.407 | 3.13E-12 | 0.654 | 0.454 | 0.200 | 3.29E-15 |  |  |  |  |
| **CYBA** |  |  |  |  |  |  |  |  |  |  | -1.224 | 3.04E-03 | -0.646 | 3.85E-04 |
|  | **1** | cg01612095 | 0.670 | 0.899 | -0.229 | 1.24E-12 | NA | NA | NA | NA |  |  |  |  |
|  | **2** | cg03787864 | 0.646 | 0.064 | 0.582 | 1.79E-18 | 0.610 | 0.150 | 0.461 | 5.47E-29 |  |  |  |  |
|  | **3** | cg26537639 | 0.882 | 0.197 | 0.685 | 3.87E-14 | 0.700 | 0.340 | 0.361 | 1.28E-36 |  |  |  |  |
|  | **4** | cg04879832 | 0.685 | 0.117 | 0.568 | 2.25E-05 | 0.584 | 0.184 | 0.399 | 3.24E-39 |  |  |  |  |
|  | **5** | cg08843517 | 0.820 | 0.183 | 0.637 | 3.48E-19 | 0.680 | 0.190 | 0.490 | 6.80E-39 |  |  |  |  |
|  | **6** | cg09025501 | 0.617 | 0.091 | 0.526 | 3.55E-18 | 0.515 | 0.152 | 0.363 | 1.07E-27 |  |  |  |  |
|  | **7** | cg03125427 | 0.672 | 0.084 | 0.588 | 4.96E-16 | 0.571 | 0.130 | 0.441 | 1.01E-24 |  |  |  |  |
|  | **8** | cg08177833 | 0.539 | 0.054 | 0.485 | 4.28E-15 | 0.485 | 0.125 | 0.360 | 6.86E-30 |  |  |  |  |
|  | **9** | cg00054525 | 0.514 | 0.094 | 0.420 | 1.12E-13 | 0.494 | 0.150 | 0.345 | 6.95E-30 |  |  |  |  |
|  | **10** | cg10246531 | 0.736 | 0.226 | 0.510 | 7.92E-18 | 0.654 | 0.288 | 0.367 | 2.84E-28 |  |  |  |  |
|  | **11** | cg24046411 | 0.725 | 0.117 | 0.609 | 3.94E-16 | 0.637 | 0.282 | 0.355 | 7.78E-28 |  |  |  |  |
|  | **12** | cg05744675 | 0.569 | 0.067 | 0.502 | 2.81E-19 | 0.490 | 0.134 | 0.355 | 1.70E-22 |  |  |  |  |
|  | **13** | cg19790294 | 0.664 | 0.099 | 0.565 | 1.93E-16 | 0.589 | 0.276 | 0.313 | 1.03E-26 |  |  |  |  |
|  | **14** | cg03870138 | 0.482 | 0.088 | 0.394 | 1.24E-17 | 0.502 | 0.272 | 0.230 | 2.68E-17 |  |  |  |  |
| **FAM115A (TCAF1)** |  |  |  |  |  |  |  |  |  |  | 0.488 | 0.019 | 0.126 | 0.656 |
|  | **1** | cg03514404 | 0.740 | 0.077 | 0.662 | 8.14E-08 | 0.635 | 0.142 | 0.493 | 2.79E-32 |  |  |  |  |
|  | **2** | cg01030534 | 0.793 | 0.121 | 0.671 | 5.48E-18 | 0.674 | 0.170 | 0.504 | 4.00E-38 |  |  |  |  |
|  | **3** | cg03225210 | 0.660 | 0.094 | 0.567 | 5.98E-16 | 0.598 | 0.143 | 0.455 | 5.13E-30 |  |  |  |  |
|  | **4** | cg02245020 | 0.765 | 0.060 | 0.705 | 7.35E-18 | 0.630 | 0.206 | 0.424 | 5.83E-30 |  |  |  |  |
|  | **5** | cg09671837 | 0.714 | 0.225 | 0.489 | 3.56E-12 | 0.679 | 0.439 | 0.240 | 7.89E-19 |  |  |  |  |
|  | **6** | cg16807089 | 0.708 | 0.095 | 0.614 | 2.04E-16 | 0.640 | 0.288 | 0.352 | 4.84E-25 |  |  |  |  |
|  | **7** | cg09846875 | 0.559 | 0.100 | 0.459 | 1.47E-13 | 0.616 | 0.357 | 0.259 | 3.33E-18 |  |  |  |  |
| **HLF** |  |  |  |  |  |  |  |  |  |  | -1.209 | 3.66E-06 | -1.106 | 9.46E-13 |
|  | **1** | cg20640281 | 0.698 | 0.457 | 0.240 | 2.45E-06 | 0.661 | 0.567 | 0.094 | 2.97E-05 |  |  |  |  |
|  | **2** | cg04219321 | 0.528 | 0.325 | 0.203 | 2.84E-03 | 0.574 | 0.471 | 0.103 | 1.53E-04 |  |  |  |  |
|  | **3** | cg18943957 | 0.381 | 0.132 | 0.248 | 1.77E-05 | 0.371 | 0.203 | 0.168 | 2.15E-04 |  |  |  |  |
|  | **4** | cg01392772 | 0.554 | 0.036 | 0.518 | 6.86E-17 | 0.499 | 0.177 | 0.322 | 1.04E-15 |  |  |  |  |
|  | **5** | cg05452524 | 0.609 | 0.030 | 0.579 | 5.85E-15 | 0.526 | 0.096 | 0.431 | 8.29E-22 |  |  |  |  |
|  | **6** | cg15835232 | 0.601 | 0.060 | 0.541 | 1.69E-18 | 0.538 | 0.214 | 0.324 | 9.43E-25 |  |  |  |  |
|  | **7** | cg02383154 | 0.524 | 0.076 | 0.448 | 8.71E-17 | 0.513 | 0.166 | 0.348 | 1.24E-33 |  |  |  |  |
|  | **8** | cg01185682 | 0.495 | 0.073 | 0.422 | 1.44E-13 | 0.487 | 0.147 | 0.339 | 1.86E-32 |  |  |  |  |
|  | **9** | cg01451391 | 0.646 | 0.077 | 0.569 | 4.74E-14 | 0.606 | 0.174 | 0.431 | 1.90E-34 |  |  |  |  |
| **LOC149134 (LINC01341)** |  |  |  |  |  |  |  |  |  |  | NA | NA | NA | NA |
|  | **1** | cg05314024 | 0.556 | 0.198 | 0.358 | 6.88E-13 | 0.539 | 0.411 | 0.128 | 4.58E-08 |  |  |  |  |
|  | **2** | cg15176829 | 0.758 | 0.161 | 0.597 | 6.41E-17 | 0.637 | 0.339 | 0.298 | 2.27E-25 |  |  |  |  |
|  | **3** | cg03596016 | 0.812 | 0.158 | 0.654 | 2.43E-17 | 0.698 | 0.201 | 0.497 | 2.20E-36 |  |  |  |  |
|  | **4** | cg08535928 | 0.661 | 0.163 | 0.498 | 9.69E-16 | 0.634 | 0.223 | 0.412 | 1.64E-36 |  |  |  |  |
|  | **5** | cg03340119 | 0.507 | 0.118 | 0.389 | 2.16E-16 | 0.452 | 0.179 | 0.273 | 1.00E-26 |  |  |  |  |
| **LRRC4** |  |  |  |  |  |  |  |  |  |  | -0.455 | 0.299 | -1.067 | 2.42E-07 |
|  | **1** | cg06173720 | 0.570 | 0.327 | 0.243 | 2.18E-04 | 0.574 | 0.479 | 0.095 | 1.27E-04 |  |  |  |  |
|  | **2** | cg26173847 | 0.461 | 0.072 | 0.389 | 1.47E-08 | 0.400 | 0.096 | 0.304 | 4.17E-11 |  |  |  |  |
|  | **3** | cg26595520 | 0.332 | 0.043 | 0.289 | 8.20E-09 | 0.405 | 0.183 | 0.222 | 5.71E-10 |  |  |  |  |
|  | **4** | cg00353923 | 0.498 | 0.084 | 0.414 | 2.62E-08 | 0.497 | 0.235 | 0.262 | 2.83E-12 |  |  |  |  |
|  | **5** | cg07871590 | 0.467 | 0.053 | 0.414 | 1.89E-10 | 0.414 | 0.090 | 0.324 | 2.92E-13 |  |  |  |  |
|  | **6** | cg06945399 | 0.329 | 0.039 | 0.290 | 7.95E-10 | 0.379 | 0.162 | 0.216 | 8.61E-11 |  |  |  |  |
|  | **7** | cg27573591 | 0.340 | 0.023 | 0.316 | 1.72E-10 | 0.386 | 0.132 | 0.254 | 1.97E-13 |  |  |  |  |
|  | **8** | cg15087147 | 0.276 | 0.022 | 0.254 | 2.86E-12 | 0.400 | 0.137 | 0.262 | 6.32E-15 |  |  |  |  |
|  | **9** | cg04153784 | 0.345 | 0.016 | 0.329 | 2.08E-06 | 0.355 | 0.075 | 0.279 | 7.88E-15 |  |  |  |  |
|  | **10** | cg12628196 | 0.630 | 0.148 | 0.482 | 5.08E-13 | 0.609 | 0.206 | 0.403 | 4.42E-40 |  |  |  |  |
|  | **11** | cg09087503 | 0.523 | 0.154 | 0.368 | 6.46E-09 | 0.517 | 0.214 | 0.304 | 3.35E-39 |  |  |  |  |
|  | **12** | cg09296001 | 0.751 | 0.123 | 0.628 | 6.22E-16 | NA | NA | NA | NA |  |  |  |  |
|  | **13** | cg12345672 | 0.507 | 0.237 | 0.270 | 4.81E-10 | NA | NA | NA | NA |  |  |  |  |
| **PROM1** |  |  |  |  |  |  |  |  |  |  | -2.705 | 2.26E-07 | -1.182 | 3.11E-04 |
|  | **1** | cg22867816 | 0.753 | 0.484 | 0.269 | 8.60E-09 | 0.750 | 0.596 | 0.154 | 3.73E-14 |  |  |  |  |
|  | **2** | cg12839172 | 0.573 | 0.063 | 0.510 | 1.21E-12 | 0.465 | 0.113 | 0.353 | 1.61E-16 |  |  |  |  |
|  | **3** | cg13164157 | 0.524 | 0.025 | 0.500 | 1.37E-14 | 0.431 | 0.144 | 0.287 | 7.87E-13 |  |  |  |  |
|  | **4** | cg07817686 | 0.478 | 0.073 | 0.404 | 2.03E-15 | 0.496 | 0.236 | 0.259 | 3.64E-17 |  |  |  |  |
|  | **5** | cg09024126 | 0.668 | 0.127 | 0.541 | 6.48E-16 | 0.586 | 0.223 | 0.363 | 1.61E-31 |  |  |  |  |
|  | **6** | cg14736058 | 0.716 | 0.177 | 0.539 | 3.34E-16 | 0.628 | 0.249 | 0.379 | 9.15E-35 |  |  |  |  |
|  | **7** | cg10630155 | 0.582 | 0.143 | 0.439 | 1.93E-15 | 0.555 | 0.339 | 0.217 | 3.29E-19 |  |  |  |  |
|  | **8** | cg04203238 | 0.720 | 0.130 | 0.590 | 2.48E-17 | 0.612 | 0.213 | 0.398 | 2.60E-28 |  |  |  |  |
|  | **9** | cg26260038 | 0.817 | 0.184 | 0.633 | 9.42E-17 | 0.670 | 0.380 | 0.290 | 8.68E-25 |  |  |  |  |
|  | **10** | cg21647189 | 0.463 | 0.246 | 0.216 | 4.43E-10 | 0.482 | 0.335 | 0.147 | 1.23E-08 |  |  |  |  |
|  | **11** | cg08855742 | 0.709 | 0.485 | 0.224 | 2.00E-07 | 0.674 | 0.589 | 0.085 | 1.74E-05 |  |  |  |  |
| **RHCG** |  |  |  |  |  |  |  |  |  |  | -3.596 | 1.82E-04 | -1.930 | 3.28E-06 |
|  | **1** | cg02805106 | 0.410 | 0.137 | 0.272 | 2.87E-13 | 0.497 | 0.322 | 0.176 | 4.82E-15 |  |  |  |  |
|  | **2** | cg24653181 | 0.501 | 0.100 | 0.400 | 2.06E-15 | 0.579 | 0.329 | 0.250 | 1.12E-22 |  |  |  |  |
|  | **3** | cg18726691 | 0.733 | 0.097 | 0.636 | 3.14E-17 | 0.647 | 0.321 | 0.326 | 3.61E-27 |  |  |  |  |
|  | **4** | cg27507295 | 0.607 | 0.119 | 0.488 | 1.04E-16 | 0.551 | 0.303 | 0.248 | 1.86E-21 |  |  |  |  |
|  | **5** | cg07485916 | 0.643 | 0.126 | 0.517 | 1.23E-16 | 0.571 | 0.314 | 0.257 | 7.97E-23 |  |  |  |  |
|  | **6** | cg10721782 | 0.669 | 0.132 | 0.537 | 5.64E-17 | 0.585 | 0.327 | 0.258 | 1.03E-23 |  |  |  |  |
|  | **7** | cg01837657 | 0.740 | 0.120 | 0.620 | 5.26E-16 | 0.619 | 0.186 | 0.433 | 2.22E-35 |  |  |  |  |
|  | **8** | cg10453365 | 0.573 | 0.179 | 0.394 | 7.66E-17 | 0.626 | 0.408 | 0.218 | 4.95E-23 |  |  |  |  |
|  | **9** | cg12042113 | 0.521 | 0.092 | 0.429 | 3.86E-18 | 0.564 | 0.296 | 0.267 | 1.26E-24 |  |  |  |  |

**Table S3: Methylation and expression data for selected candidates.** Overview of DMCs associated to each selected candidate gene (mean Δβ>|0.2|, adj. *P*<0.05) including 450K methylation level for each probe, and methylation level according to TCGA 450K data, combined with expression data for each gene according to in-house and TCGA RNA-seq data. ^a^Probe numbers in fig. S2 and fig. S3. NA: Data not found in the respective dataset.

|  | **Cohort 1 (203 T *vs.* 30 NM)** | | | | **DNBs (25 T *vs.* 50 NM)** | | | |
| --- | --- | --- | --- | --- | --- | --- | --- | --- |
| **Candidate** | **AUC (95% CI)** | **Sensitivity (%)** | **Specificity (%)^a^** | ***P*-value^b^** | **AUC (95% CI)** | **Sensitivity (%)** | **Specificity (%)^a^** | ***P*-value^b^** |
| ***COL4A6*** | 0.89 (0.85 - 0.93) | 77.3 | 96.7 | <0.001 | 0.98 (0.94 - 1.00) | 96.0 | 96.0 | <0.001 |
| ***CYBA*** | 0.82 (0.77 - 0.87) | 66.0 | 96.7 | <0.001 | 0.97 (0.93 - 1.00) | 92.0 | 96.0 | <0.001 |
| ***HLF*** | 0.79 (0.74 - 0.85) | 52.2 | 96.7 | <0.001 | 0.97 (0.93 - 1.00) | 88.0 | 96.0 | <0.001 |
| ***LINC01341*** | 0.91 (0.86 - 0.95) | 85.7 | 96.7 | <0.001 | 0.98 (0.96 - 1.00) | 88.0 | 96.0 | <0.001 |
| ***LRRC4*** | 0.80 (0.75 - 0.84) | 63.1 | 96.7 | <0.001 | 0.98 (0.93 - 1.00) | 96.0 | 96.0 | <0.001 |
| ***PROM1*** | 0.89 (0.85 - 0.93) | 74.9 | 96.7 | <0.001 | 1.00 (1.00 - 1.00) | 94.0 | 96.0 | <0.001 |
| ***RHCG*** | 0.91 (0.87 - 0.94) | 78.8 | 96.7 | <0.001 | 0.99 (0.98 - 1.00) | 92.0 | 96.0 | <0.001 |
| ***TCAF1*** | 0.90 (0.86 - 0.94) | 82.8 | 96.7 | <0.001 | 1.00 (0.99 - 1.00) | 96.0 | 96.0 | <0.001 |

**Table S4:** **Diagnostic potential of candidate methylation markers.** Data from ROC analysis of T *vs.* NM samples (RP cohort 1), and DNBs. ^a^Specificities are fixed at different values (96.7% and 96.0%, respectively) due to data-dependent values automatically generated in STATA. For each dataset, the value closest to 95% was selected based on the exact distribution of patients in each cohort. ^b^*P*-values from rank-sum test.

|  | **Dichotomized post-operative GS (≤7<)^a^** | | **Dichotomized pT (≤2<)^a^** | | **PSA^b^** | | **Age^b^** | |
| --- | --- | --- | --- | --- | --- | --- | --- | --- |
|  | **cohort 1** | **cohort 2** | **cohort 1** | **cohort 2** | **cohort 1** | **cohort 2** | **cohort 1** | **cohort 2** |
| ***COL4A6*** | **0.005** | 0.131 | **0.003** | **<0.001** | **0.23 (0.001)** | **0.19 (0.001)** | -0.10 (0.174) | -0.07 (0.259) |
| ***CYBA*** | 0.077 | 0.916 | 0.133 | **0.003** | **0.20 ( 0.005)** | 0.04 (0.544) | -0.07 (0.328) | -0.06 (0.323) |
| ***HLF*** | 0.474 | 0.074 | **0.005** | **0.001** | **0.22 (0.001)** | **0.16 (0.006)** | 0.02 (0.747) | 0.02 (0.738) |
| ***LINC01341*** | 0.173 | **0.038** | 0.329 | **0.001** | 0.07 (0.353) | **0.14 (0.016)** | -0.13 (0.061) | -0.09 (0.130) |
| ***LRRC4*** | 0.441 | **0.021** | **0.002** | **0.000** | 0.08 (0.259) | **0.12 (0.043)** | 0.08 (0.229) | -0.03 (0.558) |
| ***PROM1*** | **0.006** | 0.307 | **0.005** | **0.000** | **0.16 (0.025)** | 0.11 (0.055) | 0.01 (0.939) | -0.09 (0.150) |
| ***RHCG*** | 0.075 | 0.368 | **0.011** | **0.001** | **0.23 ( 0.001)** | 0.09 (0.118) | -0.08 (0.253) | 0.02 (0.727) |
| ***TCAF1*** | 0.171 | **0.014** | **<0.001** | **<0.001** | **0.20 ( 0.004)** | **0.15 (0.013)** | -0.13 (0.060) | 0.02 (0.789) |

**Table S5: Association between methylation marker candidates and clinicopathological parameters.** Bold: *P*<0.05. ^a^Wilcoxon rank-sum test *P*-value. ^b^Spearman's rho (*P*-value).

| Training cohort (*N*=203) | | | | |
| --- | --- | --- | --- | --- |
| Univariate | | | | |
| Variable | HR (95% CI) | C-index | *P* | adj. *P* |
| ***COL4A6* (cont.)** | 1.78 (1.25 - 2.53) | 0.601 | **0.001** | **0.006** |
| ***CYBA* (cont.)** | 12.2 (0.18 - 836) | 0.582 | 0.246 | 0.492 |
| ***HLF* (cont.)** | 4.98 (0.52 - 47.8) | 0.609 | 0.164 | 0.492 |
| ***LINC01341* (cont.)** | 0.90 (0.50 - 1.61) | 0.470 | 0.725 | 0.725 |
| ***LRRC4* (cont.)** | 1.60 (0.84 - 3.04 | 0.567 | 0.151 | 0.604 |
| ***PROM1* (cont.)** | 4.88 (2.04 - 11.7) | 0.616 | **<0.001** | **<0.006** |
| ***RHCG* (cont.)** | 1.73 (1.26 - 2.37) | 0.639 | **0.001** | **0.006** |
| ***TCAF1* (cont.)** | 3.15 (2.05 - 4.85) | 0.663 | **<0.001** | **<0.006** |
| **Tumor stage (pT2 *vs.* pT3-4)** | 4.46 (2.86 - 6.97) | 0.677 | **<0.001** | **<0.001** |
| **Gleason score (<7 *vs.* ≥7)** | 2.09 (1.32 - 3.30) | 0.579 | **0.002** | **0.002** |
| **Pre-op. PSA (<10 *vs.* ≥10)** | 2.73 (1.59 - 4.71) | 0.600 | **<0.001** | **<0.001** |
| **Surgical margin (neg. *vs.* pos.)** | 3.47 (2.25 - 5.36) | 0.663 | **<0.001** | **<0.001** |
|  |  |  |  |  |
|  |  |  |  |  |
| Validation cohort (*N*=286) | | | | |
| Univariate | | | | |
| Variable | HR (95% CI) | C-index | *P* | adj. *P* |
| ***COL4A6* (cont.)** | 1.25 (1.06 - 1.48) | 0.610 | **0.008** | **0.008** |
| ***PROM1* (cont.)** | 1.54 (1.28 - 1.86) | 0.594 | **<0.001** | **<0.004** |
| ***RHCG* (cont.)** | 1.47 (1.20 - 1.8) | 0.610 | **<0.001** | **<0.004** |
| ***TCAF1* (cont.)** | 1.44 (1.20 - 1.74) | 0.600 | **<0.001** | **<0.004** |
| **Tumor stage (pT2 *vs.* pT3-4)** | 3.16 (2.09 - 4.78) | 0.629 | **<0.001** | **<0.001** |
| **Gleason score (<7 *vs.* ≥7)** | 3.53 (2.15 - 5.81) | 0.638 | **<0.001** | **<0.001** |
| **Pre-op. PSA (<10 *vs.* ≥10)** | 2.51 (1.66 - 3.79) | 0.593 | **<0.001** | **<0.001** |

**Table S6:** **Univariate Cox regression analysis of methylation markers as continuous variables.** Analyses in cohort 1 (top) and 2 (bottom). Only candidate genes significant in cohort 1 (training) were further tested in cohort 2 (validation). Bold: *P*<0.05. Adj. *P*: *P*-value adjusted according to Hochberg [35].

| Training cohort (*N*=203) | | | | |
| --- | --- | --- | --- | --- |
| Univariate | | | | |
| Variable | HR (95% CI) | C-index | *P* | adj. *P* |
| ***RHCG* (dichotomized)** | 2.29 (1.49 - 3.52) | 0.5983 | **<0.001** | **<0.002** |
| ***TCAF1* (dichotomized)** | 2.75 (1.75 - 4.32) | 0.5988 | **<0.001** | **<0.002** |
| **Tumor stage (pT2 *vs*. pT3-4)** | 4.46 (2.86 - 6.97) | 0.677 | **<0.001** | **<0.001** |
| **Gleason score (<7 *vs.* ≥7)** | 2.09 (1.32 - 3.30) | 0.579 | **0.002** | **0.002** |
| **Pre-op. PSA (<10 *vs.* ≥10)** | 2.73 (1.59 - 4.71) | 0.600 | **<0.001** | **<0.001** |
| **Surgical margin (neg. *vs.* pos.)** | 3.47 (2.25 - 5.36) | 0.663 | **<0.001** | **<0.001** |
|  |  |  |  |  |
| Validation cohort (*N*=286) | | | | |
| Univariate | | | | |
| Variable | HR (95% CI) | C-index | *P* | adj. *P* |
| ***RHCG* (dichotomized)** | 1.70 (1.11 - 2.59) | 0.565 | **0.014** | **0.014** |
| ***TCAF1* (dichotomized)** | 2.00 (1.23 - 3.26) | 0.557 | **0.005** | **0.010** |
| **Tumor stage (pT2 *vs.* pT3-4)** | 3.16 (2.09 - 4.78) | 0.629 | **<0.001** | **<0.001** |
| **Gleason score (<7 *vs.* ≥7)** | 3.53 (2.15 - 5.81) | 0.638 | **<0.001** | **<0.001** |
| **Pre-op. PSA (<10 *vs.* ≥10)** | 2.51 (1.66 - 3.79) | 0.593 | **<0.001** | **<0.001** |

**Table S7:** **Univariate Cox regression analysis of *RHCG* and *TCAF1* analyzed as dichotomized variables.** Analyses in cohort 1 (top) and 2 (bottom). Bold: *P*<0.05.

| Training cohort (*N*=203) | | | | | |
| --- | --- | --- | --- | --- | --- |
| Multivariate | | | | | |
| Variable | HR (95% CI) | *P* | adj. *P* | C-index^a^ | C-index^b^ |
| ***RHCG* (dichotomized)** | 1.75 (1.13 - 2.73) | **0.013** | **0.026** | **0.777** |  |
| **Tumor stage (pT2 *vs.* pT3-4)** | 3.03 (1.84 - 4.99) | **<0.001** | **<0.001** |  | **0.769** |
| **Gleason score (<7 *vs.* ≥7)** | 1.83 (1.15 - 2.90) | **0.010** | **0.010** |  |  |
| **Pre-op. PSA (<10 *vs.* ≥10)** | 2.54 (1.47 - 4.40) | **0.001** | **0.001** |  |  |
| **Surgical margin (neg. *vs.* pos.)** | 2.25 (1.37 - 3.68) | **0.001** | **0.001** |  |  |
|  |  |  |  |  |  |
| Validation cohort (*N*=286) | | | | | |
| Multivariate | | | | | |
| Variable | HR (95% CI) | *P* | adj. *P* | C-index^a^ | C-index^b^ |
| ***RHCG* (dichotomized)** | 1.74 (1.13 - 2.67) | **0.012** | **0.024** | **0.714** |  |
| **Tumor stage (pT2 *vs.* pT3-4)** | 2.02 (1.31 - 3.13) | **0.001** | **0.001** |  | **0.703** |
| **Gleason score (<7 *vs.* ≥7)** | 2.93 (1.75 - 4.90) | **<0.001** | **<0.001** |  |  |
| **Pre-op. PSA (<10 *vs.* ≥10)** | 1.70 (1.11 - 2.61) | **0.014** | **0.014** |  |  |

**Table S8:** **Multivariate Cox regression analysis of *RHCG* (dichotomized) and clinicopathological variables.** Analyses in cohort 1 (top) and 2 (bottom). Bold: *P*<0.05. ^a^Model including all variables significant in multivariate analysis. ^b^Model including clinicopathological variables only.

| Training cohort (*N*=203) | | | | | |
| --- | --- | --- | --- | --- | --- |
| Multivariate | | | | | |
| Variable | HR (95% CI) | *P* | adj. *P* | C-index^a^ | C-index^b^ |
| ***TCAF1* (dichotomized)** | 1.65 (1.01 - 2.67) | **0.044** | **0.044** | **0.777** |  |
| **Tumor stage (pT2 *vs.* pT3-4)** | 2.70 (1.61 - 4.54) | **<0.001** | **<0.001** |  | **0.769** |
| **Gleason score (<7 *vs.* ≥7)** | 1.80 (1.13 - 2.85) | **0.013** | **0.013** |  |  |
| **Pre-op. PSA (<10 *vs.* ≥10)** | 2.67 (1.54 - 4.62) | **<0.001** | **<0.001** |  |  |
| **Surgical margin (neg. *vs.* pos.)** | 2.32 (1.43 - 3.77) | **0.001** | **0.001** |  |  |
|  |  |  |  |  |  |
| Validation cohort (*N*=286) | | | | | |
| Multivariate | | | | | |
| Variable | HR (95% CI) | *P* | adj. *P* | C-index^a^ | C-index^b^ |
| ***TCAF1* (dichotomized)** | 1.70 (1.03 - 2.80) | **0.038** | **0.038** | **0.714** |  |
| **Tumor stage (pT2 *vs.* pT3-4)** | 2.06 (1.33 - 3.18) | **0.001** | **0.001** |  | **0.703** |
| **Gleason score (<7 *vs.* ≥7)** | 2.76 (1.65 - 4.61) | **<0.001** | **<0.001** |  |  |
| **Pre-op. PSA (<10 *vs.* ≥10)** | 1.66 81.08 - 2.54) | **0.021** | **0.021** |  |  |

**Table S9:** **Multivariate Cox regression analysis of *TCAF1* (dichotomized) and clinicopathological variables.** Analyses in cohort 1 (top) and 2 (bottom). Bold: *P*<0.05. ^a^Model including all variables significant in multivariate analysis. ^b^Model including clinicopathological variables only.

| **Training cohort (*N*=203)** | | | | | | | |
| --- | --- | --- | --- | --- | --- | --- | --- |
|  | Univariate | | |  | Multivariate |  |  |
| Variable | HR (95% CI) | *P* | C-index | HR (95% CI) | *P* | C-index^a^ | C-index^b^ |
| **t*RHCG-TCAF1* (low *vs.* intermediate)** | 2.00 (1.19 - 3.36) | **0.008** | 0.629 | 1.25 (0.72 - 2.17) | 0.421 | **0.780** |  |
| **t*RHCG-TCAF1* (low *vs.* high)** | 3.50 (2.07 - 5.91) | **<0.001** |  | 2.28 (1.31 - 3.96) | **0.003** |  |  |
| **Tumor stage (pT2 *vs.* pT3-4)** | 4.46 (2.86 - 6.97) | **<0.001** | 0.677 | 2.69 (1.61 - 4.48) | **<0.001** |  | **0.769** |
| **Gleason score (<7 *vs.* ≥7)** | 2.09 (1.32 - 3.30) | **0.002** | 0.579 | 1.84 (1.16 - 2.92) | **0.010** |  |  |
| **Pre-op. PSA (<10 *vs.* ≥10)** | 2.73 (1.59 - 4.71) | **<0.001** | 0.600 | 2.67 (1.53 - 4.63) | **<0.001** |  |  |
| **Surgical margin (neg. *vs*. pos.)** | 3.47 (2.25 - 5.36) | **<0.001** | 0.663 | 2.38 (1.44 - 3.93) | **0.001** |  |  |
|  |  |  |  |  |  |  |  |
|  |  |  |  |  |  |  |  |
| **Validation cohort (*N*=286)** | | | | | | | |
|  | Univariate | | | Multivariate | | | |
| Variable | HR (95% CI) | *P* | C-index | HR (95% CI) | *P* | C-index^a^ | C-index^b^ |
| **t*RHCG-TCAF1* (low *vs.* intermediate)** | 1.41 (0.854 - 2.31) | 0.180 | 0.579 | 1.57 (0.981 - 2.52) | 0.060 | **0.719** |  |
| **t*RHCG-TCAF1* (low *vs.* high)** | 2.40 (1.39 - 4.14) | **0.002** |  | 2.02 (1.15 - 3.54) | **0.015** |  |  |
| **Tumor stage (pT2 *vs.* pT3-4)** | 3.16 (2.09 - 4.78) | **<0.001** | 0.629 | 1.98 (1.27 - 3.07) | **0.002** |  | **0.703** |
| **Gleason score (<7 *vs.* ≥7)** | 3.53 (2.15 - 5.81) | **<0.001** | 0.638 | 2.88 (1.72 - 4.81) | **<0.001** |  |  |
| **Pre-op. PSA (<10 *vs.* ≥10)** | 2.51 (1.66 - 3.79) | **<0.001** | 0.593 | 1.71 (1.11 - 2.63) | **0.015** |  |  |

**Table S10**: **Uni- and multivariate Cox regression analysis of the trichotomized *RHCG-TCAF1* panel.** Analyses in cohort 1 (top) and 2 (bottom). Bold: *P*<0.05. ^a^Model including all variables significant in multivariate analysis. ^b^Model including clinicopathological variables only.

| **Cell line** | **Phenotype** | **Provider** | **Culture conditions** |
| --- | --- | --- | --- |
| 22rv1 | Malignant | ATCC | RPMI 1640 with 10% FBS (Invitrogen) and 1% penicillin/streptomycin. |
| BPH1 | NM | German Collection of Microorganisms and Cell Cultures | RPMI 1640 with 10% FBS (Invitrogen) and 1% penicillin/streptomycin. |
| LNCaP | Malignant | ATCC | RPMI 1640 with 10% FBS (Invitrogen) and 1% penicillin/streptomycin. |
| PC3 | Malignant | ATCC | RPMI 1640 with 10% FBS (Invitrogen) and 1% penicillin/streptomycin. |
| PrEC | NM | ATCC | Prostate Epithelial Cell Basal Medium (ATCC) plus contents of the Prostate Epithelial Cell Growth Kit (ATCC). |

**Table S11:** **Prostate cell lines included in 450K and BS analysis.** ATCC: American Type Culture Collection.

|  | **Cohort 1** | | **Cohort 2** | |
| --- | --- | --- | --- | --- |
|  | **Complete** | **Final** | **Complete** | **Final** |
| *N* | **565** | **203** | **565** | **286** |
| Age at RP, median (range) | 64 (36 - 77) | 63 (47 - 77) | 61 (41 - 82) | 61 (41 - 76) |
| Unknown | 0 (0.0 %) | 0 (0.0 %) | 7 (1.2 %) | 5 (1.7 %) |
| **Pathological Gleason score** |  |  |  |  |
| <7, *N* (%) | 218 (38.6 %) | 96 (47.3 %) | 210 (37.2 %) | 125 (43.7 %) |
| =7, *N* (%) | 269 (47.6 %) | 85 (41.9 %) | 190 (33.6 %) | 135 (47.2 %) |
| >7, *N* (%) | 78 (13.8 %) | 22 (10.8 %) | 39 (6.9 %) | 26 (9.1 %) |
| Unknown, *N* (%) | 0 (0.0 %) | 0 (0.0 %) | 126 (22.3 %) | 0 (0.0 %) |
| **Pathological T-stage** |  |  |  |  |
| ≤pT2c, *N* (%) | 392 (69.4 %) | 131 (64.5 %) | 377 (66.7 %) | 216 (75.5 %) |
| ≥pT3a, *N* (%) | 172 (30.4 %) | 72 (35.5 %) | 114 (20.2 %) | 70 (24.5 %) |
| Unknown, *N* (%) | 1 (0.2 %) | 0 (0.0 %) | 74 (13.1 %) | 0 (0.0 %) |
| **Pre-operative PSA** |  |  |  |  |
| PSA ng/ml, median (range) | 11 (1.5 - 250) | 12.1 (2.0 - 61.0) | 6.6 (0.6 - 62.1) | 6.4 (0.6 - 62.1) |
| Unknown, *N* (%) | 0 (0.0 %) | 0 (0.0 %) | 214 (37.9 %) | 0 (0.0 %) |
| **Surgical margin status** |  |  |  |  |
| Negative, *N* (%) | 394 (69.7 %) | 140 (69.0 %) | 80 (14.2 %) | 72 (25.2 %) |
| Positive, *N* (%) | 168 (29.7 %) | 63 (31.0 %) | 37 (6.5 %) | 34 (11.9 %) |
| Unknown, *N* (%) | 3 (0.5 %) | 0 (0.0 %) | 448 (79.3 %) | 181 (63.3 %) |
| **Lymph node status** |  |  |  |  |
| Positive, *N* (%) | 6 (1.1 %) | 0 (0.0 %) | 6 (1.1 %) | 0 (0.0 %) |
| Negative, *N* (%) | 394 (69.7 %) | 24 (11.8 %) | 42 (7.4 %) | 23 (8.0 %) |
| Unknown, *N* (%) | 165 (29.2 %) | 179 (88.2 %) | 517 (91.5 %) | 263 (92.0 %) |
| **Median follow-up, months (range)** | 77 (1 - 184) | 70 (3 -184) | 79 (0.1 - 290) | 73 (3 - 290) |
| **No follow-up** | 2 (0.4 %) | 0 (0.0 %) | 1 (0.2 %) | 0 (0.0 %) |
| **PSA recurrence, *N* (%)** | 220 (38.9 %) | 85 (41.9 %) | 170 (30.1 %) | 92 (32.2 %) |
| **No PSA recurrence, *N* (%)** | 345 (61.1 %) | 118 (58.1 %) | 326 (57.7 %) | 194 (67.8 %) |
| **PSA recurrence unknown, *N* (%)** | 0 (0.0 %) | 0 (0.0 %) | 69 (12.2 %) | 0 (0.0 %) |

**Table S12: Clinicopathological characteristics for the complete (initial) and final RP cohorts 1 and 2.**

|  | **BS primers** | |  | **qMSP primers and probes** | | |
| --- | --- | --- | --- | --- | --- | --- |
| **Gene** | **Sense** | **Antisense** | **Sense (pmol/reaction)** | **Antisense (pmol/reaction)** | **Probe (pmol/reaction)** | **Reaction vol. & annealing temp. (z)** |
| aluC4^a^ | - | - | GGTTAGGTATAGTGGTTTATATTTGTAATTTTAGTA (5 pmol) | ATTAACTAAACTAATCTTAAACTCCTAACCTCA (5 pmol) | FAM_CCT ACC TTA ACC TCC C_MGBNFQ (2.04 pmol) | 10 µl, 58°C |
| *COL4A6* | TTGGATGTTTGTTTTTAATTTTTTTG | TCCATTCTTTCCTCTCCTACTACATAA | ATTTAGGGTGTATGTTTGCG (5 pmol) | CCAAATAAACACGAACCG (5 pmol) | FAM_TTCGGAGTTGGGTTTCGGGA_BHQ1 (1.7 pmol) | 5 µl, 53°C |
| *CYBA* | TTATTGGGGGTTTTAGAATTTTTTT | AACAAAACCCCTCCTATACCTC | TTTTCGGTCGAATTTCGTTT (5 pmol) | CTCCGAAACAACCCTACACC (5 pmol) | FAM_CGCGGTTGGGCGGAGATTCG_BHQ1 (3 pmol) | 10 µl, 60° |
| *HLF* | GGGGTTGTGTTATAGTTTTTGTTATT | ACTAAAACCAAAATTATCTTAAAAAAATT | AAGGGTAGTTGAGAAAGCG (5 pmol) | ACGCTAAAAACGTACACGA (5 pmol) | FAM_GAGTGGTTTTTCGGCGGGAA_BHQ1  (3 pmol) | 10 µl, 60° |
| *LINC01341* | TTGTTGGGTTTGTAGAAGCAGTGGT | AATAAATCTACCCACTTTCCACCAA | GTAGAGATCGGTAGCGGC (5 pmol) | AAAATCTCCCCTCCTCG (5 pmol) | FAM_GCGGTTCGCGTGGGTTATTG_BHQ1 (1.7 pmol) | 5 µl, 55°C |
| *LRRC4* | GGGGGAGATAAAATGGTTTTTAGTA | ATACCAAAATCCAAAAACAAAACC | TTTAGTAAATTCGGAGTCGG (5 pmol) | CATACACCTACATAACCCGC (5 pmol) | FAM_GCGGAGTTGAGGCGCGTTTG_BHQ1 (1.7 pmol) | 5 µl, 53°C |
| *MYOD1^b^* | - | - | CCAACTCCAAATCCCCTCTCTAT (3 pmol) | TGGTTTTTTTAGGGAGTAAGTTTGTT (3 pmol) | FAM_TCCCTTCCTATTCCTAAATCCAACCTAAATACCTCC_BHQ1 (1 pmol) | 10 µl, 56°C |
| *PROM1* | TATTTTTAGTATAGTGGAAGGAGTG | ACACCTAACCATACTCTCAACTCTC | AGTATAGTGGAAGGAGTGCG (10 pmol) | CGAAAACCGAATACACGA (10 pmol) | FAM_TTTTTCGTTTCGGGATGAGGATTTAG_BHQ1 (3.4 pmol) | 10 µl, 56°C |
| *RHCG* | GGAAAAGGAGTTTGTGTATATTTGATAAT | AAACACCCCTACAACATAACCTAAA | TATGTTTTGTAGAGGTCGGC (5 pmol) | CCACCTAACCAAAAACTCG (5 pmol) | FAM_AGATGCGGAGTTCGCGGTTG_BHQ1 (2.04 pmol) | 10 µl, 58°C |
| *TCAF1* | TAGAATTTGGAGAGGAGGAGTAATG | CTAAAACTCCCACTCCAACCTAAA | AGAGGAAAATTATTAGTAGAAATACGG (5 pmol) | TACGAAACGCGATAAATACG (5 pmol) | FAM_TTTGTTTTCGATGATTTTTGATTTCGG_BHQ1 (2.04 pmol) | 10 µl, 58°C |

**Table S13: Details of BS and qMSP assays.** Primer and probe sequences for BS and qMSP assays, in addition to qMSP reaction conditions. PCR program: 10 min 95°C, 50x (15 s 95°C, 1 min z°C,30 s 72°C), z = annealing temperature, as specified in the table. ^a^Primer/probe sequence from Weisenberger *et al.* [34]. ^b^Primer/probe sequence from Haldrup *et al.* [8]. For pre-amplification of DNA from DNBs, 10 ng bisulfite converted DNA was amplified in a 15 cycles, 15 µl pre-amplification reaction (C1000 Thermal Cycler, Bio-Rad) in 96-well plates . Two µl pre-amplified DNA was used as template in qMSP analysis.
